# Proteomic responses of two spring wheat cultivars to the combined water deficit and aphid (*Metopolophium dirhodum*) treatments

**DOI:** 10.3389/fpls.2022.1005755

**Published:** 2022-11-14

**Authors:** Klára Kosová, Pavel Vítámvás, Jiří Skuhrovec, Jan Vítámvás, Sébastien Planchon, Jenny Renaut, Pavel Saska

**Affiliations:** ^1^ Plant Stress Biology and Biotechnology Group, Department of Plant Genetics and Breeding, Crop Research Institute, Prague, Czechia; ^2^ Functional Diversity Group, Department of Plant Protection, Crop Research Institute, Prague, Czechia; ^3^ Faculty of Forestry and Wood Science, Czech University of Life Sciences, Prague, Czechia; ^4^ Biotechnologies and Environmental Analytics Platform (BEAP), Environmental Research and Innovation (ERIN) Department, Luxembourg Institute of Science and Technology (LIST), Esch-sur-Alzette, Luxembourg

**Keywords:** spring wheat, water deficit, herbivorous insects, combined stress, proteome, 2D-DIGE

## Abstract

In the field, plants usually have to face the combined effects of abiotic and biotic stresses. In our study, two spring wheat cultivars—Septima and Quintus—were subjected to three water regimes [70%, 50%, and 40% soil water capacity (SWC)], aphid (*Metopolophium dirhodum*) infestation, or the combination of both stresses, i.e., water deficit (50%, 40% SWC) and aphids. The study has a 2 × 3 × 2 factorial design with three biological replicates. In the present study, the results of proteomic analysis using 2D-DIGE followed by MALDI-TOF/TOF protein identification are presented. Water deficit but also aphid infestation led to alterations in 113 protein spots including proteins assigned to a variety of biological processes ranging from signaling *via* energy metabolism, redox regulation, and stress and defense responses to secondary metabolism indicating a long-term adaptation to adverse conditions. The absence of specific proteins involved in plant response to herbivorous insects indicates a loss of resistance to aphids in modern wheat cultivars during the breeding process and is in accordance with the “plant vigor hypothesis.” Septima revealed enhanced tolerance with respect to Quintus as indicated by higher values of morphophysiological characteristics (fresh aboveground biomass, leaf length, osmotic potential per full water saturation) and relative abundance of proteins involved in mitochondrial respiration and ATP biosynthesis.

## 1 Introduction

Common wheat represents one of the most important agricultural crops grown worldwide. Drought as water deficit in soil limiting plant growth and development and crop yield represents the most severe environmental stress globally (Chaves et al., 2003). In the field, plants usually have to face multiple stress factors including combinations of abiotic and biotic stresses (Mittler, 2006). Herbivorous insects such as aphids represent one of the major threats to commercial wheat production since high-yielding commercial wheat cultivars have lost natural resistance against herbivorous insects during the breeding process (van Emden and Harrington, 2017).

Proteins are directly involved in plant phenotypic responses to environmental cues including stresses (Kosová et al., 2018). The majority of proteomics studies on wheat are focused on plant response to a single stress factor only although it is evident that the effects of combined stress treatments do not simply equal to the sum of the effects of individual stress factors and that various stress interactions including both synergistic and antagonistic effects have to be taken into account (Mittler, 2006; Rasmussen et al., 2013). Recent knowledge on the impacts of combined abiotic and biotic stress factors on plants was reviewed by Suzuki et al. (2014) and Saijo and Po-iian Loo (2020).

Recently, a relatively large number of studies dealing with wheat proteome responses to drought have been published (Hajheidari et al., 2007; Caruso et al., 2009; Ford et al., 2011; Alvarez et al., 2014). Proteomics studies dealing with drought stress impacts on plants usually reported imbalances in cell redox homeostasis leading to enhanced ROS production and, as a response, to downregulation of redox reactions in energy metabolism, namely, photosynthesis (Hajheidari et al., 2007; Caruso et al., 2009). Recently, more than 100 papers corresponding to “plant proteome and aphid” have been found in the Web of Science database (accessed in June 2022). Aphids induced the accumulation of several defense modulators such as terpene synthase, papain-like cysteine protease, serine carboxypeptidase, and lipoxygenase and the repression of photosynthesis-related proteins in plants (Zogli et al., 2020). Nguyen et al. (2007) investigated the proteome of the aphid *Macrosiphum euphorbiae* rearing on potato (*Solanum tuberosum*) plants exposed to water deficit and defoliation by the beetle *Leptinotarsa decemlineata* and found enhanced levels of aphid proteins involved in cell communication, energy metabolism, and GroEL symbionin of the endosymbiont *Buchnera aphidicola* with chaperone function. However, to our best knowledge, only one study dealing with the proteome response of a commercial bread wheat cultivar to cereal aphid (*Sitobion avenae*) infestation in comparison to salicylic acid (SA) and methyl jasmonate (MeJA) treatments was published (Ferry et al., 2011). Finally, no study aimed at an investigation of wheat proteome response to water deficit combined with aphid infestation was published until now.

To fill this knowledge gap, we investigated the interaction between drought stress and aphids on plant response at the proteomic level. We used two spring wheat cultivars—Quintus (susceptible to water deficit) and Septima (tolerant to water deficit)—grown at three water regimes [70% soil water content (SWC) = control, well-watered plants; 50% SWC = mild water deficit; 40% SWC = relatively severe water deficit] combined with aphid (*Metopolophium dirhodum*) infestation versus non-infested control plants. Our study thus has a 2 × 3 × 2 factorial design with 12 experiment variants and 3 biological replicates. The plant response at the morphophysiological level and the aphid response at the population level were reported in our previous study (Saska et al., 2022) and are briefly summarized in **Supplementary Tables S1** and **S2**, respectively. In these conditions, we found a gradual decrease in leaf biomass osmotic potential at full saturation with an increasing level of water deficit in both cultivars, but the tolerant Septima seemed to be less affected (Saska et al., 2022). Aphids further increased the level of stress in all irrigation regimes except for the severe level of stress (Saska et al., 2022). The aphid population increased best on plants grown under optimum conditions, followed by plants well-adapted to drought (Saska et al., 2022). This is because these plants likely provided a good nutritional source due to the enhanced accumulation of osmolytes in leaf tissues.

In this paper, we investigate the interaction of water deficit stress, aphid infestation, and spring wheat at the proteomics level.

The three major effects are going to be investigated:

1. The effect of three water regimes (70% SWC = control, well-watered plants; 50% SWC = mild water deficit; 40% SWC = relatively severe water deficit)
2. The effect of aphid infestation in comparison with non-infested plants
3. The combined effect of water deficit and aphid rearing on spring wheat proteome

## 2 Materials and methods

### 2.1 Plant materials and treatments

To study the influence of water deficit stress on the spring cereal–aphid system, two spring wheat (*Triticum aestivum* Linnaeus) cultivars were used: the drought-resistant Septima and the standard (=non-resistant to drought) Quintus. Based on our previous study (Saska et al., 2021), these cultivars were chosen because they were similar in most leaf structural traits and also in suitability for aphids under the conditions of optimum watering. Therefore, these cultivars represent an ideal opportunity to study the potential difference in aphid response to water deficit stress in drought-tolerant and standard spring wheat, without bringing bias caused by their structural traits or different levels of suitability for aphids. Also, we prefer spring wheat over the dominantly grown winter wheat because spring wheat is potentially more sensitive to spring drought due to spring sowing. The source of the seeds is given in Saska et al. (2021).

Plant cultivation was described in our previous study (Saska et al., 2022). Briefly, seeds were germinated on moist filter paper in the dark at 20°C for 2 days. The germinated seeds were sown in field soil, eight seeds per pot (0.25 L). Plants were grown in a growth chamber (Tyler T-16/4, Budapest, Hungary) at 18°C, 16 h/8 h (light/dark) long-day photoperiod, and irradiance of 300 μmol m^−2^ s^−1^ and watered gravimetrically to attain the precise level of soil water capacity (SWC), where 100% SWC was calculated as *m*_100_, i.e., the weight of water held in 1 g of dried soil (70°C for at least 24 h) after its water saturation and draining freely for 48 h, while 0% SWC was determined as *m*_0_, i.e., the weight of the same soil amount after drying in an oven at 60°C to constant weight. The water content corresponding to the SWC levels used in the experiment was calculated as the difference between the pot weight corresponding to 100% and 0% SWC values, *m_x_* = (*m*_100_ − *m*_0_)/100 * *x* (%), where *x* is the required SWC. The following levels of SWC were used: 70% (optimum watering serving as control; hereafter SWC 70), 50% (mild water deficit treatment; SWC 50), and 40% (high water deficit treatment; SWC 40) until the second leaves fully developed. Although we are aware that 40% SWC is not a very high level of water deficit stress for wheat, we call it “high” in this paper to better contrast the water deficit treatments. According to our preliminary experiments, 40% SWC was the most severe level of water stress tolerated by aphids since aphids were leaving the plants if SWC dropped below 40%. Despite the seemingly small difference between the mild and high stress treatments in terms of the level of SWC, significant differences could be detected regarding structural and morphophysiological leaf traits (Saska et al., 2022).

The rose-grain aphid *M. dirhodum* (Walker) was used as an aphid stressor. The aphids came from the laboratory population maintained at the Crop Research Institute (Prague). This species is one of the three most damaging aphids on cereals in central Europe (Honěk et al., 2018). It is a holocyclic species in central Europe, alternating *Rosa* spp. (the primary host where sexual reproduction takes place) with *Poaceae*. It primarily infests the leaves, which may be the reason why it has gained relatively less attention compared to the species that infest ears, despite its economic importance (Honěk et al., 2018 and references therein). The plant treatment with aphids was described in our previous study (Saska et al., 2022).

### 2.2 Proteomic analysis

#### 2.2.1 Sample preparation and 2D-DIGE analysis

Three biological replicates of leaf samples were ground to a fine powder under liquid nitrogen using a mortar and pestle, and total proteins were extracted using the trichloroacetic acid (TCA)–phenol–acetone method as described by Wang et al. (2006). Finally, total proteins were precipitated by ammonium acetate, and protein pellets were rinsed with acetone, dried, and dissolved in lysis buffer (Bio-Rad manual). The lysis buffer was adjusted to pH 8.5 by adding diluted NaOH, and protein concentration was determined by the 2D Quant kit (GE Healthcare, Chicago, Illinois, USA). Thirty micrograms of protein sample was used for sample labeling using Cy3 or Cy5, and an additional 15 μg of protein sample was added to an internal standard as a mixture of all samples used in the experiment and labeled with Cy2. Protein minimal labeling by CyDyes was carried out according to the manual (GE Healthcare, Chicago, Illinois, USA). After stopping the protein labeling reaction by adding an excessive amount of lysine, rehydration buffer and 4.5 µl of 100× Bio-Lyte 3-10 ampholyte (Bio-Rad, Hercules, CA, USA) were added to the mixed samples to a final volume of 455 µl. The samples were then loaded on an IEF Cell tray using 24-cm linear IPG strips p*I* 5–8 (Bio-Rad, Hercules, CA, USA). The samples were subjected to passive rehydration for 14 h followed by isoelectric focusing using a rapid voltage slope until the final voltage of 10,000 V and a total of 75,000 Vh. After the IEF, the strips with the samples were equilibrated using equilibration buffer I with dithiothreitol followed by equilibration buffer II with iodoacetamide according to the manual (Bio-Rad, Hercules, CA, USA). The second dimension was carried out on 24 cm 12.5% SDS-PAGE gels using EttanDALTsix (GE Healthcare). The 2D-DIGE gels were scanned using PharosFX Fluorescent Imager (Bio-Rad, Hercules, CA, USA) using three wavelengths at a resolution of 600 dpi. Densitometric analysis of the 2D-DIGE gel images was carried out using the software PDQuest Advanced, multichannel application, version 8.0.1 (Bio-Rad, Hercules, CA, USA). Protein spot matching in the whole 2D-DIGE image set was edited manually using the Group Consensus Tool. Protein spot density in the individual samples was normalized at the mean density of the corresponding spot in the internal standard. Protein spots revealing significant quantitative (at least 1.5-fold change at the 0.05 level determined by Student’s *t*-test in MS Excel) or qualitative (presence/absence) differences in at least one of the 24 biologically relevant ratios—70 (S/Q), 50 (S/Q), 40 (S/Q); S (50/70), S (40/70), S (40/50); Q (50/70), Q (40/70), Q (40/50); 70 (Qm/Q), 50 (Qm/Q), 40 (Qm/Q); 70 (Sm/S), 50 (Sm/S), 40 (Sm/S); 70 (Sm/Qm), 50 (Sm/Qm), 40 (Sm/Qm); Sm (50/70), Sm (40/50), Sm (40/70); and Qm (50/70), Qm (40/50), Qm (40/70) (where S means Septima; Q means Quintus; “m” means aphid treatment with *M. dirhodum*; 40, 50, and 70 mean 40%, 50%, and 70% SWC)—were excised from preparative gels (1,000 μg of protein load, proteins stained with Coomassie Brilliant Blue) using ExQuest Spot Cutter (Bio-Rad) and sent to protein identification by MALDI-TOF/TOF.

#### 2.2.2 Protein identifications

Protein identifications were carried out using MALDI-TOF/TOF (5800, SCIEX, Prague, Czechia). Spots were treated with an EVO 2 liquid handling workstation (Tecan, Männedorf, Switzerland), washed twice for 20 min with 50 mM of ammonium bicarbonate in 50% methanol, and then dried with 75% acetonitrile (2 times, 20 min) before being incubated with 40 ng of trypsin in 20 mM of ammonium bicarbonate at 37°C for 6 h. Peptides were then extracted with 50% acetonitrile containing 0.1% TFA and air dried before spotting on the MALDI target with CHCA as matrix.

Proteins were searched against the NCBI database (www.ncbi.nlm.nih.gov) downloaded on 23 April 2021 using Triticeae as taxonomy (NCBI taxid 147389) and encompassing 518,563 sequences and 220,219,273 residues. The allowed protein modifications included carbamidomethylation of cysteine as a fixed modification and oxidation and dioxidation of tryptophan, oxidation of methionine, and tryptophan modification by kynurenine as variable modifications. Peptide mass tolerance was ±100 ppm and fragment mass tolerance was ±0.5 Da with a maximum of two missed cleavages. The criteria used for considering protein identification as significant based on the MS/MS score included at least 1 peptide with an ion score higher than the 2-fold identity threshold (46) or at least 2 peptides with a total ion score >60 (only considering peptides with a score >20).

In multiple protein identifications, the spots having two or more significantly identified proteins with a similar protein score (less than a 3-fold difference) were excluded from further evaluation.

### 2.3 Immunoblot analysis of dehydrin proteins

Hydrophilic proteins from finely ground crowns were extracted using 100 mM of Tris–HCl, pH 8.8, and a 5-min boiling step to enrich the dehydrin fraction. Dehydrins were precipitated using acetone with 1% mercaptoethanol, and dry precipitates were solubilized in Laemmli buffer, loaded onto 12.5% 1-D SDS-PAGE gels, and run at 10 mA (stacking gel) and 25 mA (resolving gel) according to standard procedures (Laemmli, 1970). The proteins were transferred to a nitrocellulose membrane in a tank blot; membranes were blocked in 3% non-fat dry milk and incubated with anti-dehydrin primary antibody (Enzo Lifesciences, Farmingdale, NY, USA) and alkaline phosphatase-conjugated secondary antibody (Bio-Rad, Hercules, CA, USA); and dehydrin proteins were visualized using BCIP/NBT reagents (Bio-Rad, Hercules, CA, USA). Membranes with dehydrin bands were scanned by a GS-800 densitometer (Bio-Rad, Hercules, CA, USA), and band density was quantified by QuantityOne version 4.6.2 (Bio-Rad, Hercules, CA, USA).

### 2.4 Bioinformatics and statistical analyses

The differentially abundant protein (DAP) spots were determined as those protein spots which reveal a significant difference defined as a minimum 1.5-fold change significant at the 0.05 level as determined by Student’s *t*-test (MS Excel) in at least one of the 24 biologically relevant ratios defined above. Dehydrin protein relative accumulation in the immunoblot was evaluated by ANOVA, LSD 0.05 test using STATISTICA, version 14 (TIBCO Inc., UK). Principal component analysis (PCA) of the *Z*-score-transformed relative abundances of the 113 DAP spots was performed using R software version 4.1.2. (R Core Team, 2022). Biplots were created using the ggbiplot function of the ggbiplot package (Vu, 2011). Cluster analysis was performed on the *Z*-score-transformed data of the 113 DAPs plus data on 6 morphophysiological characteristics (fresh aboveground biomass, leaf length, WSD, Ψπ, Ψπ100, Fv/Fm) from the paper of Saska et al. (2022) using Euclidean distance and Ward’s minimum criteria in PermutMatrix software (Caraux and Pinloche, 2005).

Functional annotation of the identified proteins was done using Gene Ontology (www.geneontology.org) using three kinds of annotation: cellular localization, molecular function, and biological process. Protein–protein interactions were analyzed using the STRING database (https://string-db.org; accessed 30 August 2022).

## 3 Results

Plants used for the experiments are shown in **Supplementary Figure S1**. It is evident that 50% and 40% SWC treatments led to reduced plant growth in comparison with 70% SWC treatments. The basic data on the plant morphophysiological characteristics and aphid populations extracted from our previous study (Saska et al., 2022) are provided in **Supplementary Tables S1** and **S2**, respectively. Increasing water deficit resulted in a decrease in fresh aboveground biomass and osmotic potential expressed per full water saturation Ψ100 in both cultivars (**Supplementary Table S1**) and led to a decrease in aphid fecundity (**Supplementary Table S2**).

**Figure 1:**
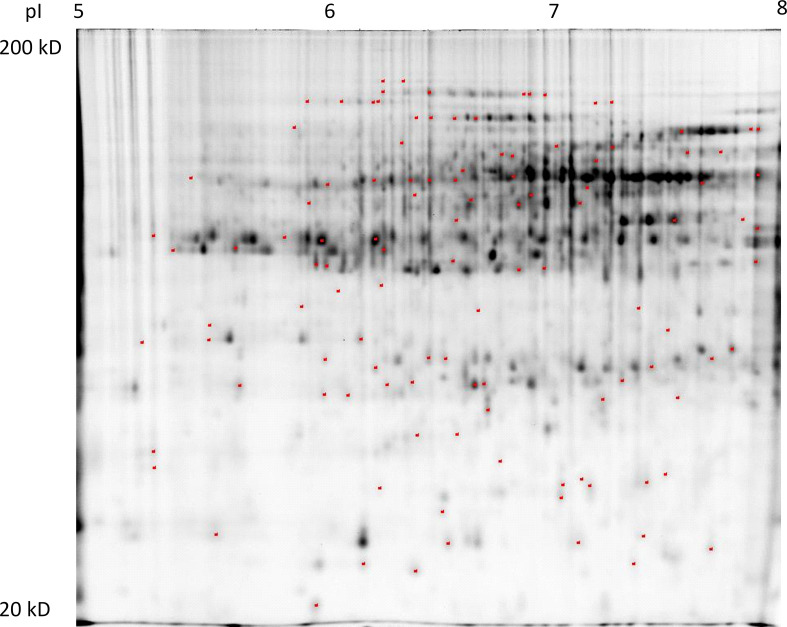
A representative 2D-DIGE gel (internal standard labeled by Cy2) with indicated positions of protein spots revealing differential relative abundance (at least 1.5-fold change at the 0.05 level) in at least one of 18 biologically relevant ratios: 70 (S/Q), 50 (S/Q), 40 (S/Q); S (50/70), S (40/70), S (40/50); Q (50/70), Q (40/70), Q (40/50); 70 (Qm/Q), 50 (Qm/Q), 40 (Qm/Q); 70 (Sm/S), 50 (Sm/S), 40 (Sm/S); and 70 (Sm/Qm), 50 (Sm/Qm), 40 (Sm/Qm). Detailed views on 2D-DIGE gel sections with protein spots revealing differential relative abundance patterns in the set of 12 experiment variants.

Proteome analysis of the crown tissues in 12 experiment variants focused on 24 biologically relevant ratios, and the given criteria (min. 1.5-fold change at the 0.05 level determined by Student’s *t*-test) led to an identification of 113 protein spots revealing differential relative abundance (DAPs) out of which 76 protein spots matched the criteria given for protein identification (**Figure 1**). The representative 2D-DIGE gel image details of the selected protein spots in the 12 experiment variants are given in **Supplementary Figure S2**.

**Figure 2:**
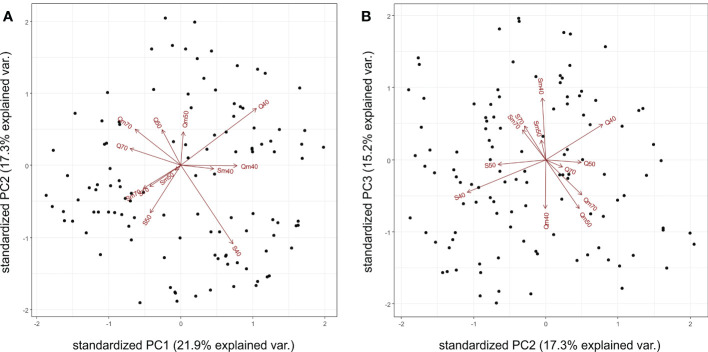
Principal component analysis of the 113 differentially abundant proteins (DAPs) with indicated positions of the 12 experiment variants showing the first two principal components PC1 versus PC2 **(A)** and PC1 versus PC3 **(B)**. Q means Quintus, S means Septima, m means *Metopolophium dirhodum* treatment, 40 means 40% SWC, 50 means 50% SWC, and 70 means 70% SWC.

The PCA results revealed that the first four PC axes explained 66.7% of the total variation in the experimental data. The first two PC variables, PC1 (21.9%) and PC2 (17.3%), differentiated the 12 experiment variants according to their water regimes with 70% SWC in the left part and 40% SWC in the right part of the PCA plot, respectively, while 50% SWC treatments are located between the 70% and 40% SWC. The other differentiation is according to the genotype: Quintus variants are located in the upper part of the PCA plot, while Septima variants are located in the lower part of the plot (**Figure 2A**). In PC1 versus PC3 (15.3%) projection, the aphid-treated variants of Septima (Sm40, Sm50, and Sm70) clustered together and were placed opposite to the cluster of the aphid-treated variants of Quintus (Qm40, Qm50, and Qm70; **Figure 2B**). Similarly, cluster analysis of the 12 experiment variants revealed the dominant effect of the water regime treatments followed by the effect of genotypes and the effect of aphid treatments on the proteome profile in the sample sets. The two major clusters clearly distinguished the 70% and 40% SWC treatments, while the samples of 50% SWC treatments were present in both clusters; similarly, both cultivars and both the aphid-treated and untreated variants were also present in both clusters (**Supplementary Figure S3**). These results thus indicate the dominant impacts of the contrasting SWC treatments, namely, 70% and 40% SWC, on protein relative abundance in the 113 DAPs. Subclusters 1a and 1b in cluster 1 and subclusters 2a, 2b, and 2c in cluster 2 clearly distinguished the Septima and Quintus genotypes.

**Figure 3:**
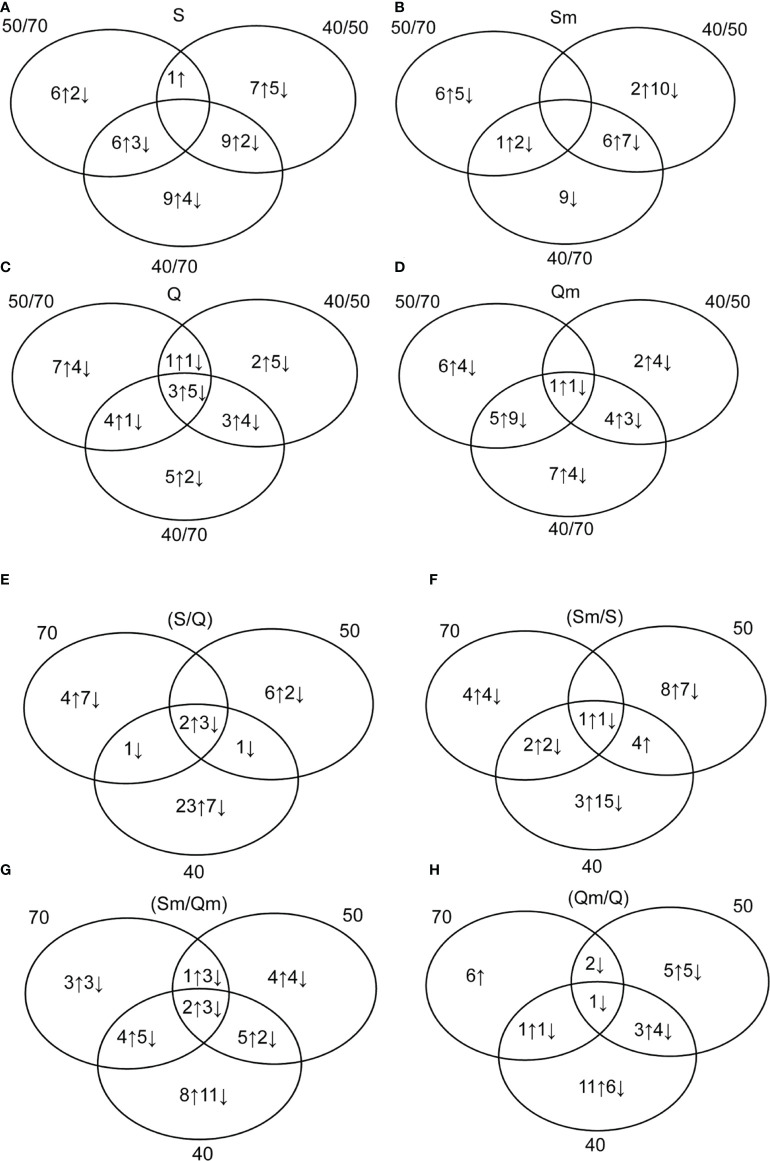
Venn diagrams showing the numbers of upregulated (↑) and downregulated (↓) protein spots in 24 biologically relevant ratios: in Septima **(A)** [S (50/70), S (40/70), S (40/50)]; in Quintus **(B)** [Q (50/70), Q (40/70), Q (40/50)]; in the aphid-infested Septima **(C)** [Sm (50/70), Sm (40/50), Sm (40/70)]; in the aphid-infested Quintus **(D)** [Qm (50/70), Qm (40/50), Qm (40/70)]; in the non-infested Septima versus Quintus (S/Q) **(E)** [70 (S/Q), 50 (S/Q), 40 (S/Q)]; in the aphid-infested versus non-infested Septima (Sm/S) **(F)** [70 (Sm/S), 50 (Sm/S), 40 (Sm/S)]; in the aphid-infested Septima versus Quintus (Sm/Qm) **(G)** [70 (Sm/Qm), 50 (Sm/Qm), 40 (Sm/Qm)]; and in the aphid-infested versus non-infested Quintus (Qm/Q) **(H)** [70 (Qm/Q), 50 (Qm/Q), 40 (Qm/Q)].

The Venn diagrams of the 24 biologically relevant ratios grouped into eight diagrams showing the numbers of DAPs including both identified and non-identified protein spots, i.e., a total of 113 DAPs, are given in **Figure 3**. Regarding the differences between the three water regime treatments, i.e., (40/70), (50/70), and (40/50) ratios, the cultivar Septima revealed a total of 38 protein spots with an increased relative abundance and 16 protein spots with a decreased relative abundance between the three water regime treatments (**Figure 3A**), while in Quintus, 25 protein spots revealed a significant increase and 22 protein spots revealed a significant decrease, respectively, between the three water regimes (**Figure 3B**). In the aphid-treated Septima (Sm) plants, 15 protein spots revealed an increase and 33 protein spots revealed a significant decrease between the three water regime treatments (**Figure 3C**), while in the aphid-treated Quintus (Qm), the analogous treatments led 25 protein spots to increase and 25 protein spots to decrease, respectively (**Figure 3D**). Regarding the ratio between Septima and Quintus (S/Q) plants, a total of 38 protein spots revealed an increase, while 21 protein spots revealed a decrease across the three water regime treatments (**Figure 3E**). Meanwhile, between the aphid-treated Septima and Quintus (Sm/Qm), 27 increased spots and 31 decreased spots were found in the set of the three water regime treatments (**Figure 3G**). Regarding the ratio between the aphid-treated and untreated plants, a total of 22 increased spots and 29 decreased spots were found in Septima (Sm/S; **Figure 3F**), while a total of 26 increased spots and 19 decreased spots were found in Quintus (Qm/Q; **Figure 3H**), respectively. Information on the significant differences in the 24 studied ratios between experiment variants for the identified protein spots is also provided in **Supplementary Table S3**.

**Figure 4:**
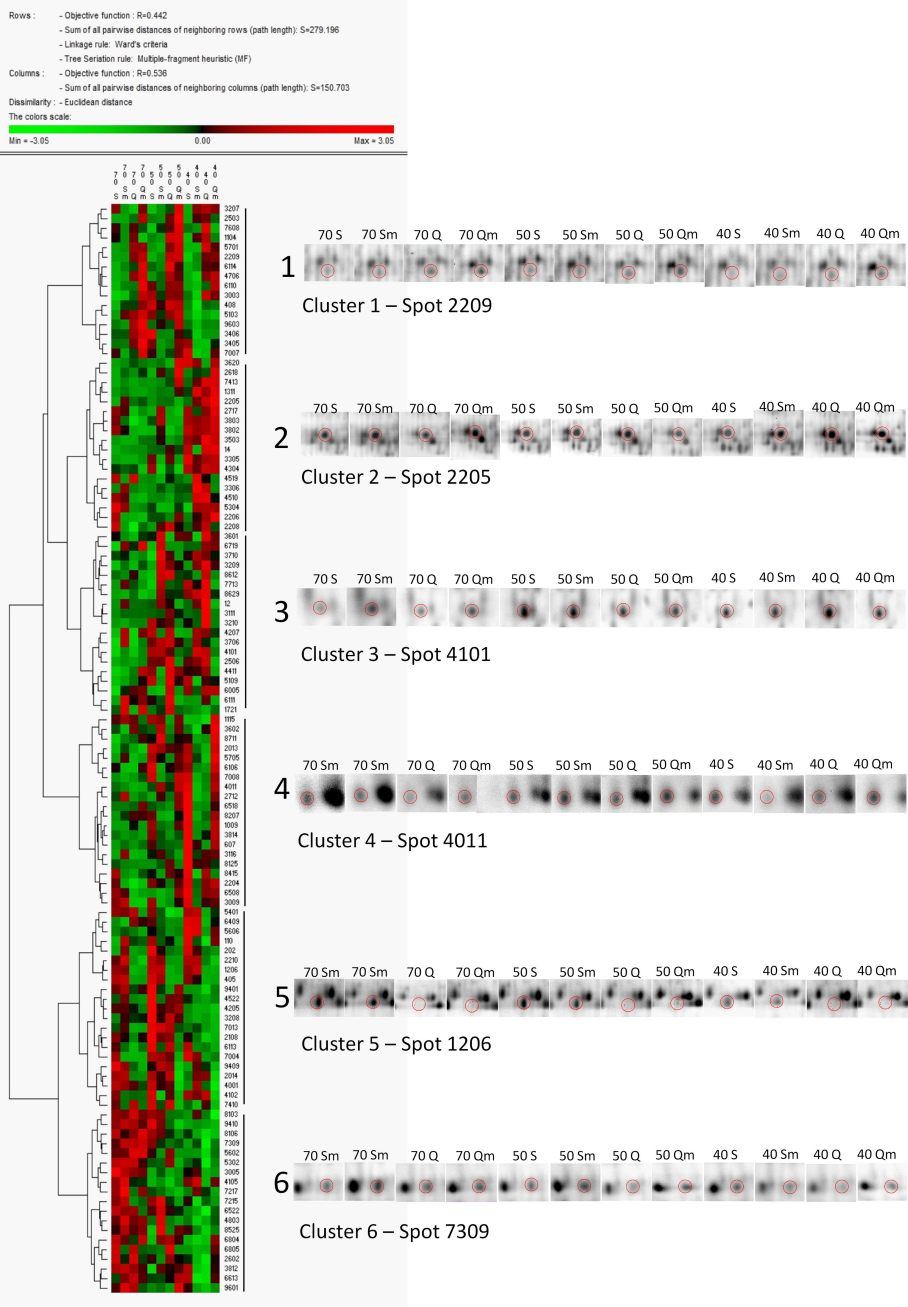
Cluster analysis of the 113 DAPs in the set of 12 experiment variants (S70, S50, S40, Sm70, Sm50, Sm40, Q70, Q50, Q40, Qm70, Qm50, Qm40). The data were normalized using *Z*-score transformation, and cluster analysis was performed in PermutMatrix software using Euclidean distances and Ward’s minimum criteria as algorithms for clusterogram construction. Details on the 2D-DIGE gel images of selected protein spots representing the individual clusters are given.

Cluster analysis of the 113 DAPs revealed six major clusters, i.e., six major patterns of protein relative abundance in the set of 12 experiment variants (**Figure 4**). Protein spots in cluster 1 revealed lower relative abundance in Septima than in Quintus, and protein spots in cluster 2 revealed enhanced relative abundance in severe water deficit of 40% SWC with respect to mild water deficit of 50% SWC and the control variants. Clusters 5 and 6 reveal the opposite pattern, i.e., protein spots in cluster 5 revealed enhanced relative abundance in Septima with respect to Quintus, and protein spots in cluster 6 revealed enhanced relative abundance in the control with respect to the variants exposed to water deficit treatments.

**Figure 5:**
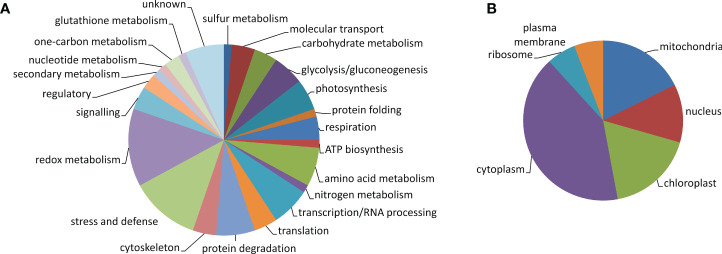
Pie chart of the identified protein spots showing protein classification according to GO biological processes **(A)** and cellular localization **(B)**.

**Figure 6:**
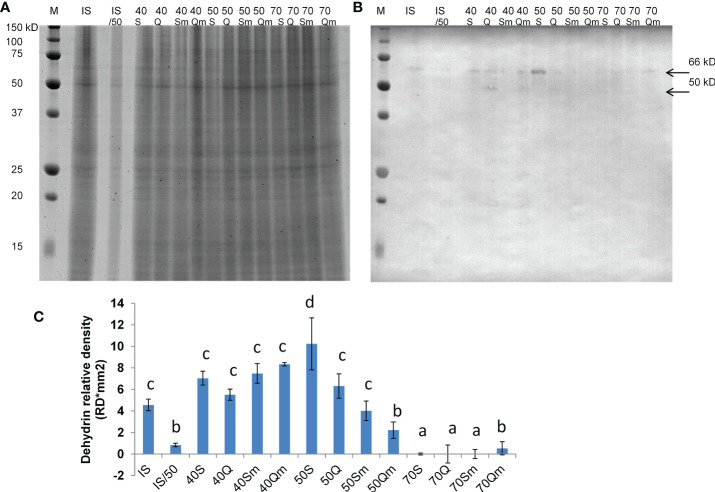
Representative 1-D SDS-PAGE gel stained by CBB R-250 **(A)**, representative immunoblot with detected dehydrin proteins **(B)**, and the results of densitometric analysis of dehydrin proteins detected in the immunoblot **(C)**. In **(B)**, the position of two detected dehydrin bands of 66 and 50 kDa corresponding to WCS66 and WCS120 proteins, respectively, is indicated by an arrow. In **(C)**, the data columns represent mean values and the error bars represent standard deviation from three replicates (*n* = 3), and different letters above the data columns indicate significant differences in the density of dehydrin bands determined by ANOVA, LSD 0.05 test (STATISTICA version 14, Inc., TIBCO, UK). M means molecular marker (Bio-Rad), IS means internal standard as an equimolar mixture of all samples loaded on the gel, IS50 means internal standard at half dilution (50%), Q means Quintus, S means Septima, m means treatment with *Metopolophium dirhodum*, 70 means 70% SWC, 50 means 50% SWC, and 40 means 40% SWC.

The 76 identified proteins were grouped into 23 kinds of biological processes according to GO annotation: amino acid metabolism (5 proteins), ATP biosynthesis (1), carbohydrate metabolism (3), cytoskeleton (3), glutathione metabolism (1), glycolysis/gluconeogenesis (4), molecular transport (3), nitrogen metabolism (1), nucleotide metabolism (1), one-carbon metabolism (2), photosynthesis (4), protein degradation (5), protein folding (1), redox metabolism (10), regulatory proteins (2), respiration (3), secondary metabolism (1), signaling (3), stress and defense (9), sulfur metabolism (1), transcription/RNA processing (5), translation/protein biosynthesis (3), and proteins with unknown function (5) (**Figure 5A**). Regarding protein cellular localization, 21 proteins were localized to the cytoplasm, 6 proteins were localized to the nucleus, 9 proteins were localized to the mitochondria, 9 proteins were localized to the chloroplasts, 3 proteins were localized to the ribosomes, and 3 proteins were localized to the plasma membrane (**Figure 5B**). The list of the identified proteins organized according to their assignments to biological processes is provided in **Supplementary Table S3**. The proteins other than the uncharacterized ones which were predicted to interact with the identified proteins and the protein interacting networks drawn by STRING are given in **Supplementary Figure S4**. Since one of the identified proteins LEA-19 (QPO15911.1) was predicted to interact with acidic dehydrin WCOR410, 1-D immunoblot analysis of dehydrin proteins in the samples was performed to validate the 2D-DIGE data. The results of the immunoblot analysis and the representative 1-D SDS-PAGE gel stained by CBB R-250 showing protein load and representative immunoblot are given in **Figure 6**. Two bands of dehydrin proteins with the apparent molecular weight of 66 and 50 kDa corresponding to the positions of WCS66 and WCS120 proteins, respectively, identified in cold-treated wheat (Sarhan et al., 1997; Vítámvás et al., 2019) were detected. Densitometric analysis of the immunoblot revealed the highest dehydrin accumulation in the S50 sample followed by the samples grown at 40% SWC and the remaining three samples grown at 50% SWC and Qm70, while practically no dehydrin proteins were detected in the remaining three 70% SWC treatments (**Figures 6B, C**). In the following text, we will focus on the three kinds of stress treatments as defined in the aims:

1. DAPs revealing significant differences between the three water regimes
2. DAPs revealing significant differences between the aphid-infested plants as compared with non-infested ones
3. DAPs revealing significant differences between the combined stress treatments of water deficit and aphid infestation (Sm40, Sm50, Qm40, Qm50) as compared with the single stress treatments of water deficit (S40, S50, Q40, Q50) and aphid infestation (Sm70, Qm70).

## 4 Discussion

### 4.1 Spring wheat responses to water deficit, aphid rearing, and the combined water deficit and aphid treatments

Regarding our previous study on wheat morphophysiological characteristics and aphid life tables in two spring wheat cultivars exposed to the combined treatment of water deficit and aphid infestation (Saska et al., 2022), the following three major effects of stress treatments were studied at the proteome level of wheat: effects of drought treatments (40%, 50% SWC), effect of aphid infestation (variants indicated by “m”), and combined drought and aphid treatments (variants Qm40, Qm50, Sm40, Sm50). The key proteins responding to the three stress treatments studied in Quintus and Septima are summarized in **Table 1**.

**Table 1:** An overview of the key proteins responding to water deficit, aphid infestation, and the combined water deficit and aphid treatment with respect to the control in the spring wheat cultivars Quintus and Septima.

Stress treatment	Differentially abundant proteins (DAPs)	
	Quintus	Septima
Water deficit (40%, 50% SWC)	NAD(P)H quinone oxidoreductase FQR1 (ssp 2108) ↓	NAD(P)H quinone oxidoreductase FQR1 (ssp 2108) ↑, mitochondrial ETC flavoprotein α (ssp 5103), citrate synthase 4 (ssp 2506), malate dehydrogenase (ssp 5103), chloroplast Cu/Zn-SOD (ssp 8125) ↑, mitochondrial porin 3 (ssp 110) ↑
Aphid infestation (m)	UTP-glucose-1-phosphate uridylyltransferase (ssp 8525), UDP-glucose 6-dehydrogenase 4 (ssp 5401) ↑, ATP synthase α (ssp 3306) ↓, GST 1-like (ssp 3207) ↑	UTP-glucose-1-phosphate uridylyltransferase (ssp 8525), UDP-glucose 6-dehydrogenase 4 (ssp 5401) ↑, MnSOD (ssp 3111), Hsc70-1, Hsc70-2 (ssp 4519, 8711, 9601), actin depolymerizing factor 4 (ADF4; ssp 6110, 6111), UEV1D (ssp 3009) ↑
Combined water deficit and aphid treatment (Qm40, Qm50, Sm40, Sm50)	IN2-1-like protein B (ssp 7413; protein glutathionylation), isoflavone reductase (ssp 6106) ↑	Probable glutathione-S-transferase DHAR1 (ascorbate dehydrogenase; ssp 4101) ↑, RubisCO LSU (ssp 2618, 3405), RubisCO SSU (ssp 4011) ↓

↑ indicates an enhanced protein relative abundance with respect to the control, while ↓ indicates a decreased protein relative abundance with respect to the control.

#### 4.1.1 Effects of water deficit

Based on the evaluation of morphophysiological characteristics, Septima revealed an enhanced water deficit tolerance with respect to Quintus which is demonstrated by larger shoot biomass, lower WSD, and higher ψπ and ψπ100 values (Saska et al., 2022). PCA (**Figure 2**) and cluster analysis (**Supplementary Figure S3**) of all experiment variants clearly distinguished the 70% and 40% SWC treatments; considering the 50% SWC treatments, Septima varieties (S50, Sm50) clustered together with the 70% SWC varieties, while the Quintus varieties (Q50, Qm50) clustered together with the 40% SWC varieties indicating that the 50% SWC treatment represented stress for Quintus, but not for Septima.

2D-DIGE coupled with MALDI-TOF/TOF led to an identification of 76 DAPs covering a broad range of biological processes from signaling *via* stress acclimation to long-term adaptation processes. Signaling proteins include one Ser/Thr protein kinase 38-like (ssp 4304) and two β subunits of heterotrimeric G proteins (ssp 5103, 6409). G protein signaling is involved in plant immunity against a wide range of pathogens including bacteria such as *Pseudomonas syringae* and necrotrophic fungi such as *Fusarium oxysporum* as evidenced by a number of G protein subunit mutants with impaired resistance (reviewed in Zhang et al., 2012; Zhong et al., 2019). However, in our study, both G protein subunit β identified revealed a decreased relative abundance in the aphid-treated Septima with respect to the non-treated plants under both optimum and water deficit treatments. This result probably deals with the timing of samplings which was relatively late after the beginning of stress treatments with both drought and aphids. Proteomics analysis also revealed enhanced protein turnover as indicated by the enhanced relative abundances of ribosomal proteins S21 (ssp 2014) and S2 (ssp 9409) and eIF3 subunit i (ssp 1206) as well as proteins involved in proteasomal protein degradation as evidenced by proteasome subunit α type-6 (ssp 2210, 8106) and 26S protease regulatory subunit 6B (ssp 5304) similarly to Kosová et al. (2017). Alterations in the regulatory subunit of 26S proteasome represent evidence on the involvement of further mechanisms such as ABA signaling and SUMOylation which is competitive to ubiquitination to regulate protein stability under drought (Antoni et al., 2011; Stone, 2014; Marshall and Vierstra, 2019). The precise regulation of protein SUMOylation and protein turnover was found to underlie a superior drought tolerance in the wheat mutant RYNO3926 (Le Roux et al., 2020). In contrast, spot 3009 identified as ubiquitin-conjugating enzyme E2 variant 1D-like (UEV1D) forms a heterodimer with UBC13 and catalyzes the synthesis of non-canonical poly-ubiquitin chains that are linked through lysine 63 (K-63). It is important to note that this type of polyubiquitination does not lead to proteasomal protein degradation. Instead, the UEV1D–UBC13 complex is involved in DNA postreplication repair underlying DNA damage tolerance (DDT) since the UEV1D–UBC13 complex catalyzes Lys-63-linked polyubiquitination of the proliferating cell nuclear antigen (PCNA) and activates the transcription of target genes (Wen et al., 2008).

Proteomics analysis has complemented the morphophysiological data, and it has revealed that Septima has better drought tolerance than Quintus due to its ability to maintain key processes related to mitochondrial metabolism and respiration, namely, tricarboxylic acid cycle (TCA cycle) located in the mitochondrial matrix and respiratory electron transport chain (ETC) located in the mitochondrial inner membrane. Maintenance of a high turnover of energy metabolism is crucial for plant stress acclimation since the synthesis of novel compounds requires energy (Kosová et al., 2018). TCA enzymes include mitochondrial citrate synthase 4 (ssp 2506) and malate dehydrogenase (ssp 5103), while components of the mitochondrial respiratory ETC include NAD(P)H quinone-dependent dehydrogenase FQR1 (ssp 2108) and mitochondrial electron transfer flavoprotein subunit α (ssp 5103). Spot 2108 belongs to cluster 5, i.e., revealing an enhanced relative abundance in Septima with respect to Quintus. In addition, relatively enhanced mitochondrial metabolism in drought-treated Septima as compared to Quintus is indirectly indicated by the enhanced relative abundance of ssp 101 identified as mitochondrial outer membrane porin 3 involved in molecular transport between the mitochondria and cytoplasm. Enhanced levels of proteins involved in mitochondrial respiration as well as proteins involved in mitochondrial transport indicate a key role of mitochondrial respiration in drought acclimation in wheat. Moreover, Septima also revealed an enhanced level of chloroplast Cu/Zn-SOD (ssp 8125), indicating better regulation of redox stress in the key organelle for energy metabolism which is crucial for drought tolerance (on the role of redox regulation in drought stress see, e.g., Hajheidari et al., 2007; Pilon et al., 2011). Moreover, Septima with respect to Quintus at the control (70% SWC) as well as severe drought (40% SWC) revealed an enhanced relative abundance in spot 3803 identified as lipoxygenase-2 and ssp 3503 identified as aldehyde dehydrogenase 7b which are involved in the detoxification of lipid peroxides that arise as products of hydrogen peroxide reaction with unsaturated fatty acids. In the study of Zogli et al. (2020), four lipoxygenases were identified in the response of switchgrass (*Panicum virgatum*) to greenbug infestation.

In addition, the water deficit-treated Quintus (40 Q, 50 Q) revealed a significant increase in germin D (ssp 3111) with respect to the control plants (70 Q). Germins and germin-like proteins belong to the PR-15 and PR-16 protein families, respectively, and are hydrophilic glycoproteins located in the extracellular matrix (ECM) which possess a beta-barrel structure characteristic of cupin proteins. Some germins and GLPs possess enzymatic activities, namely, oxalate oxidase (OXO) and superoxide dismutase (SOD) activities (Bernier and Berna, 2001). Oxalate oxidases catalyze the cleavage of oxalate to produce CO_2_, and it is known that oxalate oxidase can represent an alternative source of CO_2_ in drought-stressed C3 plants with reduced stomatal conductance (Prasad and Shivay, 2017).

Moreover, drought led to an enhanced turnover in sulfur metabolism as indicated by the cystathionine β-synthase (CBS) domain protein CBSX3 identified in ssp 12. It revealed an increase at 50% SWC with respect to the control 70% SWC in both non-infested Septima and Quintus plants and a decrease in infested Quintus plants at both water deficit treatments, i.e., 40% and 50% SWC. Cystathionine β-synthase domain-containing proteins are mitochondrial proteins that catalyze the first step in the transsulfuration pathway lying in sulfur transfer between serine and homocysteine to form cystathionine. Three CBS protein isoforms named CBSX1–3 were identified in *Arabidopsis thaliana*. CBSX1 and CBSX2 proteins are localized in the chloroplast and reported to regulate the ferredoxin–thioredoxin system, while CBSX3 is localized in the mitochondria and reported to regulate the mitochondrial NADP–thioredoxin (NADP-Trx, NTS) system, thus playing an important role in maintaining redox homeostasis (Yoo et al., 2011). Enhanced CBSX3 protein was found in the salt-treated barley *Hordeum vulgare* cv. Tadmor and wild *H. marinum* in the study of Maršálová et al. (2016). In contrast, two spots ssp 5302 and 6522 identified as S-adenosylmethionine synthetase (SAMS) revealed a decreased relative abundance in water deficit-treated plants with respect to the control ones (70% SWC) which is interesting since stress treatments usually induced an enhanced need for SAM as a universal methylating agent in plant cells. Plant stress proteomics papers dealing with SAMS were reviewed by Kosová et al. (2018).

These findings correspond with enhanced tolerance to water deficit (dehydration) found in Septima with respect to Quintus determined as Ψπ100 (osmotic potential recalculated per 100% water saturation). Septima revealed higher Ψπ and Ψπ100 values than Quintus across the different experiment variants. In accordance, Quintus revealed higher WSD values than Septima across different experiment variants (Saska et al., 2022). Similar patterns were also observed for growth-related characteristics, i.e., total aboveground biomass and leaf length which clustered together with Ψπ and Ψπ100 values. The morphophysiological characteristics of total aboveground biomass, leaf length, Ψπ, and Ψπ100 clustered together with ssp 9601 identified as an Hsc70 2-like protein which is an important stress-inducible chaperone (**Supplementary Figure S6**).

#### 4.1.2 Effects of aphid inoculation

The absence of aphid-specific resistance proteins is in accordance with the plant vigor hypothesis formulated in our previous study (Saska et al., 2022). Although no specific resistance proteins related to wheat response to aphid infestation were found by proteomic analyses, several proteins related to plant immunity and plant–pathogen interactions were identified. These include, for example, Hsc70-1 and Hsc70-2 isoforms involved in interaction with SGT1 and *R*-gene-mediated resistance, two ricin B-like lectins R40C1 and R40G3, ubiquitin-conjugating enzyme E2 variant 1D-like (UEV1D) involved in non-proteasomal protein ubiquitination and DNA damage repair, or actin depolymerizing factor 4 (ADF4) involved in actin filament reassembly and recognition of bacterial and fungal microbe-associated molecular patterns (MAMPs).

Protein ssp 4519 was identified as an Hsc70-1 isoform, while ssp 8711 and 9601 were identified as Hsc70 2-like isoforms. The Hsc70 proteins function as chaperones and can be involved in *R*-gene-mediated resistance mechanisms to pathogens *via* their interaction with the SGS domain in the SGT1 protein (Noël et al., 2007). Four isoforms were described for the Hsc70 protein in *A. thaliana*, of which Hsc70-1 and Hsc70-3 isoforms are constitutively expressed, while Hsc70-2 and Hsc70-4 isoforms are pathogen-inducible (Noël et al., 2007). Enhanced levels of two Hsc70-2 isoforms were found in the *Fusarium*-treated wheat cultivars Sumai 3 and SW Kadrilj by Kosová et al. (2021a). Two protein spots 2206 and 4411 were identified as ricin B-like lectin proteins R40C1 and R40G3, respectively. Lectins are glycoproteins involved in saccharide signaling whose level can affect plant development. Ricin B lectins were reported in plant response to pathogens and were found to be involved in caspase 3-like protease-induced apoptosis (reviewed in Lannoo and Van Damme, 2014). Ricin B lectin 2 proteins were found to increase in the cold-treated winter wheat Samanta (Kosová et al., 2013) and winter barley Luxor (Hlaváčková et al., 2013) crowns. Moreover, two protein spots identified as ricin B lectin R40G2 protein were found enhanced in the *Fusarium culmorum*-treated susceptible wheat cultivar SW Kadrilj (Kosová et al., 2021a). A chimeric lectin with two agglutinin domains and one ETX/MTX2 toxin domain was mapped to the *Fhb1* locus on the 3BS chromosome in the *Fusarium*-resistant spring wheat Sumai 3 (Rawat et al., 2016). A chimeric protein SNA-I isolated from elderberry (*Sambucus nigra*) belonging to type-2 ribosome-inactivating protein (RIP) and containing chain A with RNA N-glycosidase activity and chain B with lectin activity reduced adult survival and fecundity of the pea aphid (*Acyrtosiphon pisum*) and tobacco aphid (*Myzus nicotianae*) (Shahidi-Noghabi et al., 2008). Transgenic experiments indicated that the SNA-I chain B saccharide-binding activity underlies its insecticidal activity. Spot 4411 identified as ricin B-like lectin protein R40G3 was found to increase in the water deficit-treated Septima with respect to the control plants as well as in the aphid-treated Quintus with respect to Septima plants grown at control watering (70% SWC), indicating its enhanced relative abundance under water deficit stress as well as enhanced abundance in the relatively more susceptible Quintus with respect to the tolerant Septima plants. Spots 6110 and 6111 identified as actin-depolymerizing factor 4-like isoform X1 (ADF4) were reported to form the complex with cofilin and to regulate the remodeling of actin filaments during innate immune signaling in plant cells as a response to bacterial and fungal MAMPs sensed by pattern recognition receptors (PRRs). ADF4 is thus involved in actin cytoskeleton rearrangement in response to bacterial MAMPs (Henty-Ridilla et al., 2014).

Our data did not indicate activation of any specific resistance mechanisms such as pore-forming toxins, etc. in response to aphid infestation which corresponds with the finding of Ferry et al. (2011) on wheat cv. Claire and their conclusion that an absence of specific aphid response mechanisms corresponds with the observed lack of aphid resistance in commercial wheat cultivars.

However, we have found some wheat responses to aphids shared by both cultivars studied. Both Quintus and Septima activated the biosynthesis of oligo- and polysaccharides involved in cell wall remodeling as indicated by the enhanced relative abundance of UTP-glucose-1-phosphate uridylyltransferase which catalyzes the synthesis of activated UDP-glucose. UDP-glucose is necessary for glucose incorporation into oligo- and polysaccharides. Increased levels of the enzyme involved in UDP-glucose biosynthesis in both cultivars under aphid infestation may indicate a plant strategy aimed at reducing the availability of free glucose in cells, thus reducing the availability of glucose for aphids, or enhanced UDP-glucose may indicate increased biosynthesis of cell wall polysaccharides and enhanced cell wall remodeling in response to aphid attack. In accordance with this hypothesis, increased levels of UDP-glucose 6-dehydrogenase 4 were found in the water deficit-treated and aphid-infested variants. This enzyme is involved in the biosynthesis of UDP-glucuronic acid which is an important component of cell wall polysaccharides; thus, the enhanced abundance of the enzyme indicates enhanced cell wall remodeling in response to water deficit and aphid infestation. Enhanced levels of the enzymes involved in cell wall remodeling in response to stresses such as drought and salinity were found by Mostek et al. (2015); Vítámvás et al. (2015), and others.

Aphid infestation led to an increased level of oxidative stress including both ascorbate–glutathione cycle-related enzymes and peroxiredoxins as ascorbate–glutathione cycle-independent redox systems (Dietz et al., 2006). Plants try to alleviate redox stress due to an increase in ROS-scavenging enzymes in the chloroplasts and mitochondria as organelles involved in aerobic metabolism (an increase in mitochondrial MnSOD and chloroplast Cu/Zn-SOD). Our data have shown that aphid feeding induced higher oxidative stress in Quintus than in Septima as indicated by decreased mitochondrial ATP synthase α subunit (ssp 3306) involved in ATP biosynthesis in Quintus, while mitochondrial MnSOD isoform (ssp 3111) was significantly more increased in the water deficit-treated Quintus when compared with Septima. The aphid-infested Septima revealed enhanced mitochondrial proteins MnSOD and ATP synthase α with respect to the aphid-infested Quintus indicating that Septima revealed enhanced resistance to aphids with respect to Quintus. Spot 7013 was identified as a peroxiredoxin 2C-like protein belonging to the PRX5 subfamily. PRX5 family members are homodimeric thioredoxin (TRX) peroxidases found in the cytoplasm, peroxisomes, and mitochondria (Bhatt and Tripathi, 2011).

Plants try to alleviate oxidative stress by the downregulation of aerobic metabolism, namely, photosynthetic and respiratory electron transport processes (Apel and Hirt, 2004). Decreased respiratory electron transport chain is also reflected by decreased ATP biosynthesis as indicated by decreased ATP synthase α subunit relative abundance in Quintus. Alterations in response to aphid treatments were also found in nitrogen assimilation, namely, glutamine synthetase (GS; EC 6.3.1.2.) GS2C chloroplastic isoform (Wang et al., 2015), where a significant decrease in GS2C in the aphid-treated Septima plants with respect to non-treated ones grown at 40% SWC was found which indicates reduced nitrogen assimilation in aphid-infected plants subjected to water deficit as a potential strategy to reduce nitrogen availability to aphids.

Enhanced sensitivity of Quintus to aphid infestation is also manifested by the presence of a dehydrin protein in the Qm70 variant and the absence of dehydrin in the Sm70 variant in the immunoblot (**Figure 6**). Similarly, Quintus in comparison with Septima also revealed higher levels of GST 1-like (ssp 3207) involved in xenobiotics detoxification *via* GST conjugation and higher levels of isoflavone reductase which is involved in isoflavonoid biosynthesis. It is evident that GST as an important xenobiotic detoxification enzyme plays an important role in a broad range of stresses including both water deficit and aphid treatments. As an example, enhanced GST levels were found in the drought-treated wheat mutant line BIG8-1 (Le Roux et al., 2021). Enhanced GST levels were also reported by Ferry et al. (2011) in wheat infested by the aphid *S. avenae*. Flavonoids and isoflavonoids are secondary metabolites which help the plants to alleviate secondary redox stress caused by drought, and increased levels of these metabolites were found in drought-treated wheat under both continuous drought and pulsed drought treatments (Stallmann et al., 2020).

#### 4.1.3 Combined effects of drought and aphid inoculation

The combined drought and aphid treatments represent a stronger stress for Quintus compared with Septima as indicated by the enhanced relative abundance of IN2-1-like protein B involved in protein glutathionylation and of isoflavone reductase involved in phytoalexin biosynthesis and ROS regulation in drought- and aphid-treated Quintus (Qm50, Qm40) as compared with the corresponding Septima samples since all these processes are aimed at stress alleviation. IN2-1-like protein B was reported to reveal a glutathione-S-transferase activity and is proposed to be involved in target protein glutathionylation which was reported to protect the target thiol groups in cysteine residues against oxidative modifications (Diaz-Vivancos et al., 2015; Martínez-Seidel et al., 2021; reviewed in Kosová et al., 2021b). Isoflavone reductase homolog IRL (ssp 6106) has an NADPH-dependent oxidoreductase and an isoflavone reductase activity and is involved in the biosynthesis of isoflavonoid phytoalexins in plants. Phytoalexins are known to play an important role in plant defense against fungal pathogens. Spot 6106 revealed relatively enhanced relative abundance in the drought- and aphid-treated Quintus (Qm40) with respect to Septima (Sm40). Kim et al. (2003) reported an induction of the rice isoflavone reductase-like gene *OsIRL* by the rice blast fungus (*Magnaporthe grisea*) elicitor as well as a positive effect of jasmonic acid (JA) and negative effects of abscisic acid (ABA) and salicylic acid (SA) on *OsIRL* induction. Cheng et al. (2015) reported an enhanced resistance of transgenic soybean overexpressing *GmIRF* isoflavone reductase to *Phytophthora sojae* due to enhanced accumulation of the soybean-specific phytoalexins (glyceollins) and reduced ROS levels.

However, Septima also revealed an enhanced level of ascorbate dehydrogenase DHAR1 (ssp 4101) involved in ROS detoxification *via* the ascorbate–glutathione cycle in the combined drought- and aphid-treated variants (Sm50, Sm40) with respect to the control ones indicating enhanced redox stress. Moreover, significantly decreased levels of RubisCO subunits (ssp 2618, 3405, 4011) were found in the combined drought and aphid treatments (see **Supplementary Table S3** data) in comparison to single stress treatments, indicating the enhanced intensity of the combined stress.

## 5 Conclusion

Proteomic analysis revealed a dominant effect of water regime treatments followed by the effect of genotypes. while the effect of aphid treatments was the lowest. In accordance with our previous results, Septima revealed enhanced water deficit tolerance with respect to Quintus which is indicated by enhanced levels of proteins involved in mitochondrial respiration as a key process of energy metabolism necessary for stress acclimation. Aphid treatments led to induction of some proteins involved in *R*-gene-mediated resistance, and ricin B-like lectins and MAMP recognition were identified in aphid-treated plants. The combined water deficit and aphid treatments led to the induction of proteins involved in detoxification processes such as protein glutathionylation and phytoalexin biosynthesis and reduced levels of RubisCO subunits which indicates an enhanced stress effect of the combined treatments on wheat plants. Enhanced levels of proteins involved in proteasomal degradation indicate enhanced protein degradation; in our future studies, the detection of the target proteins with specific antibodies against ubiquitin or SUMO peptides will provide important information on the kinds of proteins revealing enhanced protein flux upon the single and combined stress treatments.

## Data availability statement

The datasets presented in this study can be found in online repositories. The names of the repository/repositories and accession number(s) can be found below: https://www.ebi.ac.uk/pride/archive/, PXD035083.

## Author contributions

Conceptualization was done by PS, JS, and PV. Data collection was done by JS, KK, and PV. 2D-DIGE gel analysis and protein spot excision were performed by PV and JV. Protein spot identification by MALDI-TOF/TOF was performed by JR and SP and data loading into the ProteomeXCHange repository was performed by SP. Formal analysis was carried out by KK. Writing—original draft preparation was done by KK. Writing—review and editing was performed by all authors. Funding acquisition was done by PS. All authors contributed to the article and approved the submitted version.

## Funding

This work was supported by the Czech Science Foundation (CSF) project 18-13174J and by the institutional project from the Czech Ministry of Agriculture (MZe CR) RO0418. The work was also supported by the Luxembourg National Research Fund (FNR) grant SMARTWALL n. C15/SR/10240550.

## Conflict of interest

The authors declare that the research was conducted in the absence of any commercial or financial relationships that could be construed as a potential conflict of interest.

## Publisher’s note

All claims expressed in this article are solely those of the authors and do not necessarily represent those of their affiliated organizations, or those of the publisher, the editors and the reviewers. Any product that may be evaluated in this article, or claim that may be made by its manufacturer, is not guaranteed or endorsed by the publisher.

## Supplementary material

Supplementary Figure 1 Representative image of plants used for the experiments. Q means Quintus, S means Septima, m means treatment with *M. dirhodum*, 70 means 70% SWC, 50 means 50% SWC, 40 means 40% SWC, R means replicate.

Supplementary Figure 2 Representative images of 2D-DIGE gels of the 12 experiment variants.

Supplementary Figure 3 Cluster analysis of 113 differentially abundant proteins (DAPs) showing the clustering of 12 experiment variants. Abbreviations: 40 – 40% soil water content (SWC), 50 – 50% SWC, 70 – 70% SWC, m – *M. dirhodum* infestation, Q – Quintus, S – Septima.

Supplementary Figure 4 Protein-protein interactions networks drawn by STRING online tool (https://www.string-db.org) for the identified proteins.

Supplementary Figure 5 Cluster analysis of the 113 DAPs and six morphophysiological characteristics (fresh above-ground biomass, leaf length, WSD, Ψπ, Ψπ100, Fv/Fm). The data were normalized using Z-score transformation and cluster analysis was performed in PermutMatrix software using Euclidean distances and Ward´s minimum criteria as algorithms for clusterogram construction.

Supplementary Table 1 Estimated mean values for leaf morpho-physiological traits of spring wheat seedlings under the variable conditions of water supply (soil water capacity, SWC [%]) and aphid infestation. SE denotes standard error. The same letters within the columns indicate values that were not significantly different from each other based on a paired bootstrap test. Data from Saska et al. (2022).

Supplementary Table 2 Life table parameters (means ± SE) of *Metopolophium dirhodum* reared on two cultivars (Quintus and Septima) of *Triticum aestivum*. SE were estimated with bootstrapping (100,000 resamplings). *λ* - finite rate of increase, *r* - intrinsic rate of increase, *R*_0_ – net reproductive rate; T – generation time. The same letters within the columns indicate values that were not significantly different from each other based on a paired bootstrap test. Data from Saska et al. (2022).

Supplementary Table 3 A list of the identified protein spots revealing differential abundance, i.e., min. 1.5 fold change at 0.05 level determined by Student’s *t*-test, in at least one out of 24 biologically relevant sample ratios. SSP – protein spot number; accession n. according to NCBI protein database (access: 23rd April, 2021; taxonomy: Triticeae: NCBI txid147389_20210423 (518563 sequences; 220219273 residues)), MS score – MASCOT Score, Nr of matches – number of unique peptides matched to the given sequence, pI/MW theor. was calculated from the downloaded NCBI protein sequence using “Compute pI/MW” tool in Expasy database (www.web.expasy.org). Significant ratio: 1: 70 (S/Q); 2: 50 (S/Q); 3: 40 (S/Q); 4: S (50/70); 5: S (40/70); 6: S (40/50); 7: Q (50/70); 8: Q (40/70); 9: Q (40/50); 10: 70 (Qm/Q); 11: 50 (Qm/Q); 12: 40 (Qm/Q); 13: 70 (Sm/S); 14: 50 (Sm/S); 15: 40 (Sm/S); 16: 70 (Sm/Qm); 17: 50 (Sm/Qm); 18: 40 (Sm/Qm); 19: Sm (50/70); 20: Sm (40/50); 21: Sm (40/70); 22: Qm (50/70); 23: Qm (40/50); 24: Qm (40/70) where Q means Quintus, S means Septima, Qm means aphid-treated Quintus, Sm means aphid-treated Septima, 40 means 40% SWC, 50 means 50% SWC, 70 means 70% SWC; ↑ means an increased rel. abundance while ↓ means decreased rel. abundance.
