# Supplementary figures and images for: Proteomic responses of two spring wheat cultivars to the combined water deficit and aphid (Metopolophium dirhodum) treatments
Source: Front Plant Sci. 2022 Nov 14;13:1005755. doi: 10.3389/fpls.2022.1005755 (PMC9704420; doi:10.3389/fpls.2022.1005755)

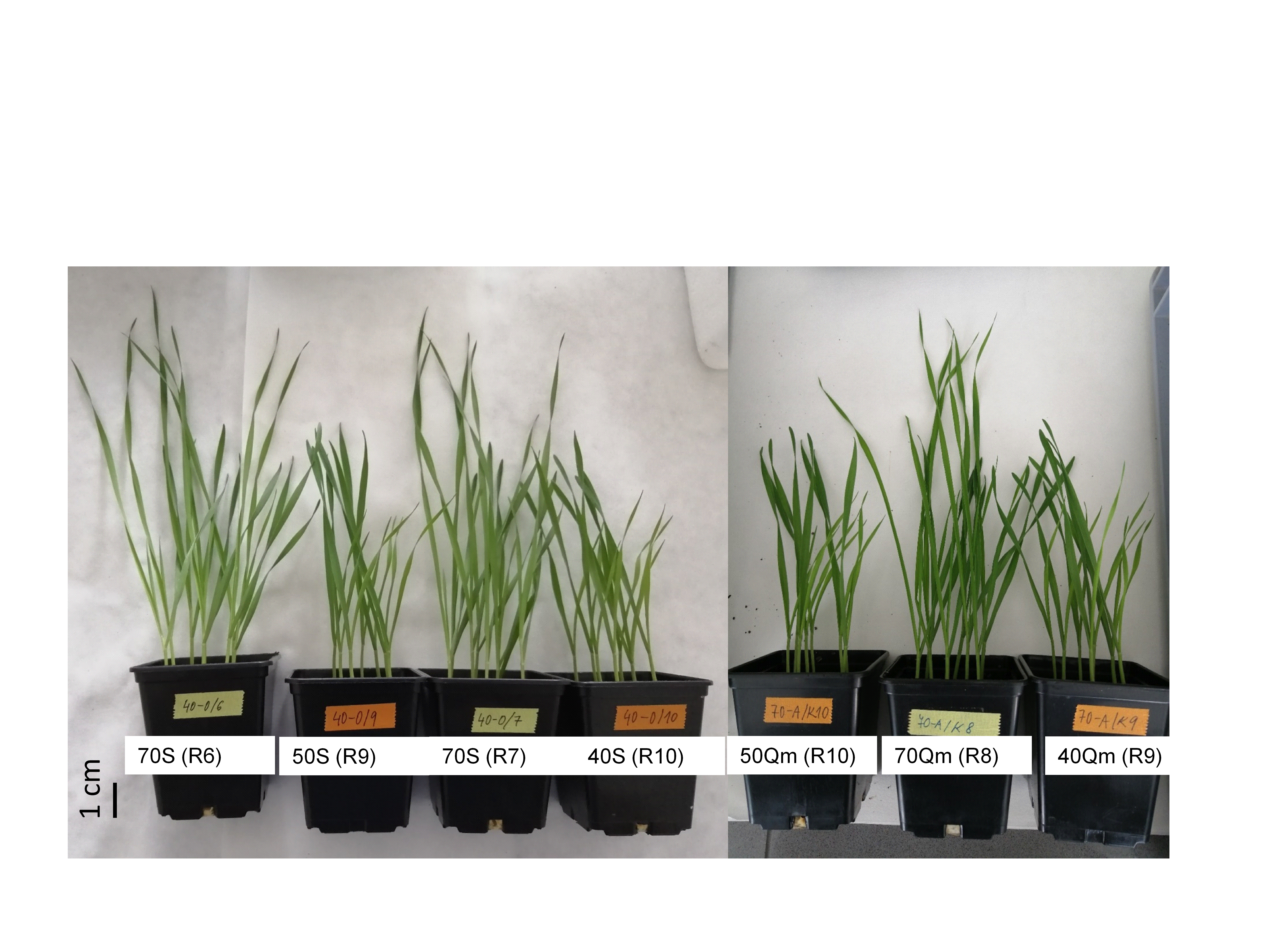

Supplement: Supplementary Figure 1 — Representative image of plants used for the experiments. Q means Quintus, S means Septima, m means treatment with M. dirhodum, 70 means 70% SWC, 50 means 50% SWC, 40 means 40% SWC, R means replicate. [file Image_1.tif]

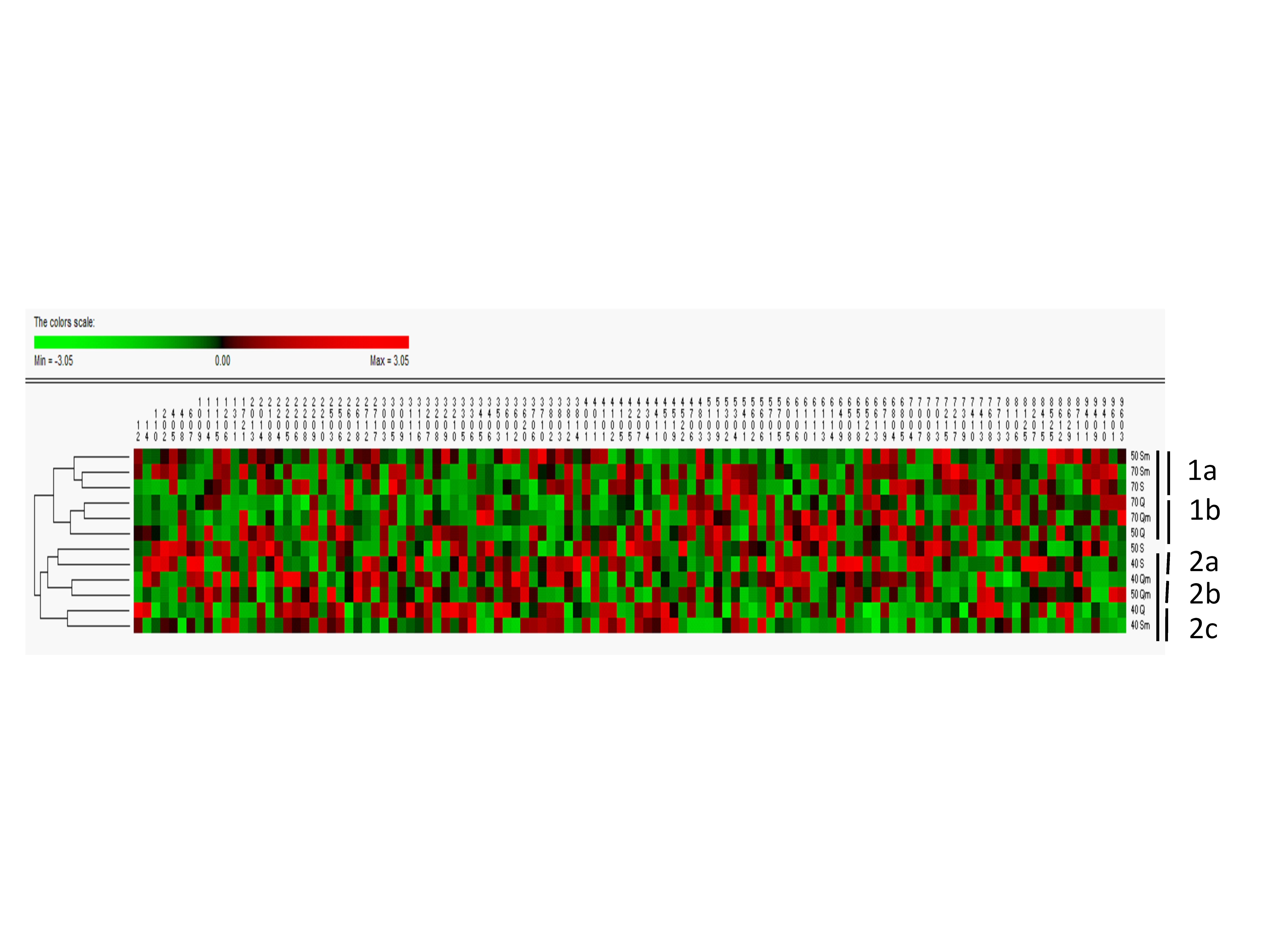

Supplement: Supplementary Figure 2 — Representative images of 2D-DIGE gels of the 12 experiment variants. [file Image_2.tif]

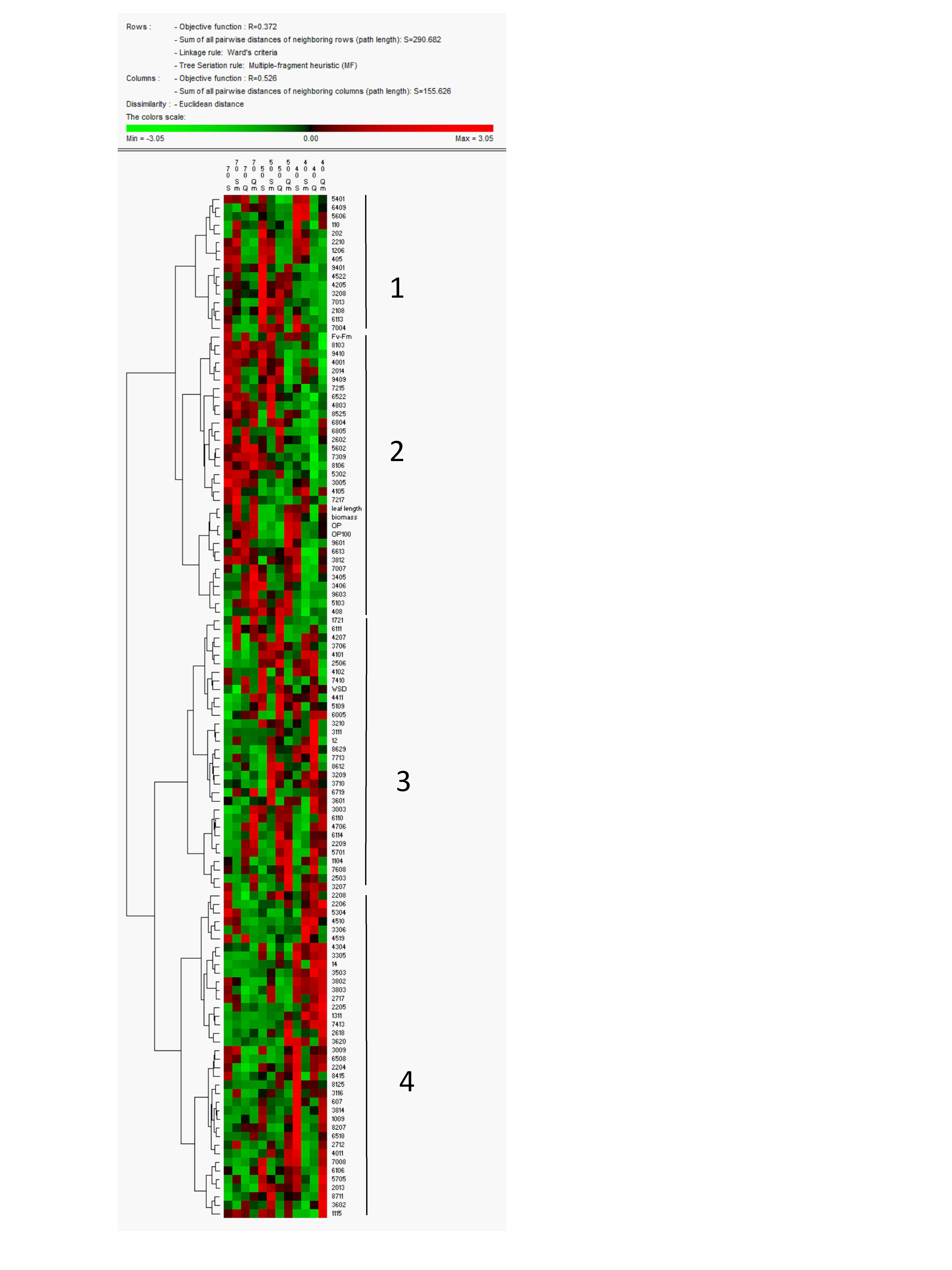

Supplement: Supplementary Figure 3 — Cluster analysis of 113 differentially abundant proteins (DAPs) showing the clustering of 12 experiment variants. Abbreviations: 40 – 40% soil water content (SWC), 50 – 50% SWC, 70 – 70% SWC, m – M. dirhodum infestation, Q – Quintus, S – Septima. [file Image_3.tif]
