# Supplementary material for: Proteomic responses of two spring wheat cultivars to the combined water deficit and aphid (Metopolophium dirhodum) treatments
Source: Front Plant Sci. 2022 Nov 14;13:1005755. doi: 10.3389/fpls.2022.1005755 (PMC9704420; doi:10.3389/fpls.2022.1005755)
Supplement: Supplementary Figure 4 — Protein-protein interactions networks drawn by STRING online tool (https://www.string-db.org) for the identified proteins. [file Table_4.docx]

Protein-protein interaction (PPI) networks for the identified proteins drawn by STRING (accessed 30th August 2022). The identified protein is symbolized by the red node.

Legend:

Nodes: red node – the identified protein used as a query in STRING database

Edges: blue – from curated databases; red – gene fusions; pink - experimentally determined; pale green – textmining; green – gene neighborhood; pale blue – protein homology; dark blue – gene co-occurrence


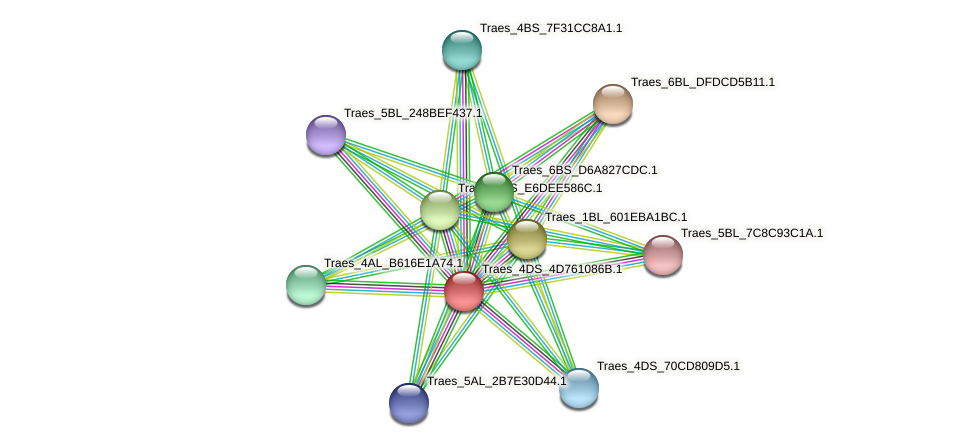


Ssp 12: XP_040242492.1 (*Aegilops tauschii* ssp. *strangulata*), CBS domain-containing protein CBSX3, mitochondrial; interacting partners other than uncharacterized proteins: Glutamine amidotransferase type-1


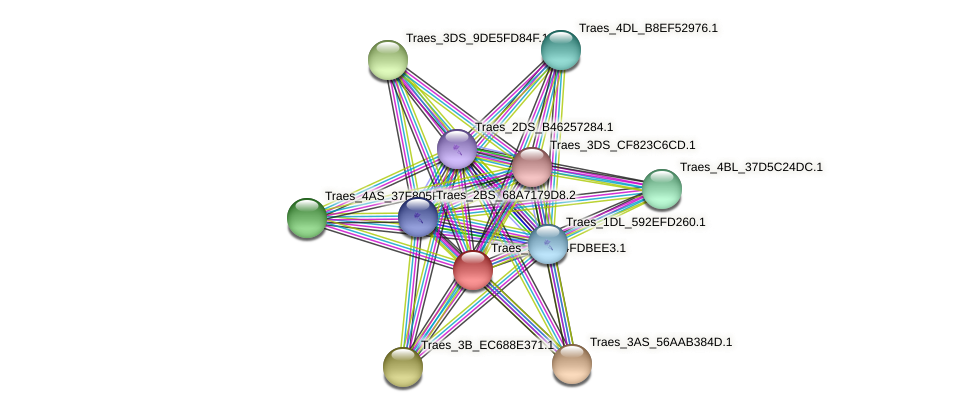


Ssp 110: XP_037413079.1 mitochondrial porin 3; interacting partners other than uncharacterized proteins: ATP synthase ε subunit


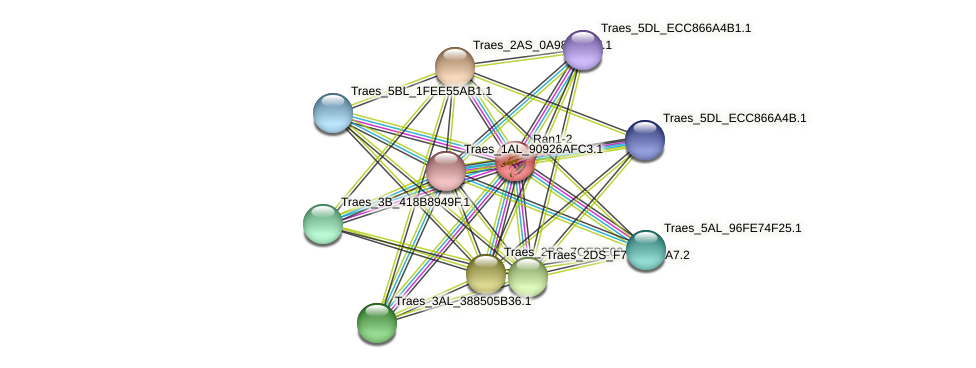


Ssp 202: XP_020174223.1 Ran 2 small GTP-binding protein nuclear; interacting partners other than uncharacterized proteins: nuclear transport factor 2 (NTF2), tRNA nucleus export receptor


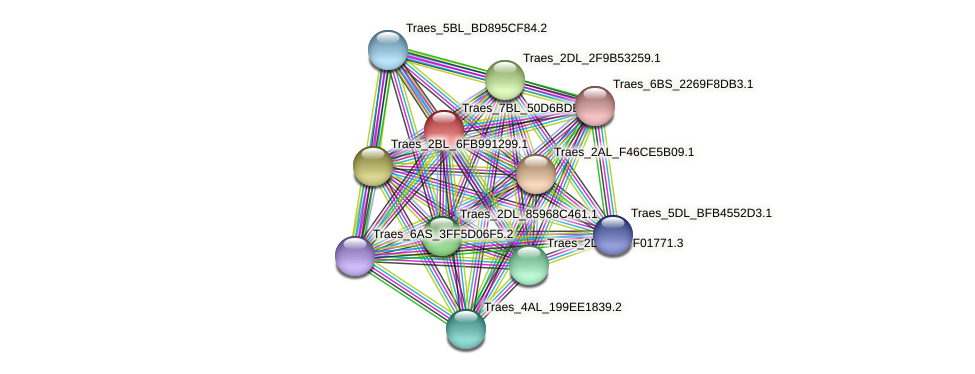


Ssp 202: AAK01176.1 RNA-binding protein


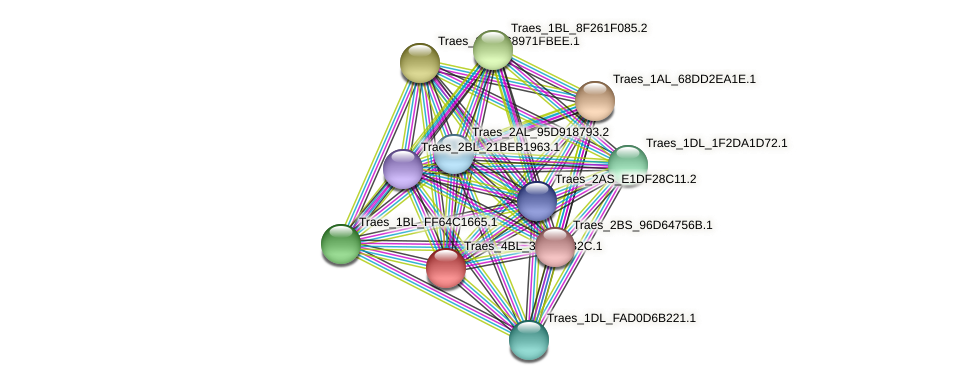


Ssp 1206: KAE8800160.1 eIF-3 subunit i


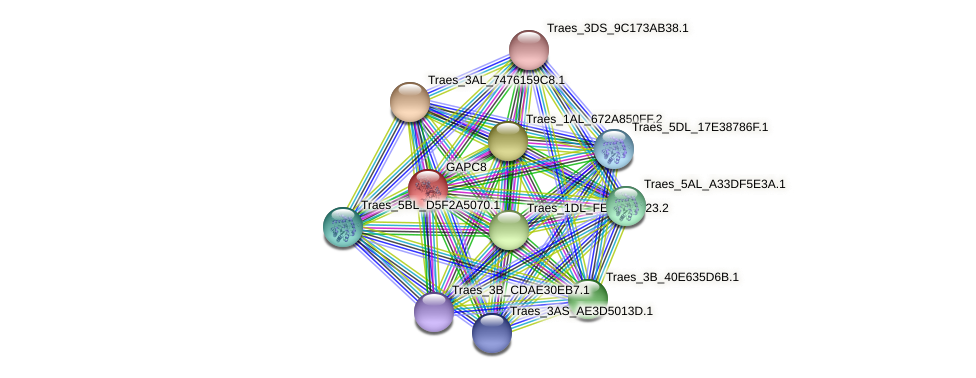


Ssp 1206: GAPDH


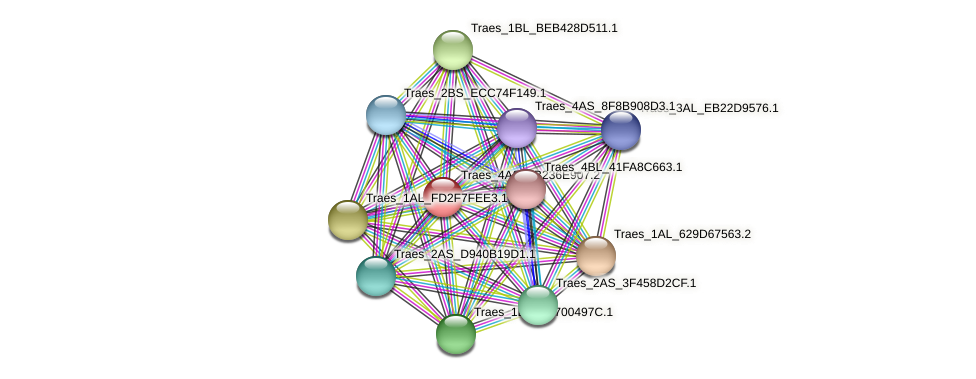


Ssp 2014: KAE8790324.1 40S ribosomal protein S21; interacting partners other than uncharacterized proteins: 40S ribosomal protein SA, ribosomal protein eL38


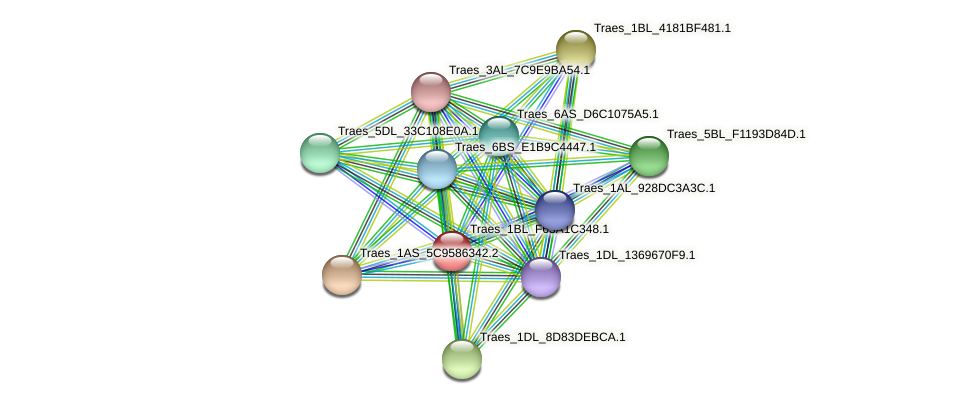


Ssp 2108: XP_020198028.1 NAD(P)H dehydrogenase (quinone) FQR1; interacting partners other than uncharacterized proteins: Flavodoxin-like domain containing protein, flavokinase domain containing protein


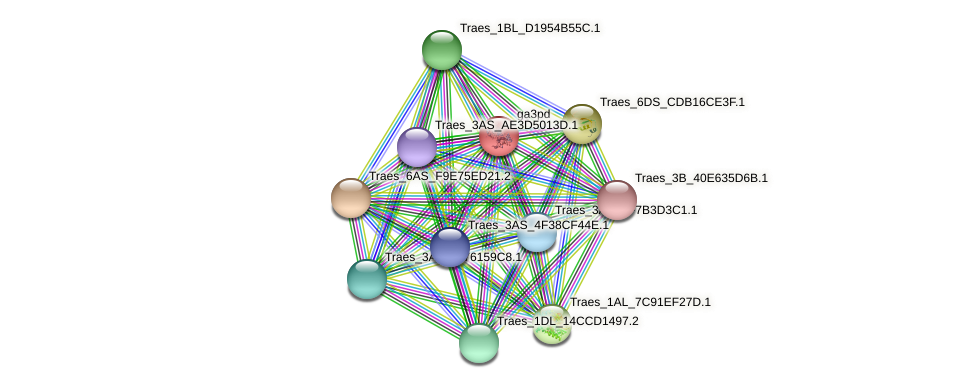


Ssp 2205: ANW11922.1 GAPDH interacting partners other than uncharacterized proteins: Phosphoglycerate kinase


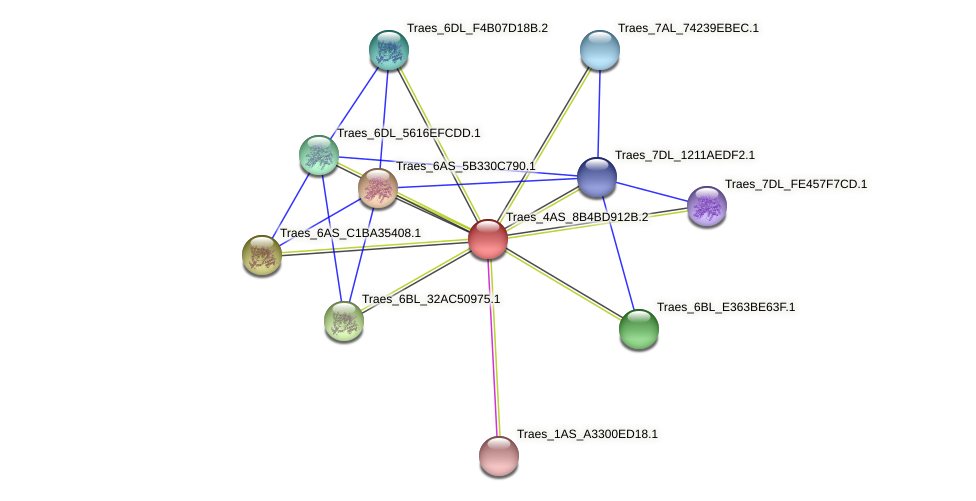


Ssp 2206: XP_037420774.1 ricin b lectin


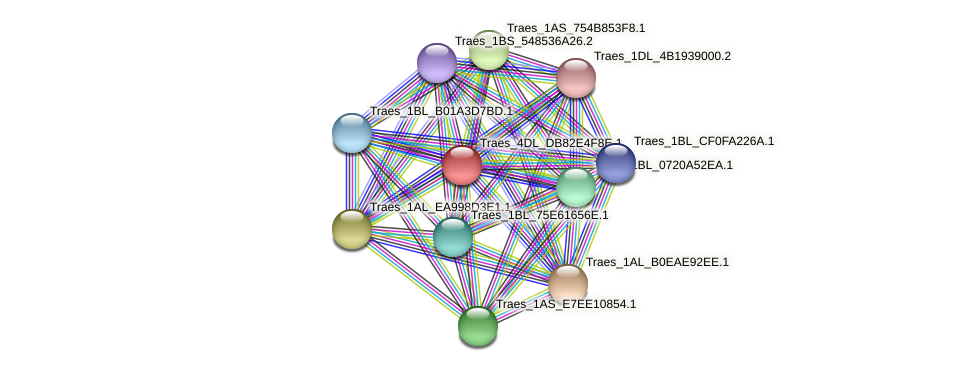
ssp 2210: XP_020167947.1 proteasome subunit alpha type-6; interacting partners other than uncharacterized proteins: Proteasome subunit alpha, beta type


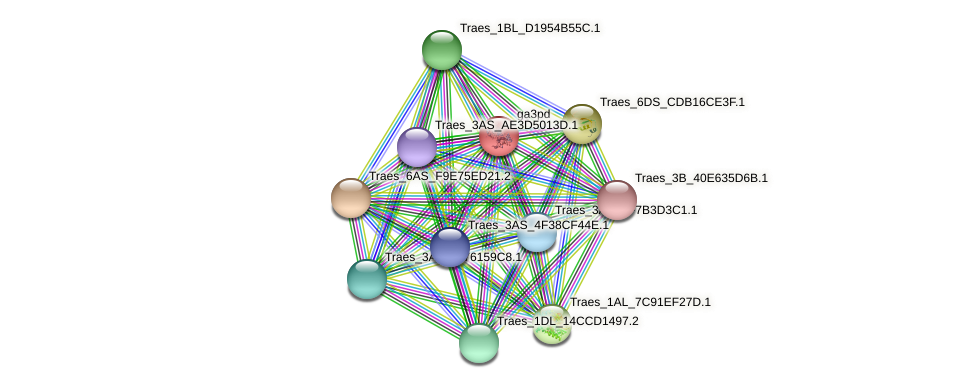


Ssp 2503: EMS54975.1 GAPDH interacting partners other than uncharacterized proteins: Phosphoglycerate kinase (PGK), glyceraldehyde-3-phosphate dehydrogenase (GAPDH), fructose bisphosphate aldolase


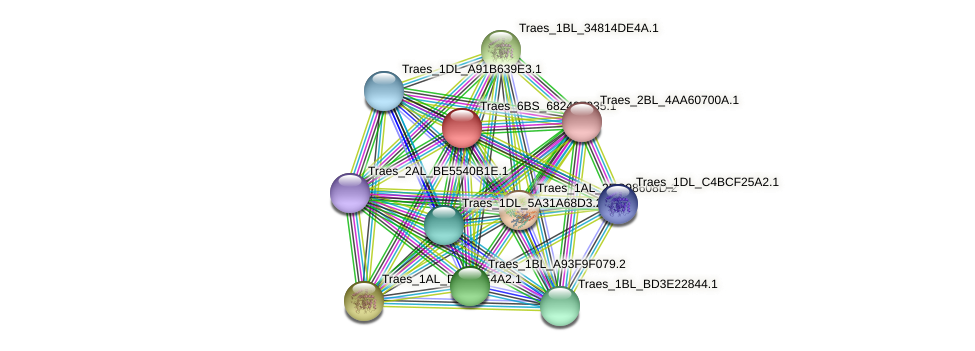


Ssp 2506: XP_037446710.1 citrate synthase; interacting partners other than uncharacterized proteins: Malate dehydrogenase


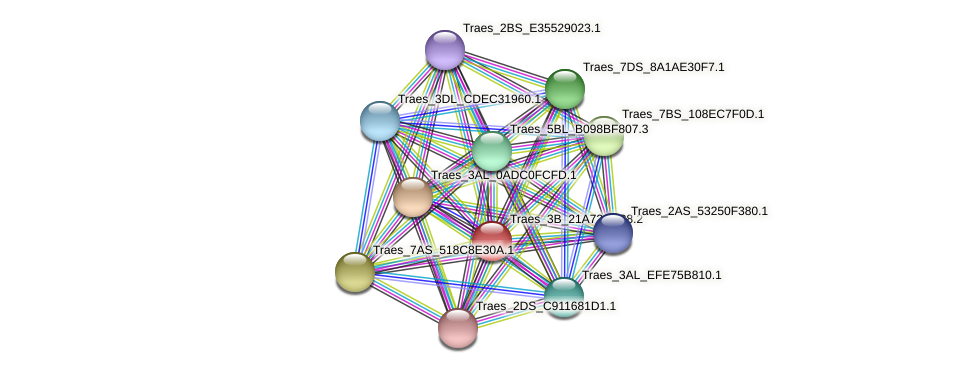


Ssp 2602: KAE8802615.1 mitochondrial peptidase; interacting partners other than uncharacterized proteins: NADH dehydrogenase flavoprotein 1, cytochrome b-c1 subunit 7, cytochrome c domain-containing protein


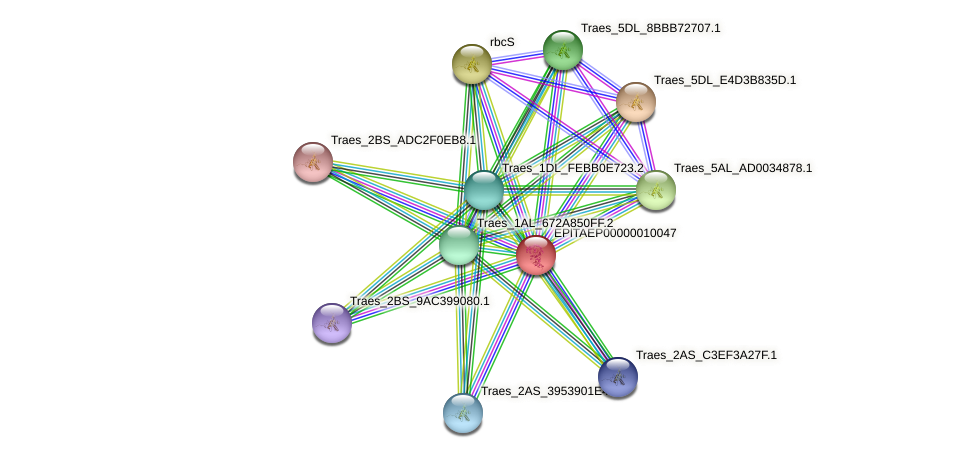


Ssp 2618: AAU11113.1  RubisCO LSU interacting partners other than uncharacterized proteins: RbcL, RbcS, PGK (RubisCO large chain, RubisCO small chain, phosphoglycerate kinase)


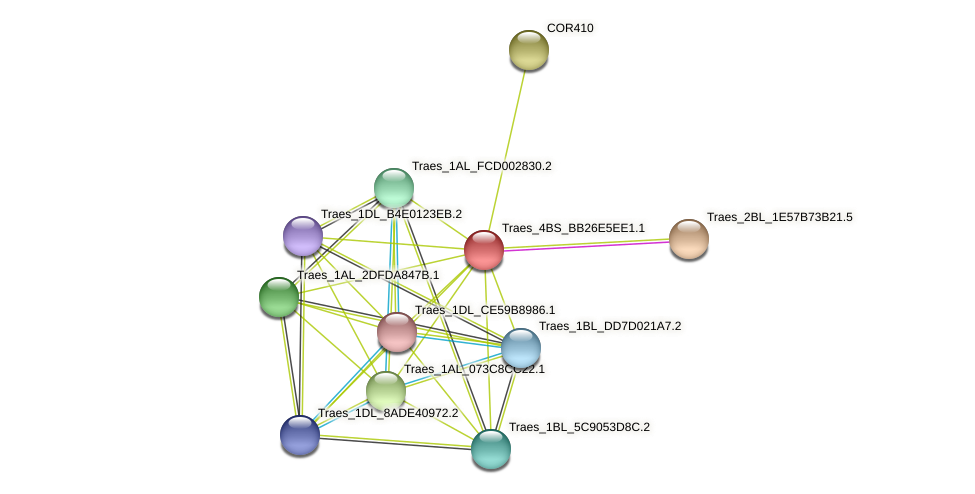


Ssp 3005: QPO15911.1 LEA19; interacting partners other than uncharacterized proteins: WCOR410 dehydrin, anaphase complex subunit 10


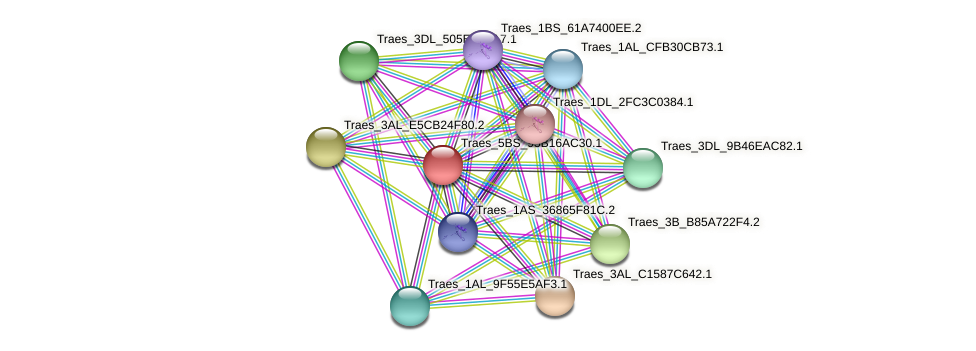


Ssp 3009: XP_037432389.1  E2 ubiquitin conjugating enzyme ; interacting partners other than uncharacterized proteins: Ubiquitin, ubiquitin-like domain containing protein, ubiquitin-conjugating protein family


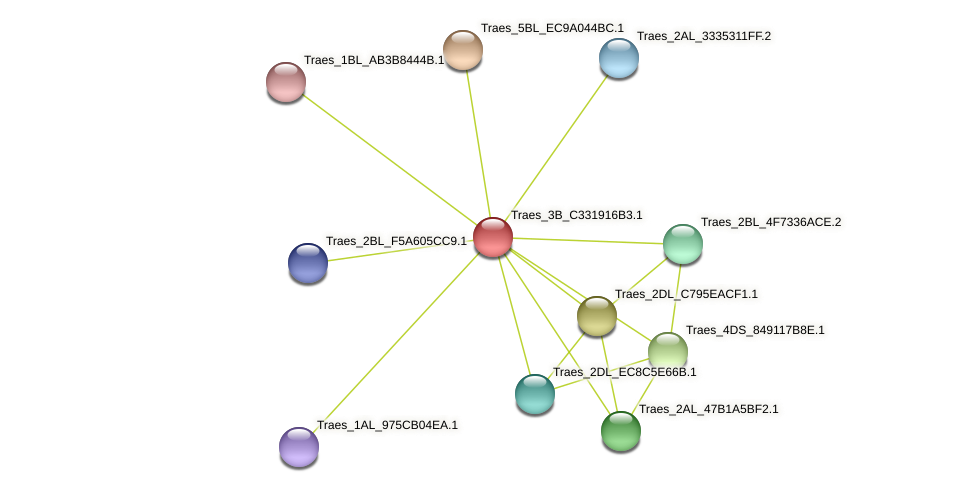


Ssp 3111: AAG00428.1 germin D; interacting partners other than uncharacterized proteins: ATP-dependent protease proteolytic subunit


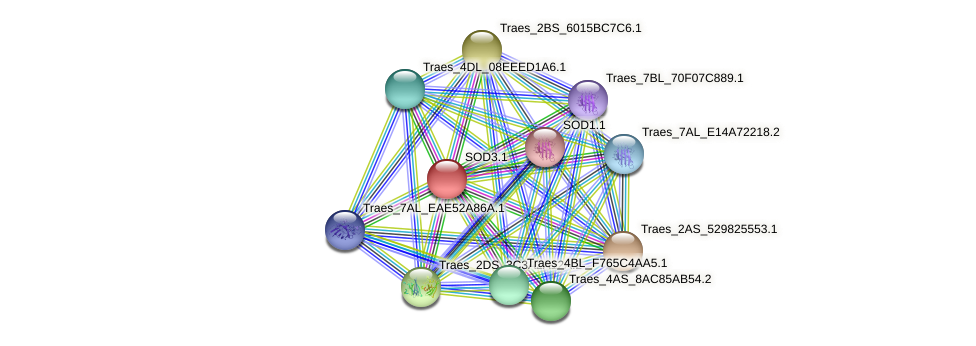


Ssp 3111: QBZ38485.1 MnSOD; interacting partners other than uncharacterized proteins: Cu/Zn-SOD


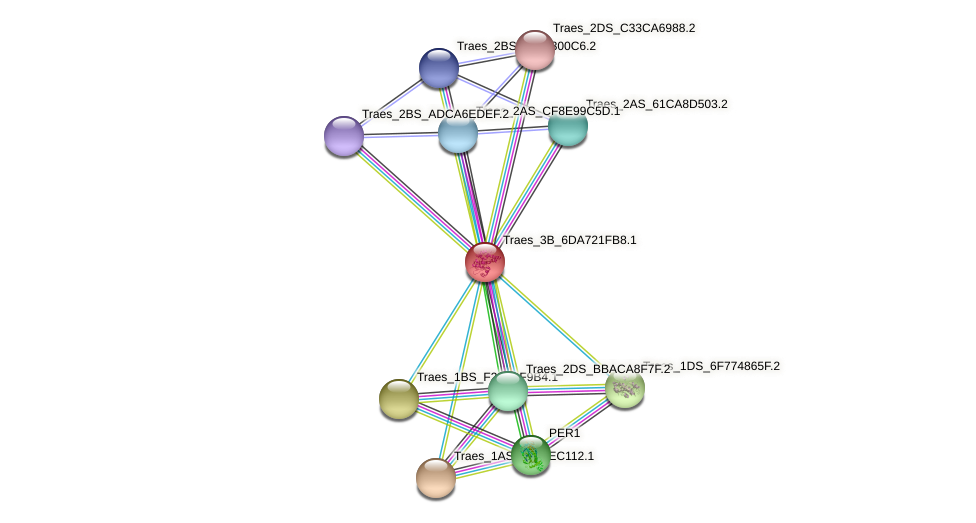


Ssp 3207: XP_037414204.1  GST1; interacting partners other than uncharacterized proteins: 1-Cys Prx (PER1), EF1_GNE domain containing protein of EF1-beta/EF1-delta family


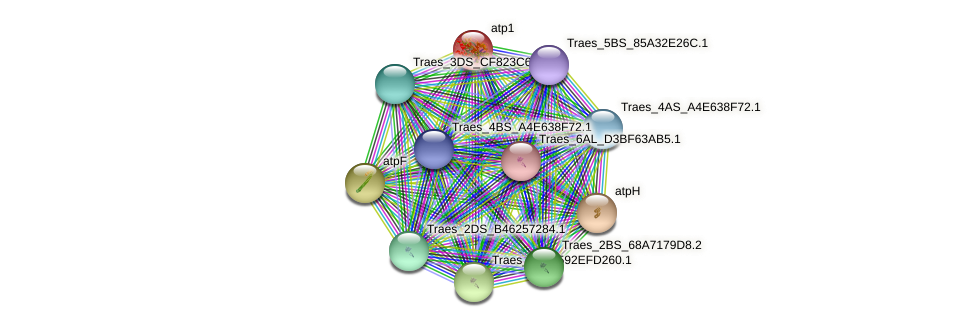


Ssp 3306: XP_040248654.1 ATP synthase subunit alpha; interacting partners other than uncharacterized proteins: Atp1, AtpF, atpH, ATP synthase ε subunit


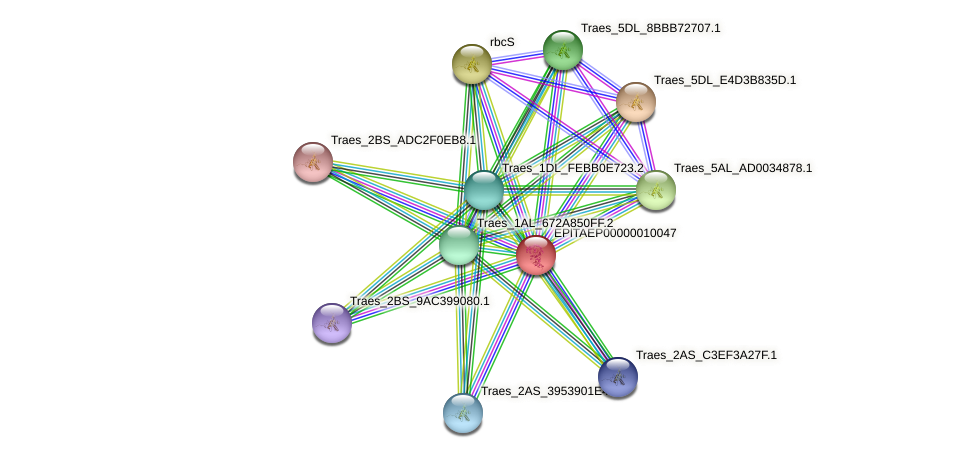


Ssp 3405: ASD37885.1 RubisCO LSU; interacting partners other than uncharacterized proteins: RubisCO small chain, PGK


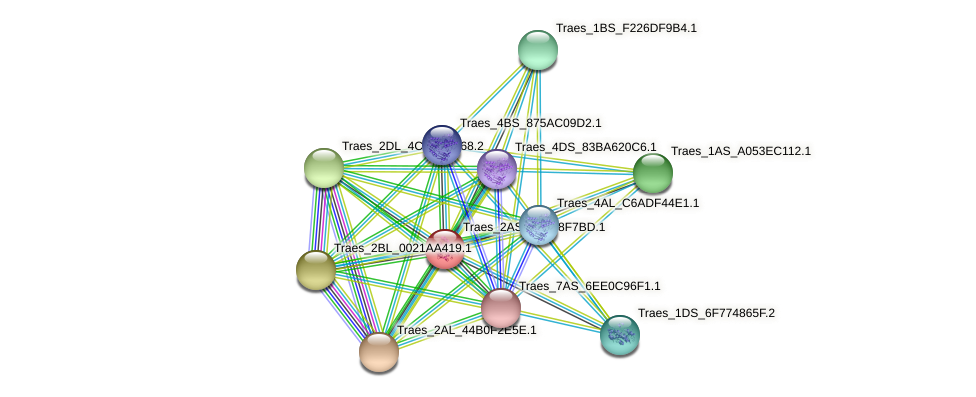


Ssp 3406: XP_037475587.1 ascorbate peroxidase; interacting partners other than uncharacterized proteins: Bifunctional dihydrofolate reductase thymidylate synthase, Pyr_redox 2 domain containing protein, GST superfamily


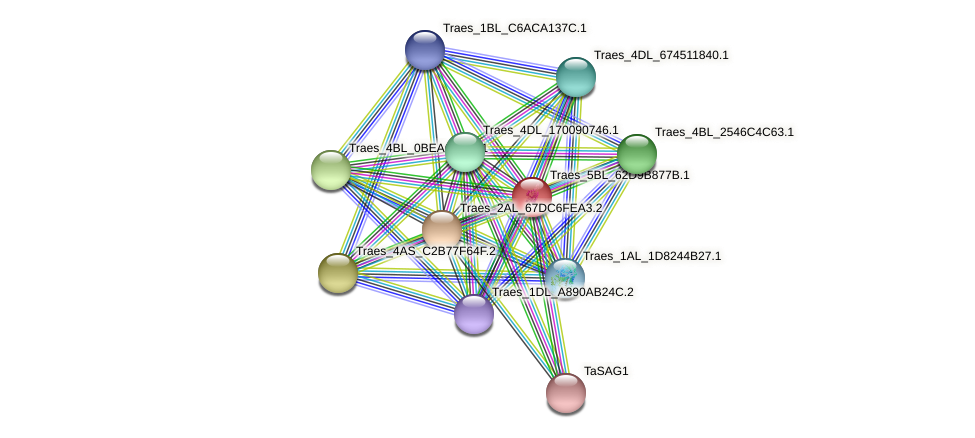


Ssp 3503: ADJ67791.1 aldehyde dehydrogenase; interacting partners other than uncharacterized proteins: SAG1 aminotransferase class-III pyridoxal phosphate-dependent aminotransferase


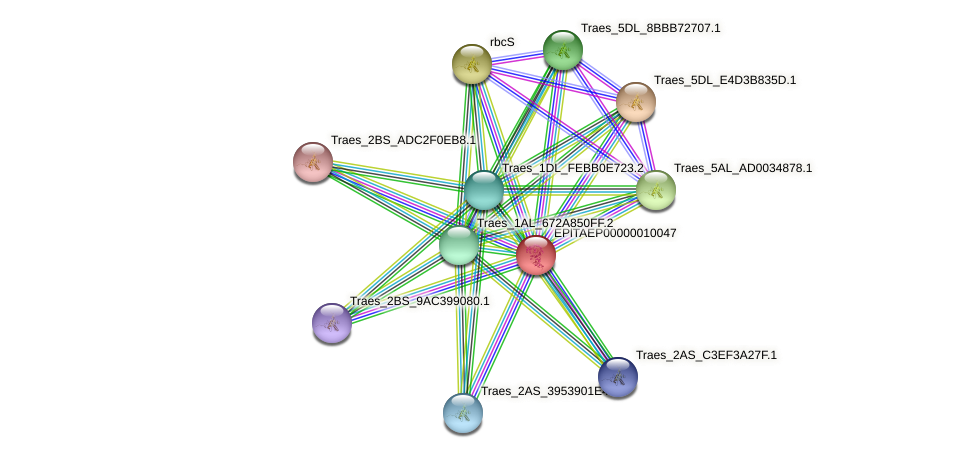


Ssp 3620: AAU11113.1 RubiscO LSU; interacting partners other than uncharacterized proteins: RbcL, RbcS, PGK (RubisCO large chain, RubisCO small chain, phosphoglycerate kinase)


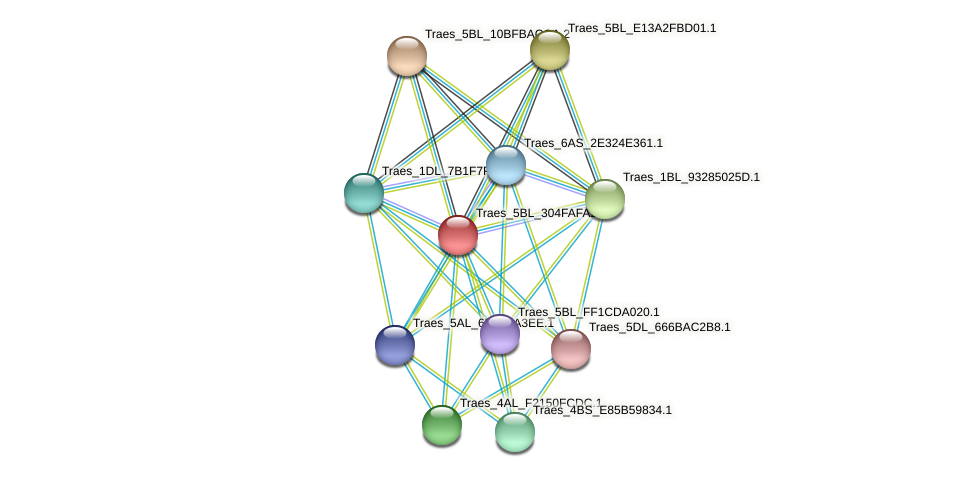


Ssp 3803: QNV69782.1 lipoxygenase 2 LOX2; interacting partners other than uncharacterized proteins: Cytochrome P450, LAH – lipolytic acyl hydrolase


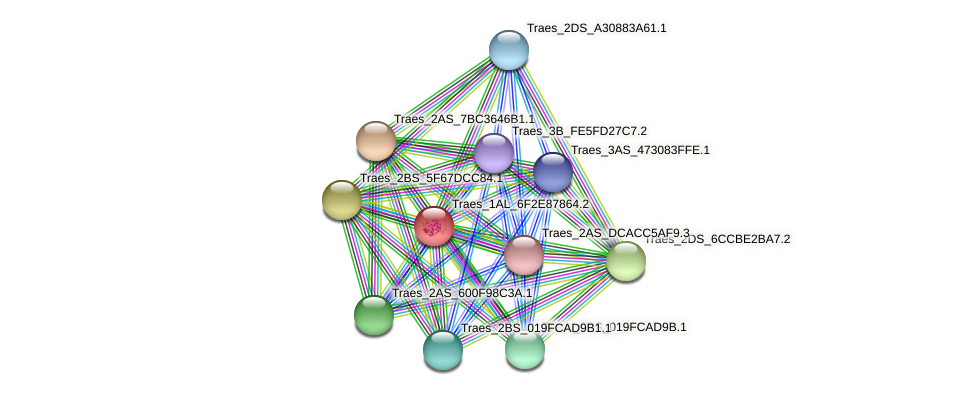


Ssp 4011: EMS63724.1 nucleoside diphosphate kinase 1 NDPK1; interacting partners other than uncharacterized proteins: Adenylate kinase, thymidylate kinase, ump-cmp kinase (pyrimidine monophosphate kinase)


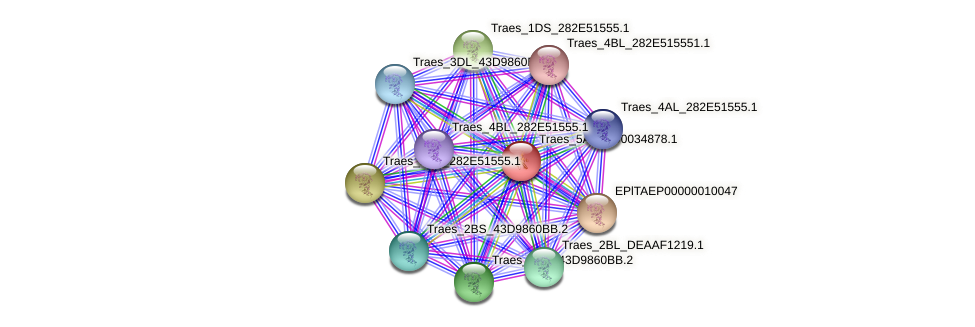


Ssp 4011: KAE8790036.1 RubisCO SSU; interacting partners other than uncharacterized proteins: RubisCO LSU


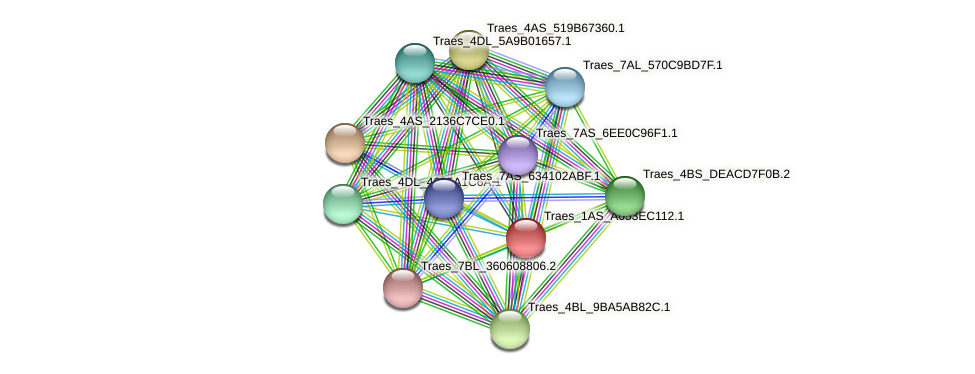


Ssp 4101: XP_020196644.1  GST glutathione-S-transferase; interacting partners other than uncharacterized proteins: Glutathione peroxidase GPX, Pyr_redox2_domain-containing protein


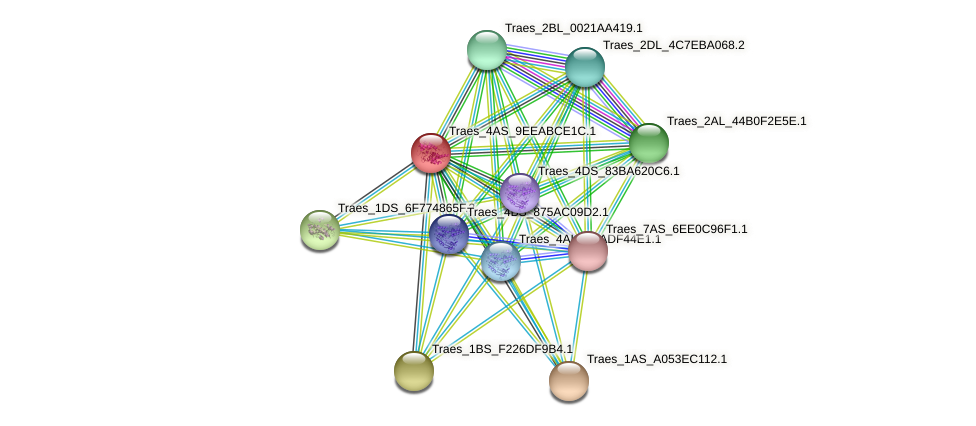


Ssp 4207: XP_020172367.1 ascorbate peroxidase APX; interacting partners other than uncharacterized proteins: Bifunctional dihydrofolate reductase thymidylate synthase, Pyr_redox 2 domain containing protein, GST superfamily


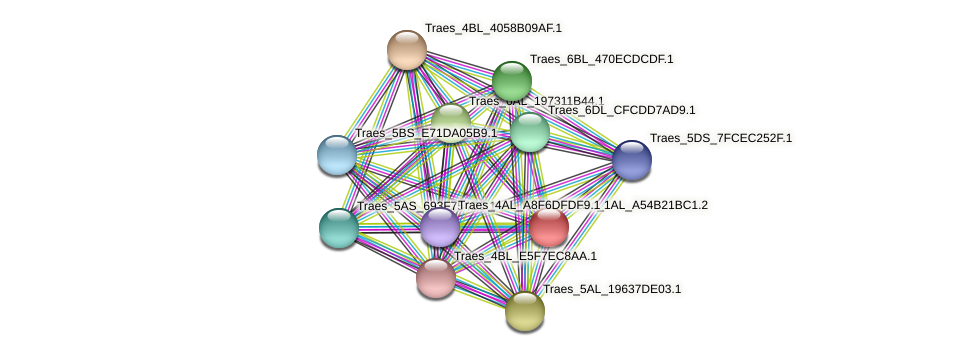


Ssp 4304: KAE8791495.1 serine-threonine kinase; interacting partners other than uncharacterized proteins: Adaptor protein complex AP-2, AP sigma subunit, Mhd domain


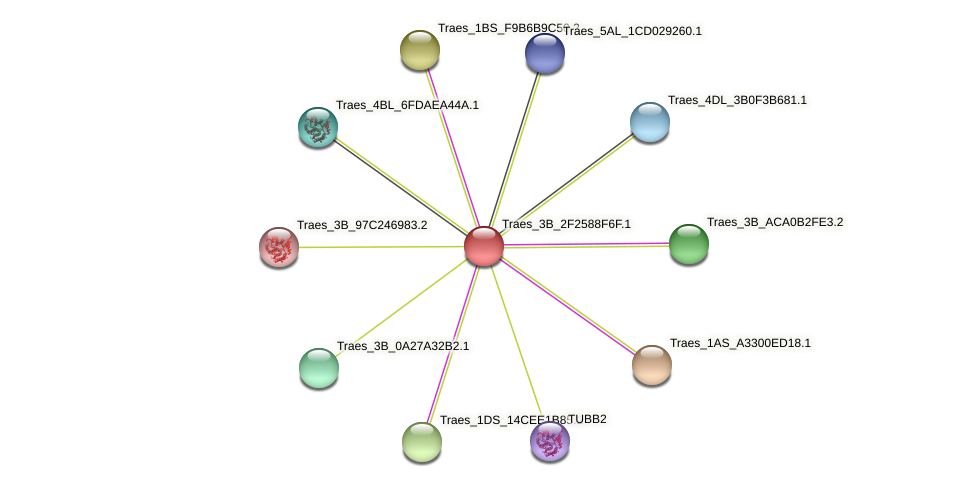


Ssp 4411: XP_020183874.1 ricin b lectin; interacting partners other than uncharacterized proteins: Tubulin beta-2


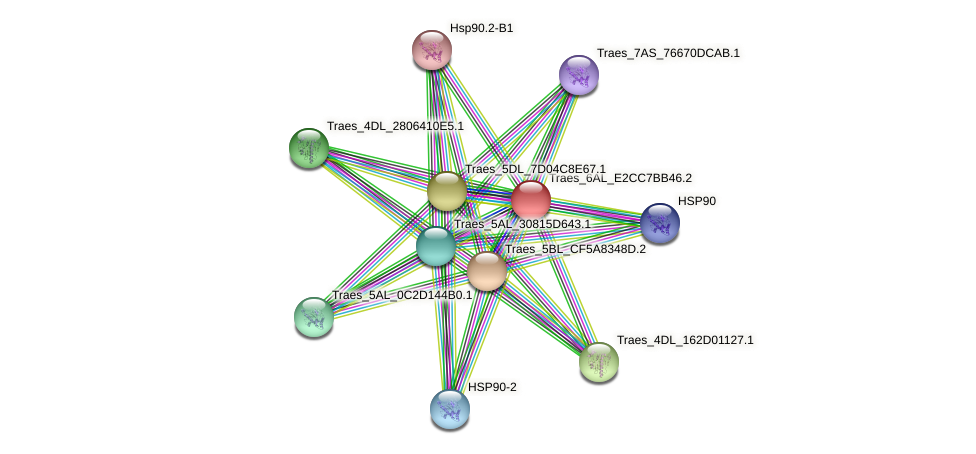


Ssp 4519: EMS50129.1 Hsc70; interacting partners other than uncharacterized proteins: HSP90, HSP90-2


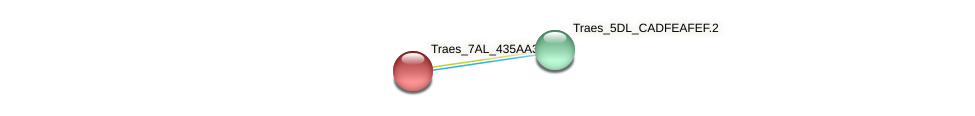


Ssp 4519: XP_020190543.1 peroxidase 1; no interacting proteins other than uncharacterized ones


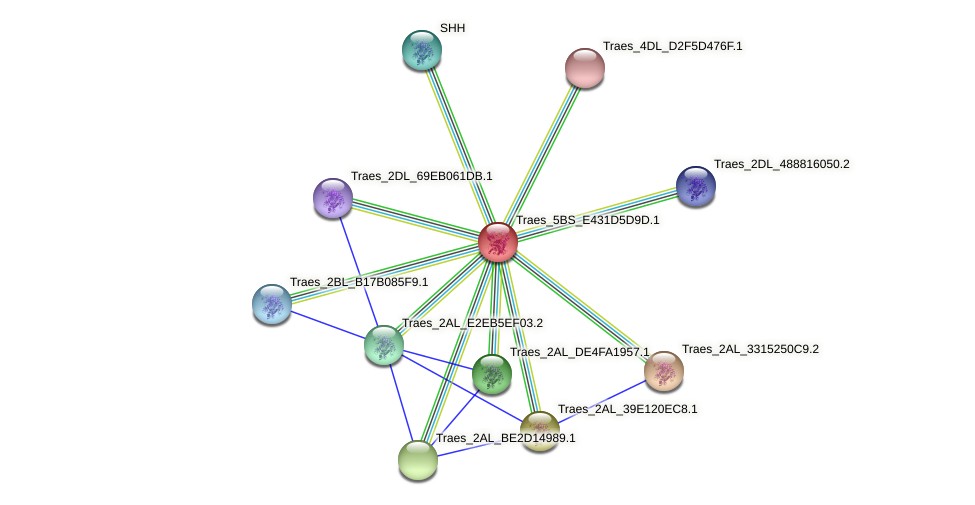


Ssp 4706: XP_037439131.1 5-methyltetrahydropteroyltriglutamate--homocysteine methyltransferase 1-like; interacting partners other than uncharacterized proteins: SHH = adenosylhomocysteinase


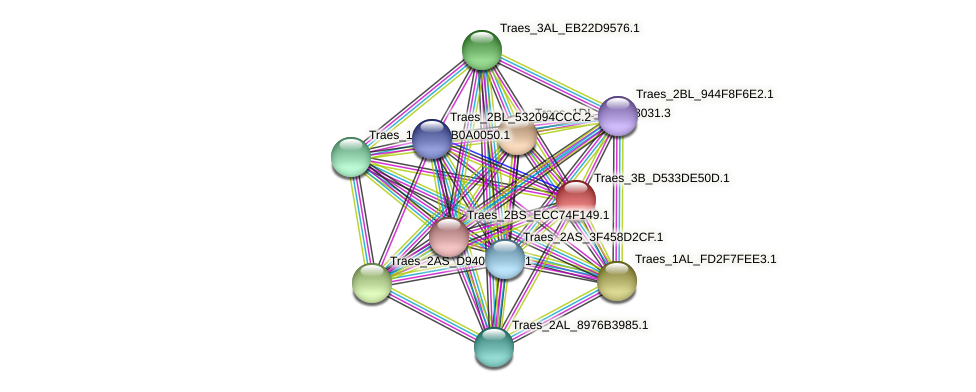


Ssp 5103: XP_037425140. GTP-binding protein subunit beta; interacting partners other than uncharacterized proteins: 60S ribosomal protein L29, 40S ribosomal protein SA


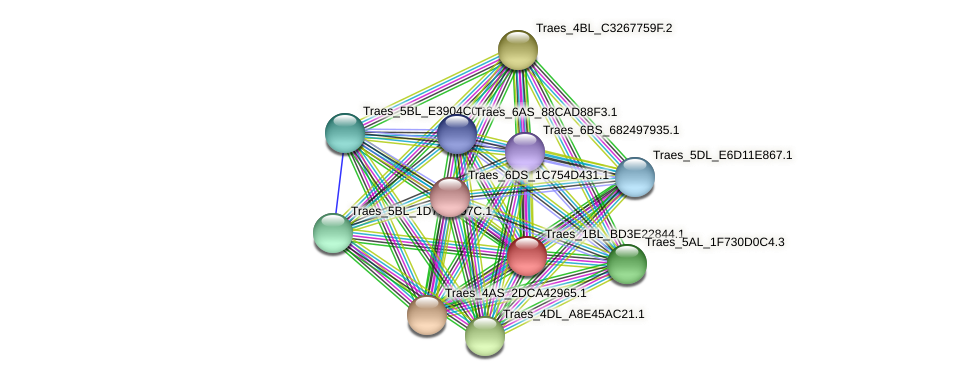


Ssp 5103: XP_037473639.1 malate dehydrogenase; interacting partners other than uncharacterized proteins: Malate dehydrogenase, citrate synthase


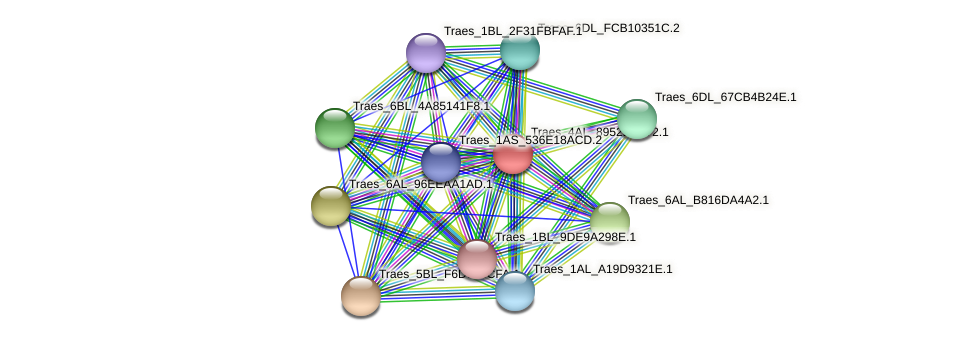


Ssp 5103: KAE8779219.1 electron transfer flavoprotein subunit alpha, mitochondrial; interacting partners other than uncharacterized proteins: Electron transfer flavoprotein-ubiquinone oxidoreductase, Etf-domain containing protein


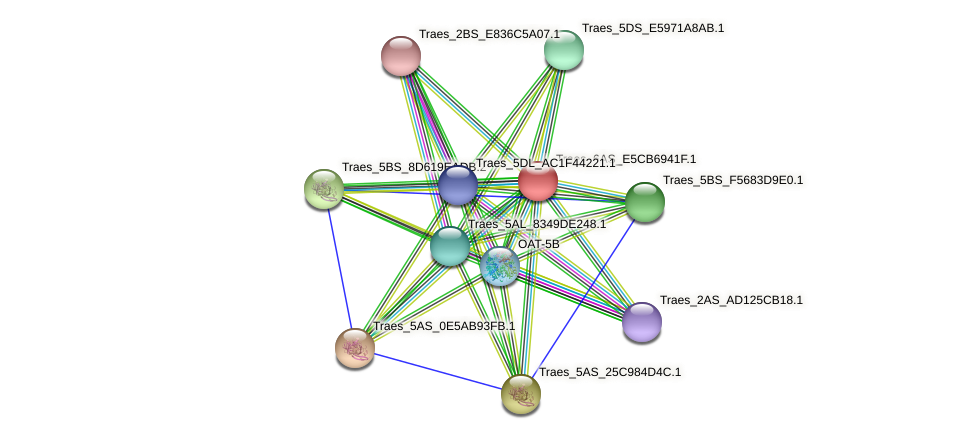


Ssp 5302: EMS64657.1   putative diaminopimelate decarboxylase, chloroplastic; interacting partners other than uncharacterized proteins: Class-III pyridoxal phosphate-dependent aminotransferase


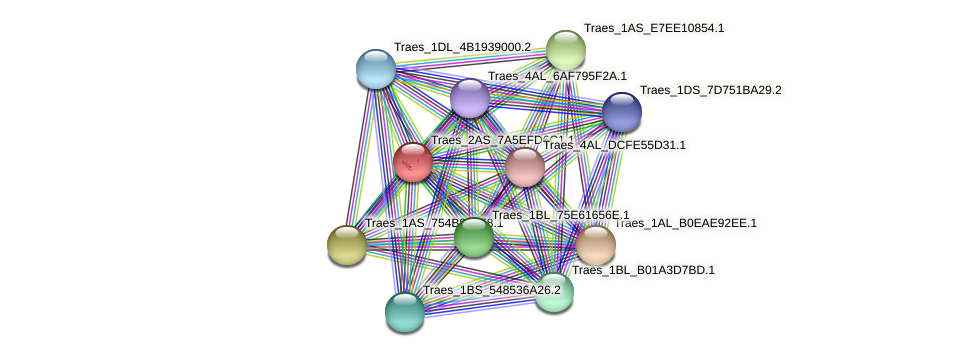


Ssp 5304: EMS47104.1 26S protease; interacting partners other than uncharacterized proteins: Proteasome subunit alpha, beta type


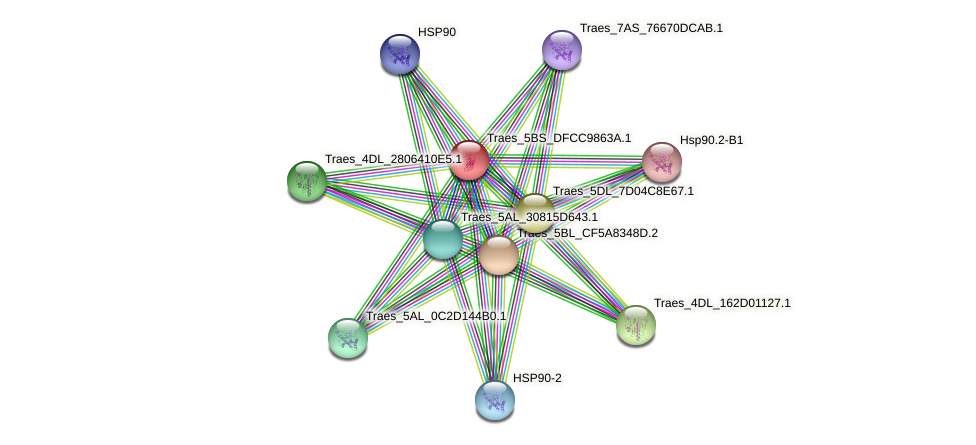


Ssp 5304: XP_037439714.1   heat shock-cognate protein 70 Hsc70; interacting partners other than uncharacterized proteins: HSP90, HSP90-2


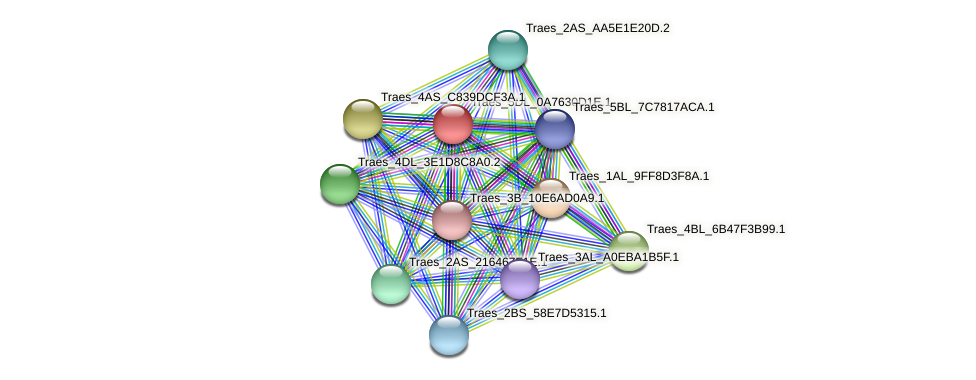


Ssp 5401: XP_020173165.1 UDP-glucose 6-dehydrogenase 4; interacting partners other than uncharacterized proteins: UDP-glucose-6-dehydrogenase, epimerase domain-containing protein, NAD(P) domain containing protein


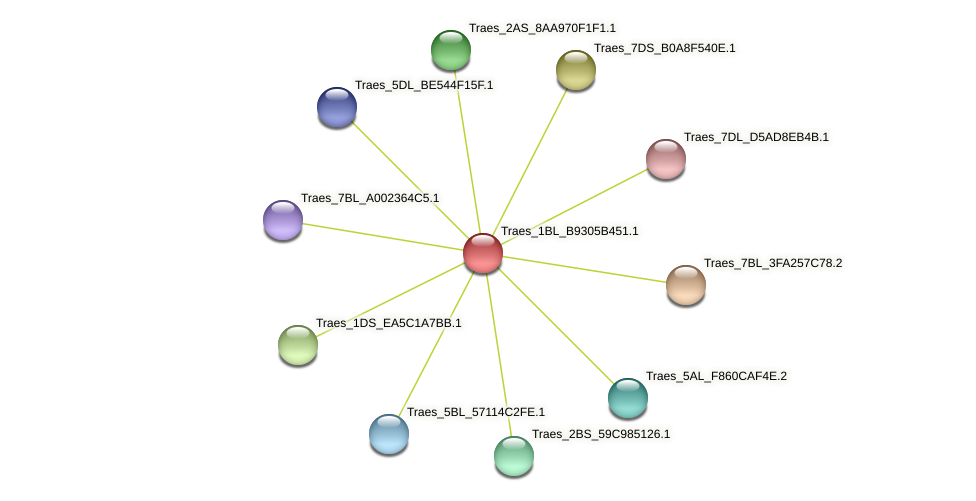


Ssp 6106: XP_020157388.1 isoflavone reductase homolog IRL IRL ; interacting partners other than uncharacterized proteins: Hth cro/c1 type domain containing protein


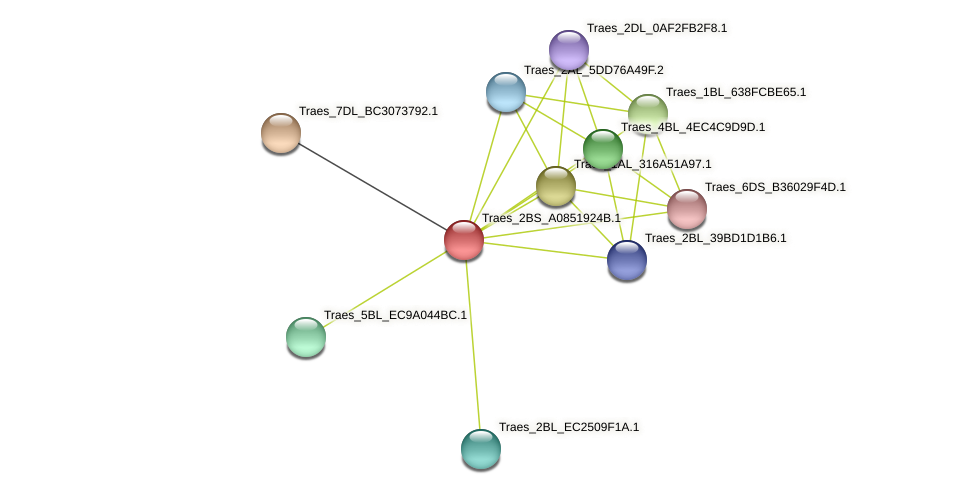


Ssp 6106: XP_037460771.1 germin 8-14; interacting partners other than uncharacterized proteins: Ab hydrolase-1 domain-containing protein


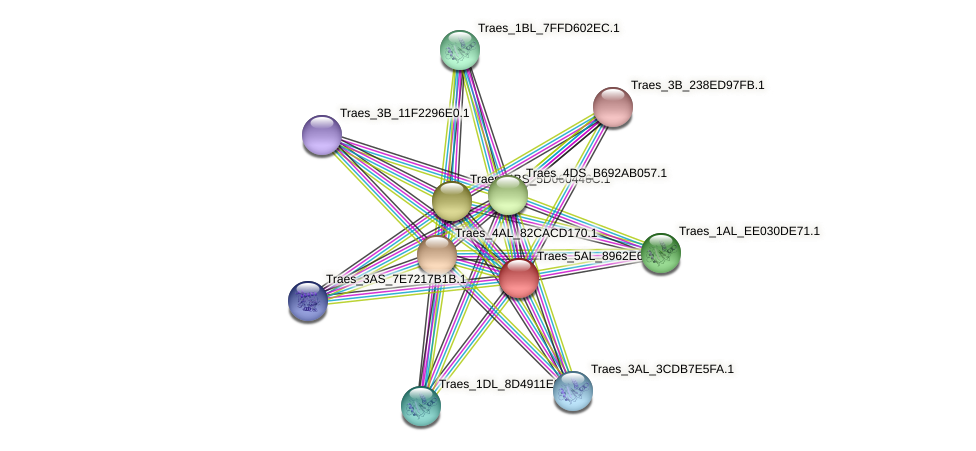


Ssp 6110, 6111: XP_037436859.1 actin depolymerizing factor 4 ADF4; interacting partners other than uncharacterized proteins: Profilin, adenylyl cyclase associated proteins CAP family


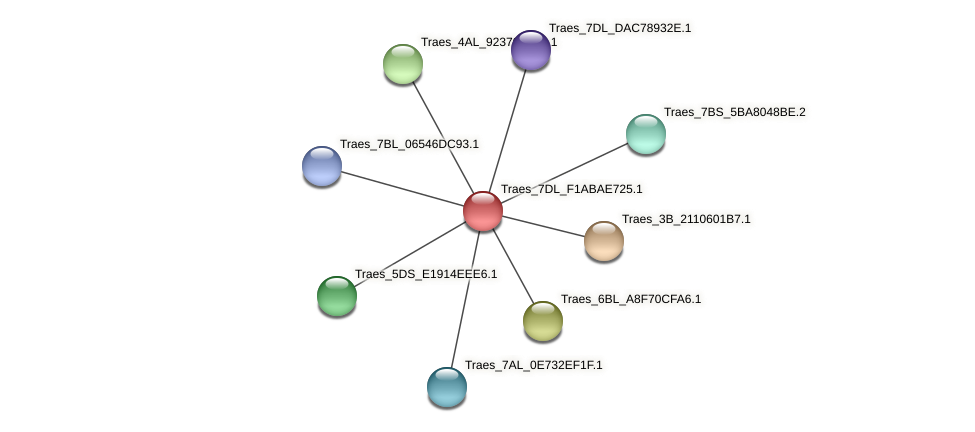


Ssp 6113: XP_037458661.1 uncharacterized protein; no interacting partners other than uncharacterized proteins


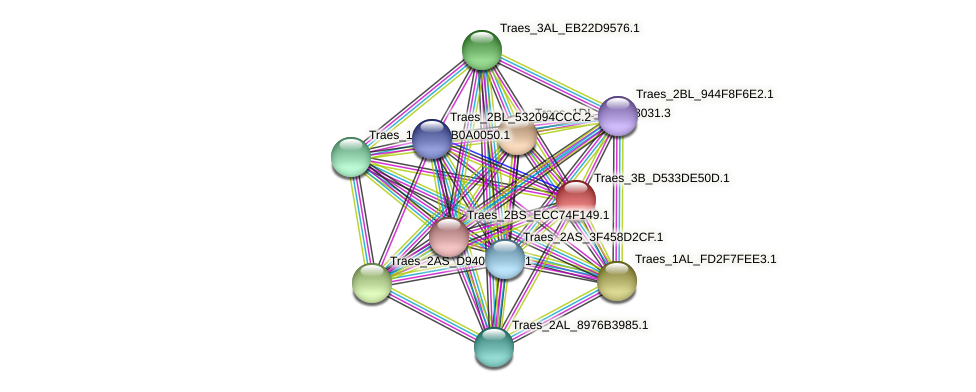


Ssp 6409: XP_020197659.1 GTP binding protein subunit beta; interacting partners other than uncharacterized proteins: eIF3, 60S ribosomal protein L29, ribosomal protein eL38, 40S ribosomal protein SA


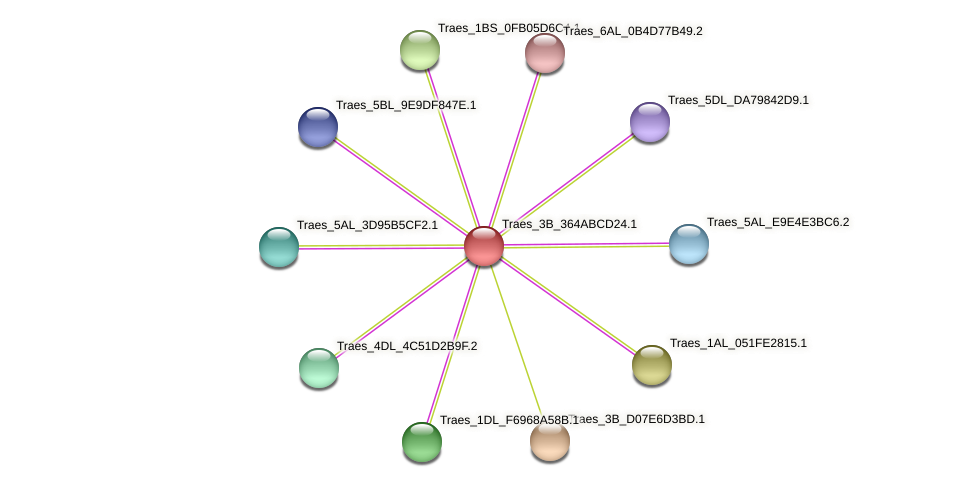


Ssp 6409: XP_037412554.1 transcription factor Pur alpha 1; no other interacting partners than uncharacterized proteins


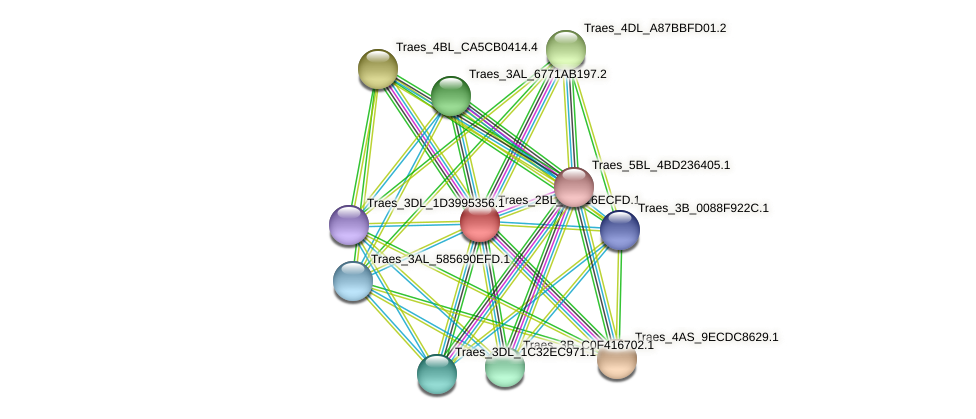


Ssp 6613: XP_037479697.1  D-3-phosphoglycerate dehydrogenase 1, chloroplastic-like; interacting partners other than uncharacterized proteins: D-3-phosphoglycerate dehydrogenase, phosphoserine aminotransferase


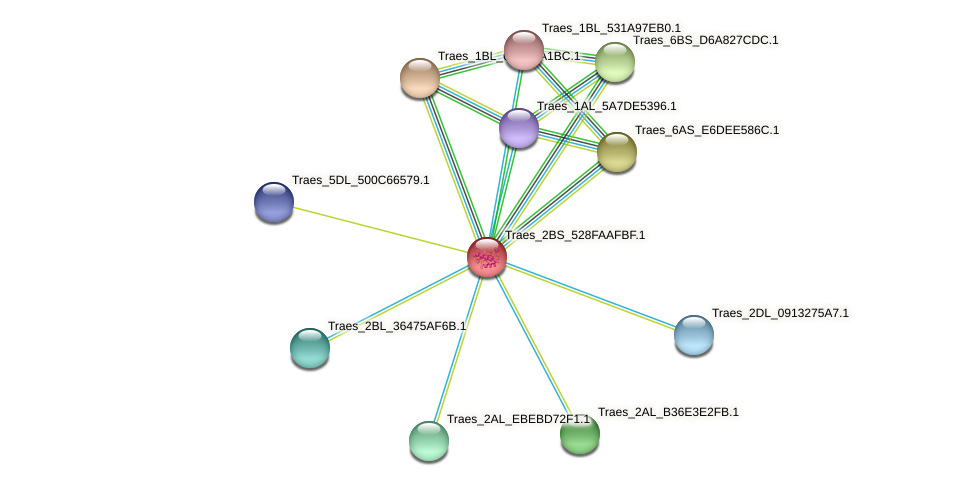


Ssp 7004: KAE8774345.1 reactive intermediate deaminase A, chloroplastic; interacting partners other than uncharacterized proteins: Pyrophosphatase, WD repeat region


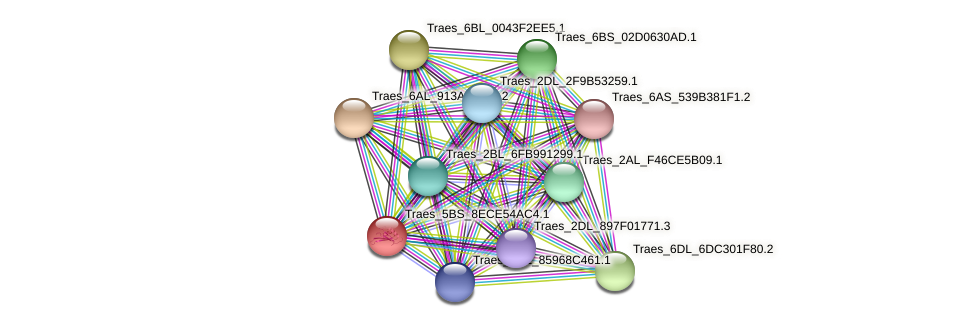


Ssp 7008: XP_020162185.1 glycine-rich protein GRP; no other interacting partners than uncharacterized proteins


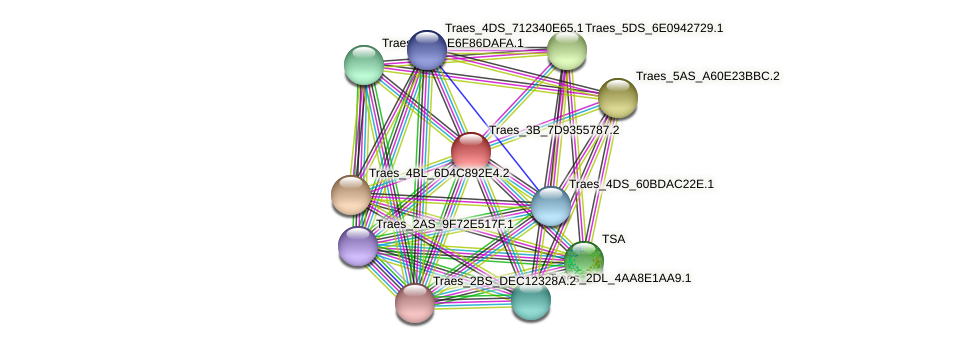


Ssp 7013: XP_037413838.1 2-Cys peroxiredoxin; interacting partners other than uncharacterized proteins: Ubiquitin-related modifier 1, 2-Cys Prx BAS1 chloroplastic


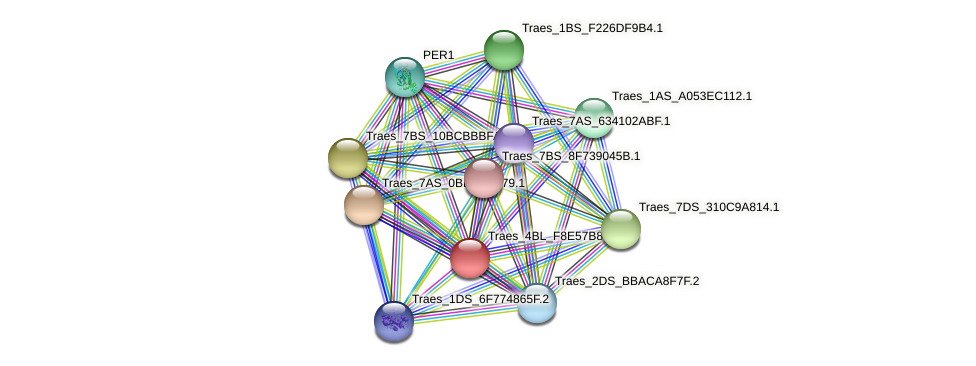


Ssp 7413: EMS48257.1 IN2-1 Protein IN2-1-like protein B; interacting partners other than uncharacterized proteins: GPX (glutathione peroxidase), 1-Cys Prx PER1, proteins from GST superfamily


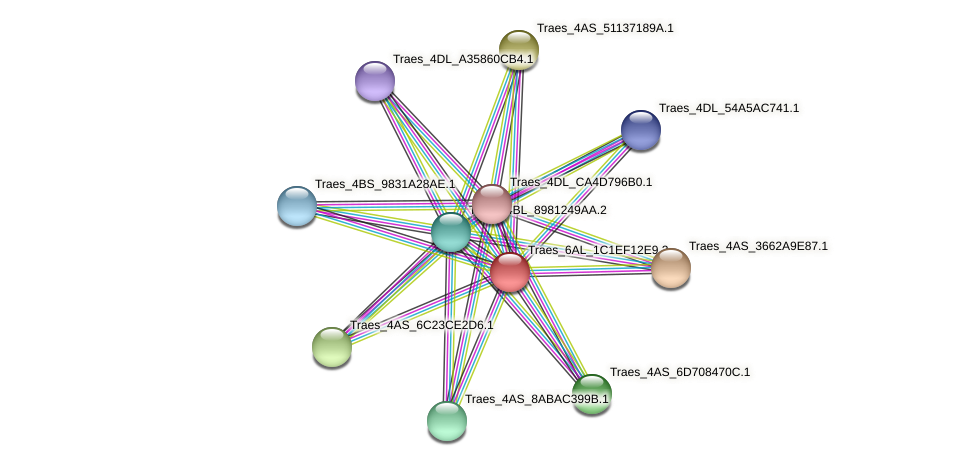


Ssp 7413: XP_020150997.1 nuclear cap-binding protein; interacting partners other than uncharacterized proteins: MIF4-G containing protein, core component of spliceosomal U1, U2, U4, U5 snRNPs


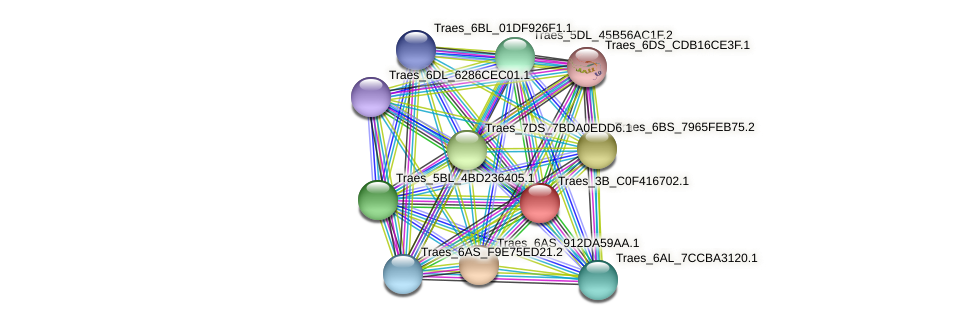


Ssp 7608: XP_037414566.1 phosphoglycerate mutase; interacting partners other than uncharacterized proteins: Phosphoglycerate kinase (PGK)


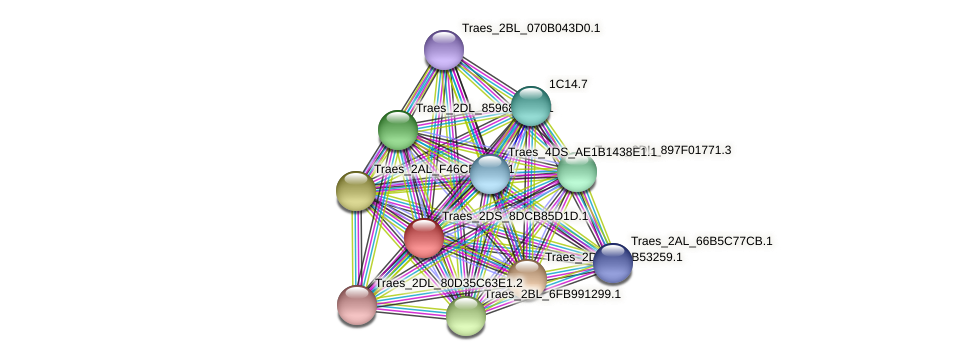


Ssp 7608: XP_037476932.1 polyA binding protein; interacting partners other than uncharacterized proteins: Small nuclear ribonucleoprotein, DEAD-box RNA helicase
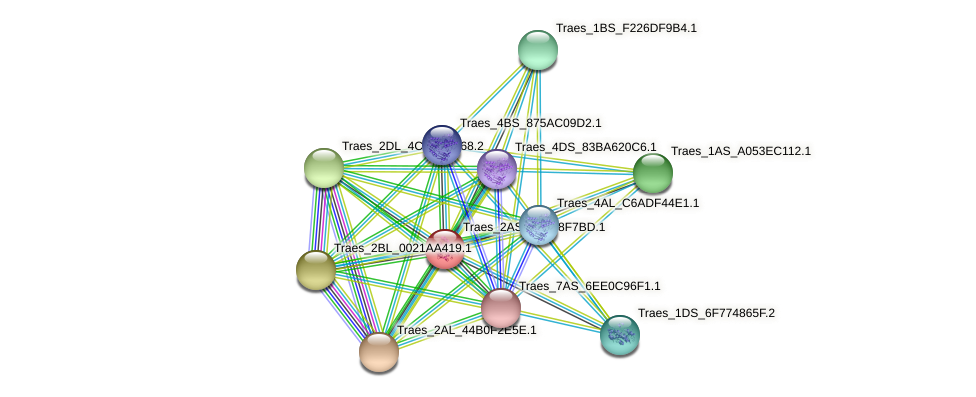


Ssp 8103 and 8106: XP_037475587.1 ascorbate peroxidase; interacting partners other than uncharacterized proteins: Bifunctional dihydrofolate reductase thymidylate synthase, Pyr_redox 2 domain containing protein, GST superfamily


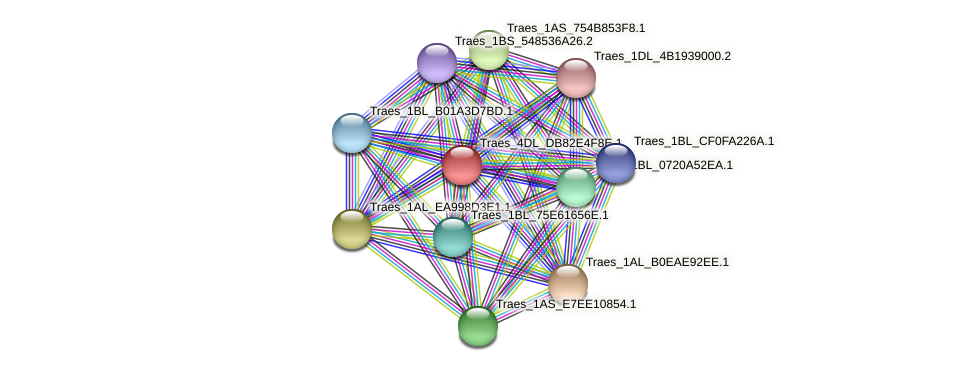


Ssp 8106: XP_037419561.1 proteasome subunit alpha type-6; ; interacting partners other than uncharacterized proteins: Proteasome subunit alpha, beta type


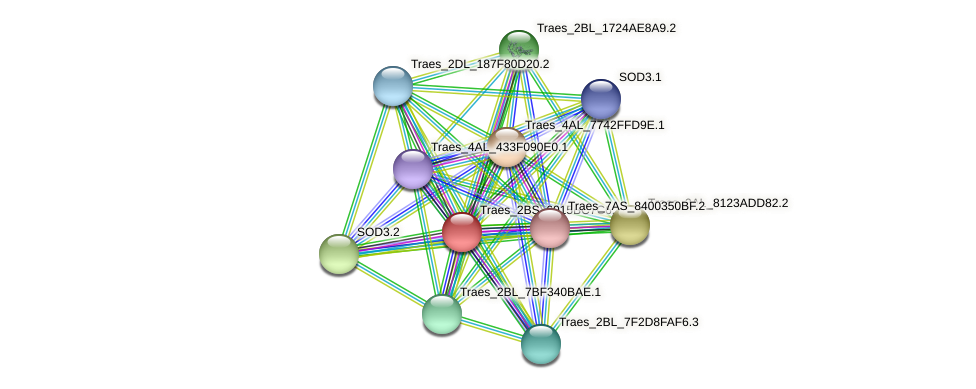


Ssp 8125: AFF27606.1 Cu/Zn-superoxide dismutase; interacting partners other than uncharacterized proteins: SOD3.1, SOD3.2, Hma domain-containing protein


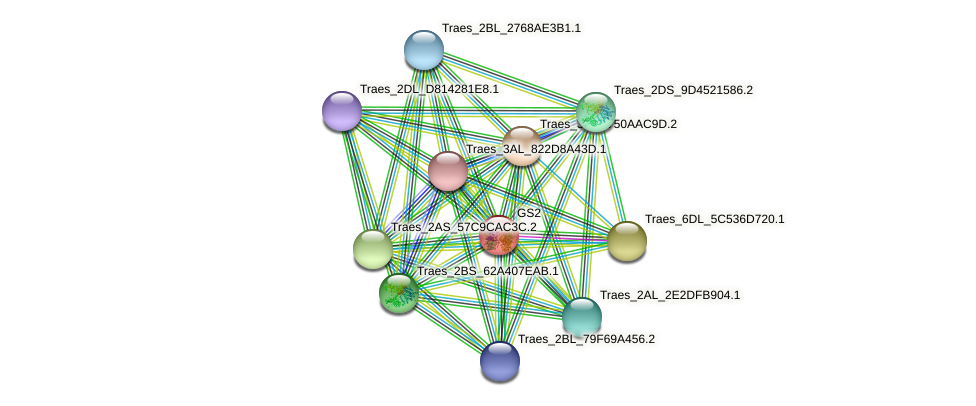


Ssp 8415: KAE8793100.1 plastid glutamine synthetase isoform GS2c, chloroplastic; interacting partners other than uncharacterized proteins: Glutamine synthetase, glutamine amidotransferase


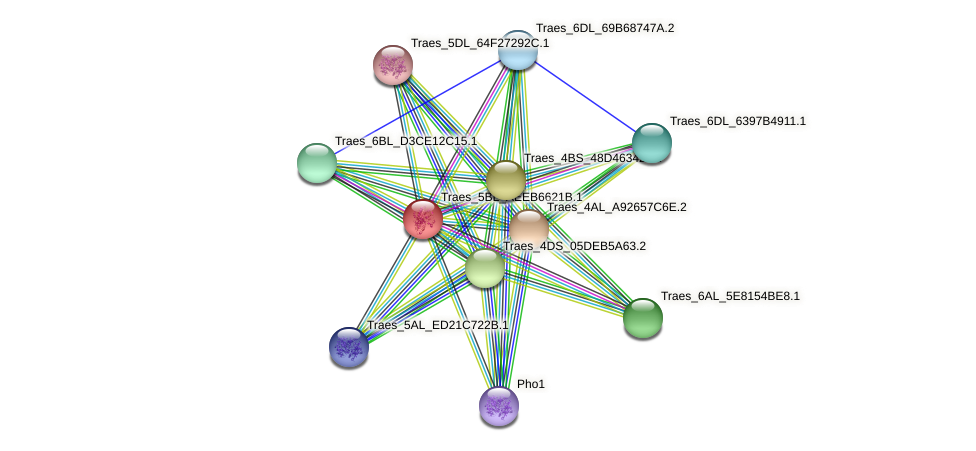


Ssp 8525: XP_020186567.1 UTP--glucose-1-phosphate uridylyltransferase; interacting partners other than uncharacterized proteins: Pho1: alpha-1,4-glucan phosphorylase


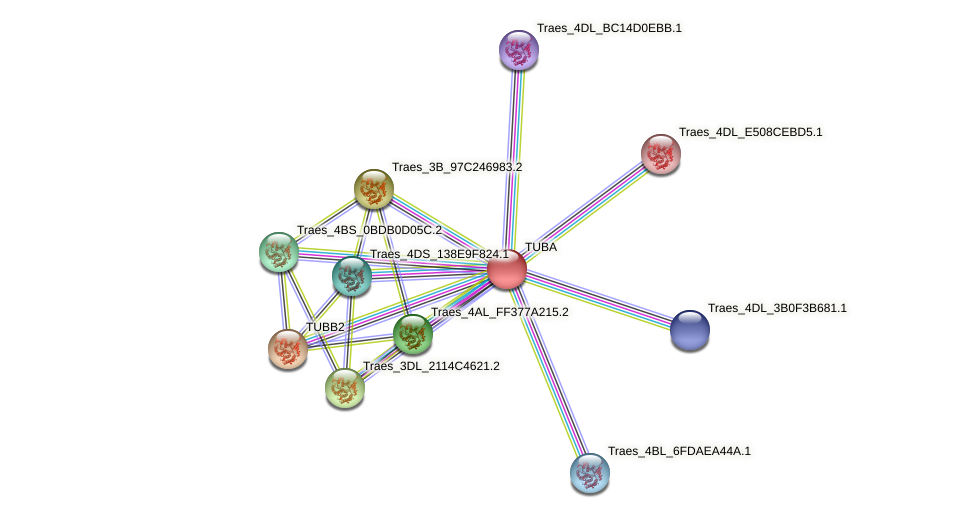


Ssp 8525: Q9ZRB7.1 tubulin alpha chain; interacting partners other than uncharacterized proteins: Tubulin beta, tubulin beta-2


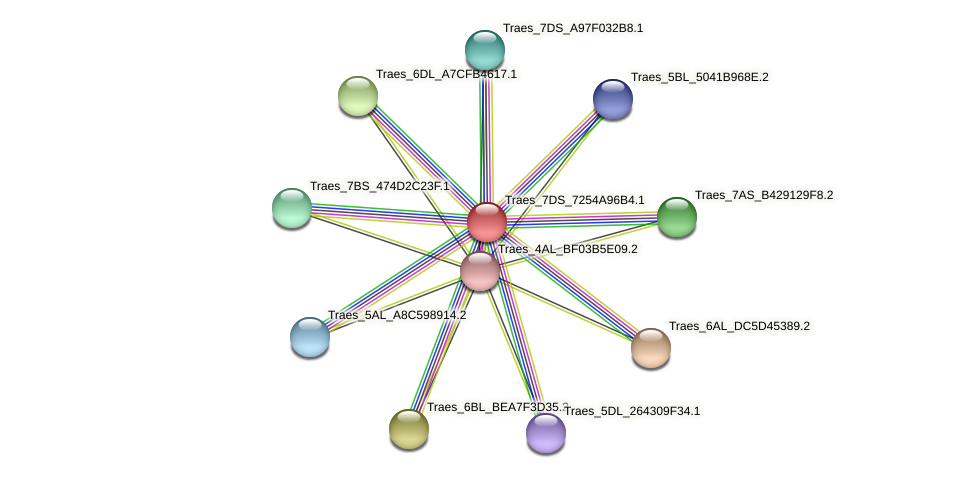


Ssp 8629: XP_020147244.1 RuBisCO large subunit-binding protein subunit beta, chloroplastic; interacting partners other than uncharacterized proteins: GroES chaperonin


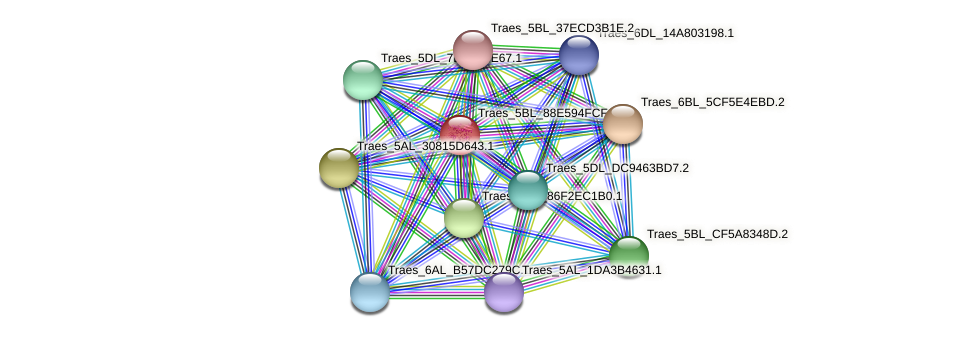


Ssp 8711: XP_037444157.1 heat shock cognate protein 70 Hsc70; interacting partners other than uncharacterized proteins: Hatpase c-domain containing protein


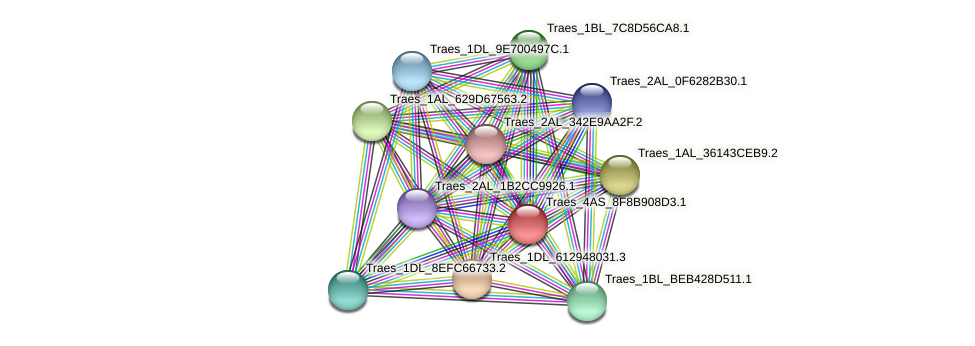


Ssp 9409: AQU14669.1 40S ribosomal protein S2; interacting partners other than uncharacterized proteins: 40S ribosomal protein S8, 60S ribosomal protein L29, ribosomal L4 C domain containing protein


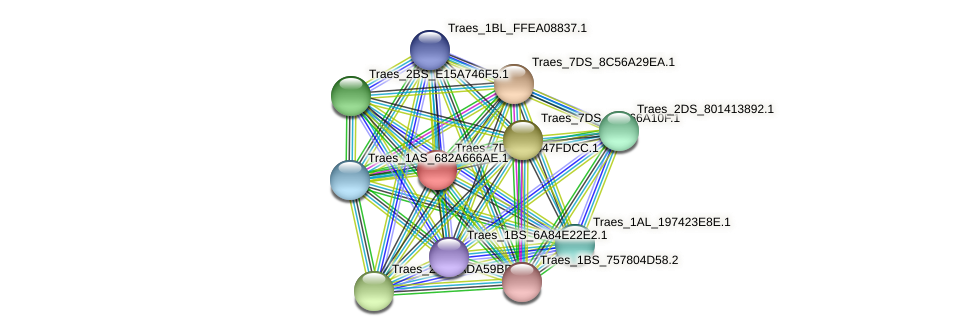


Ssp 9410: XP_020148641.1 fructokinase 2; interacting partners other than uncharacterized proteins: Glucose-6-phosphate isomerase, phosphotransferase


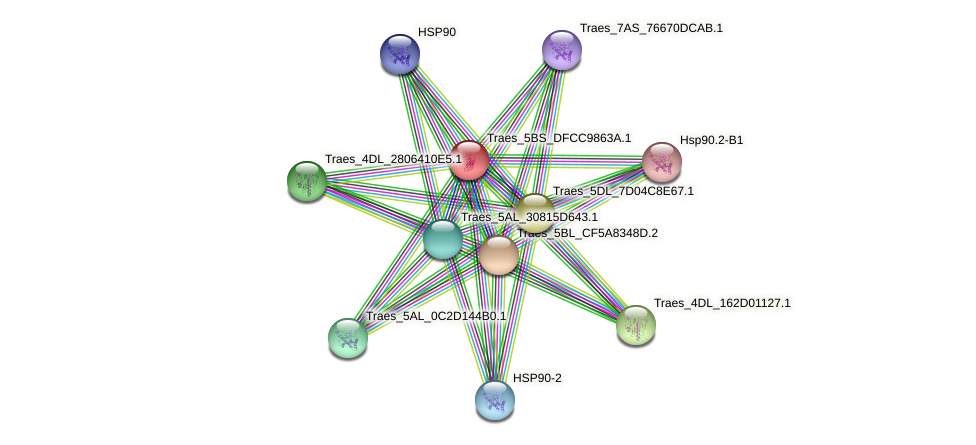


Ssp 9601: XP_037439714.1 Hsc70; interacting partners other than uncharacterized proteins: HSP90, HSP90-2


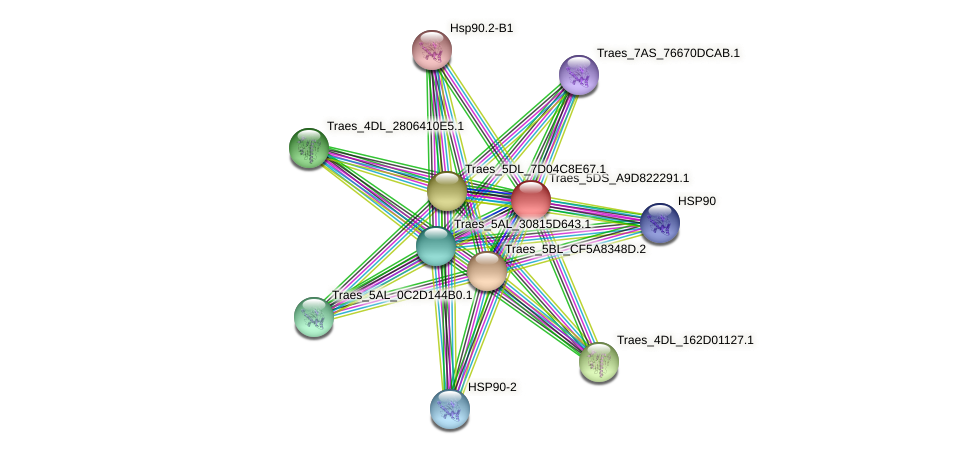


Ssp 9603: VAI28258.1 unnamed protein predicted Hsp70; interacting partners other than uncharacterized proteins: HSP90, HSP90-2
