# Supplementary material for: Proteomic responses of two spring wheat cultivars to the combined water deficit and aphid (Metopolophium dirhodum) treatments
Source: Front Plant Sci. 2022 Nov 14;13:1005755. doi: 10.3389/fpls.2022.1005755 (PMC9704420; doi:10.3389/fpls.2022.1005755)
Supplement: Supplementary Figure 5 — Cluster analysis of the 113 DAPs and six morphophysiological characteristics (fresh above-ground biomass, leaf length, WSD, Ψπ, Ψπ100, Fv/Fm). The data were normalized using Z-score transformation and cluster analysis was performed in PermutMatrix software using Euclidean distances and Ward´s minimum criteria as algorithms for clusterogram construction. [file Presentation_1.pptx]

## Slide 1
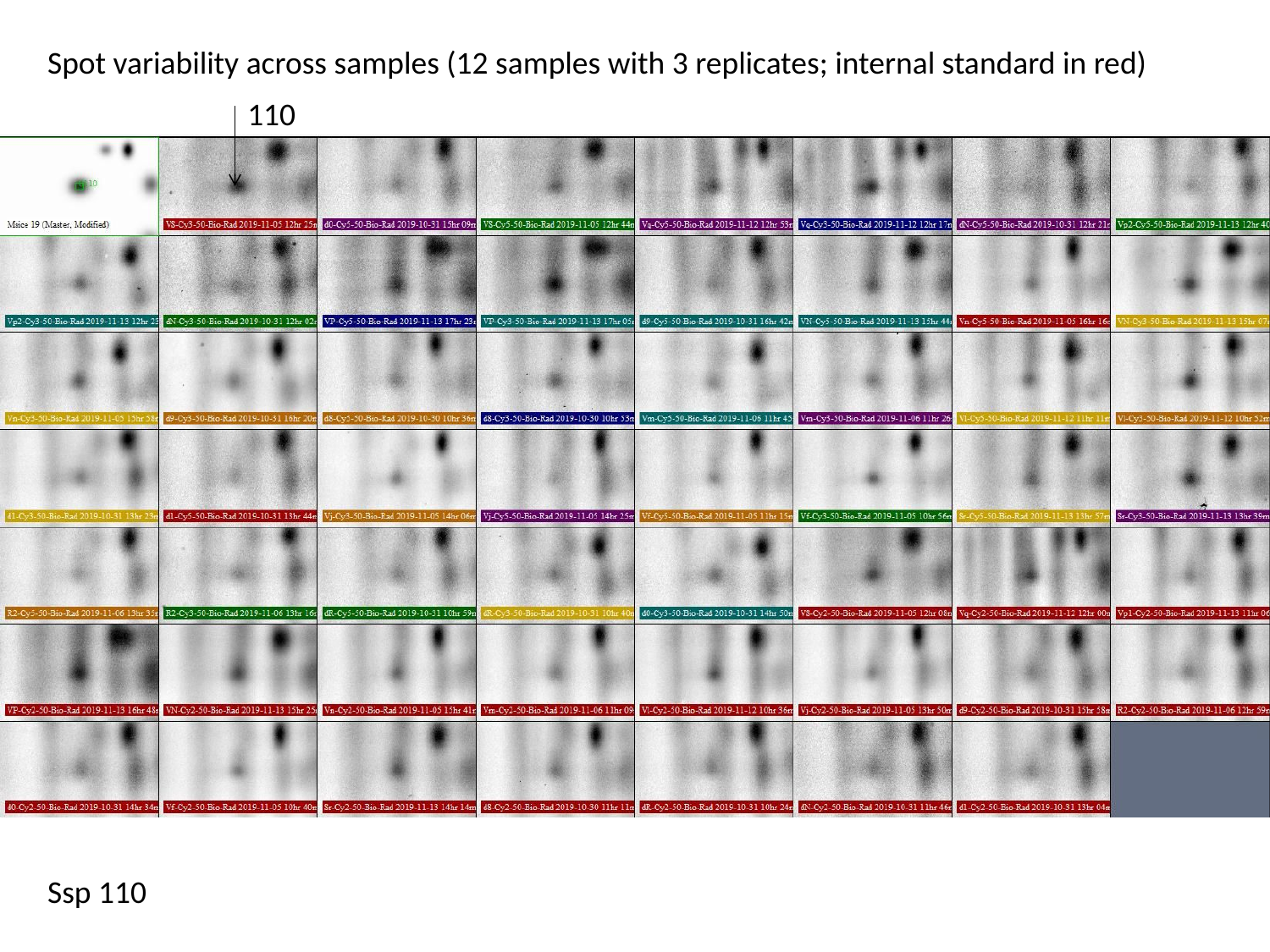

Spot variability across samples (12 samples with 3 replicates; internal standard in red)
110
Ssp 110

## Slide 2
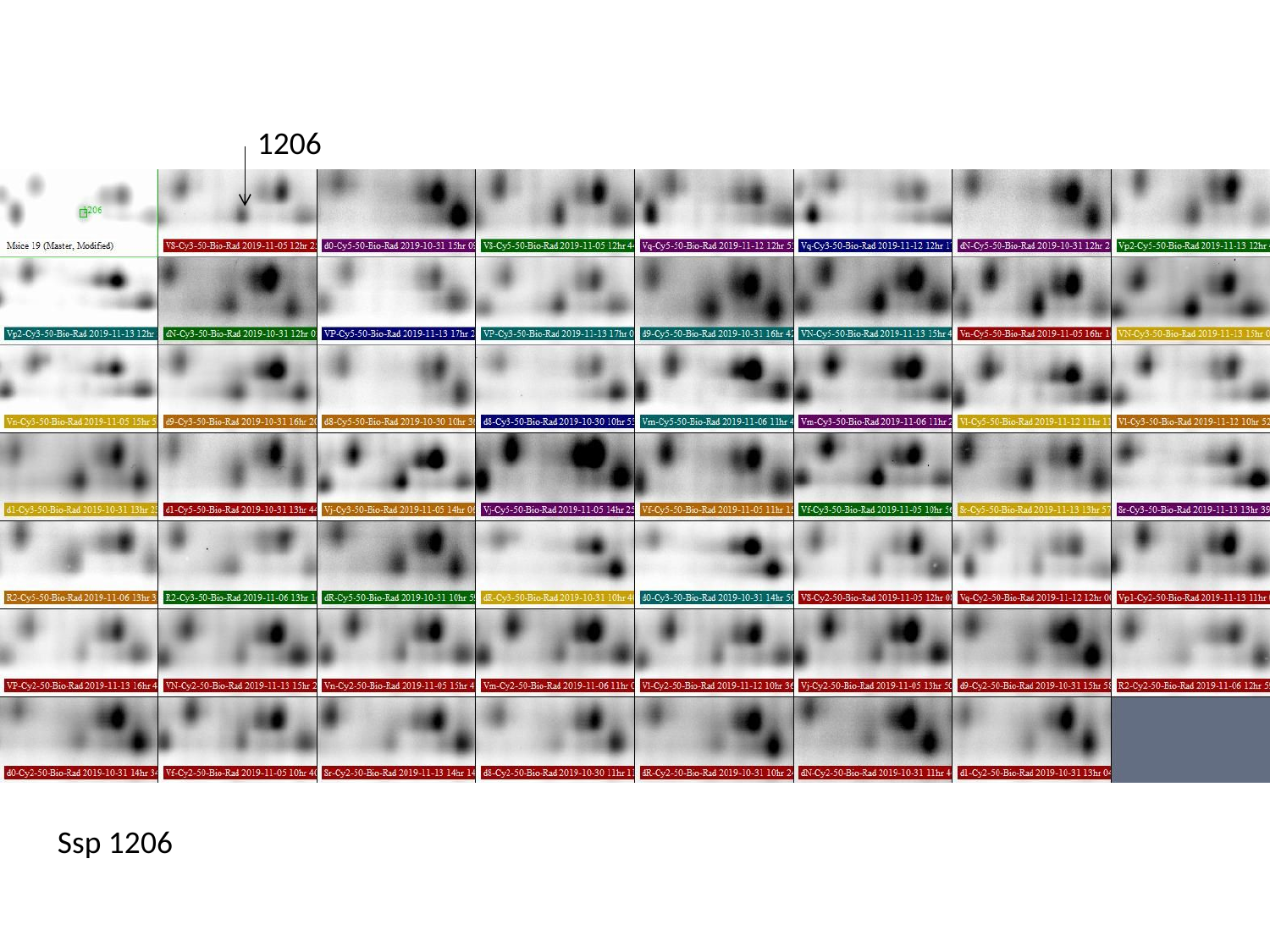

1206
Ssp 1206

## Slide 3
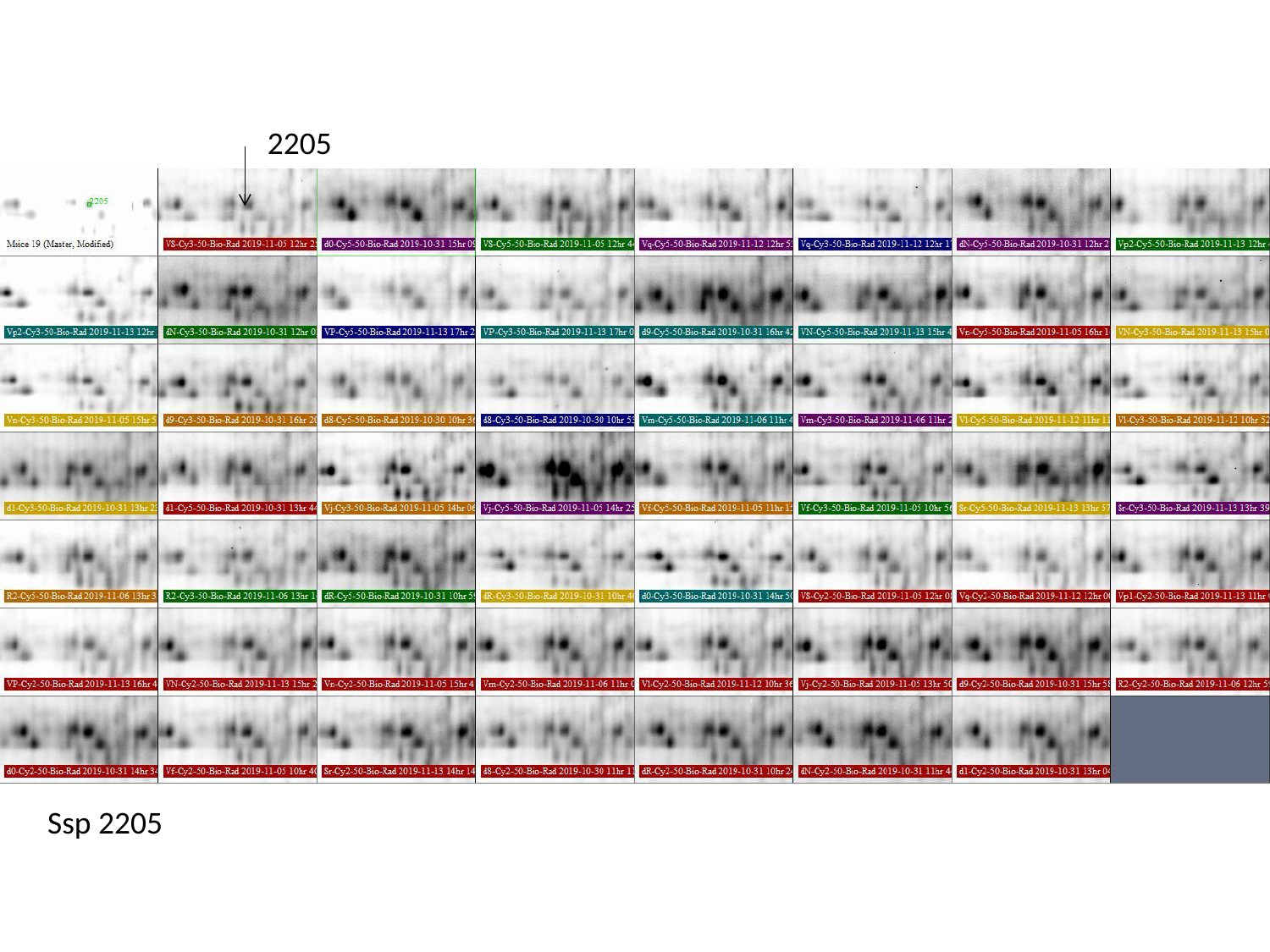

2205
Ssp 2205

## Slide 4
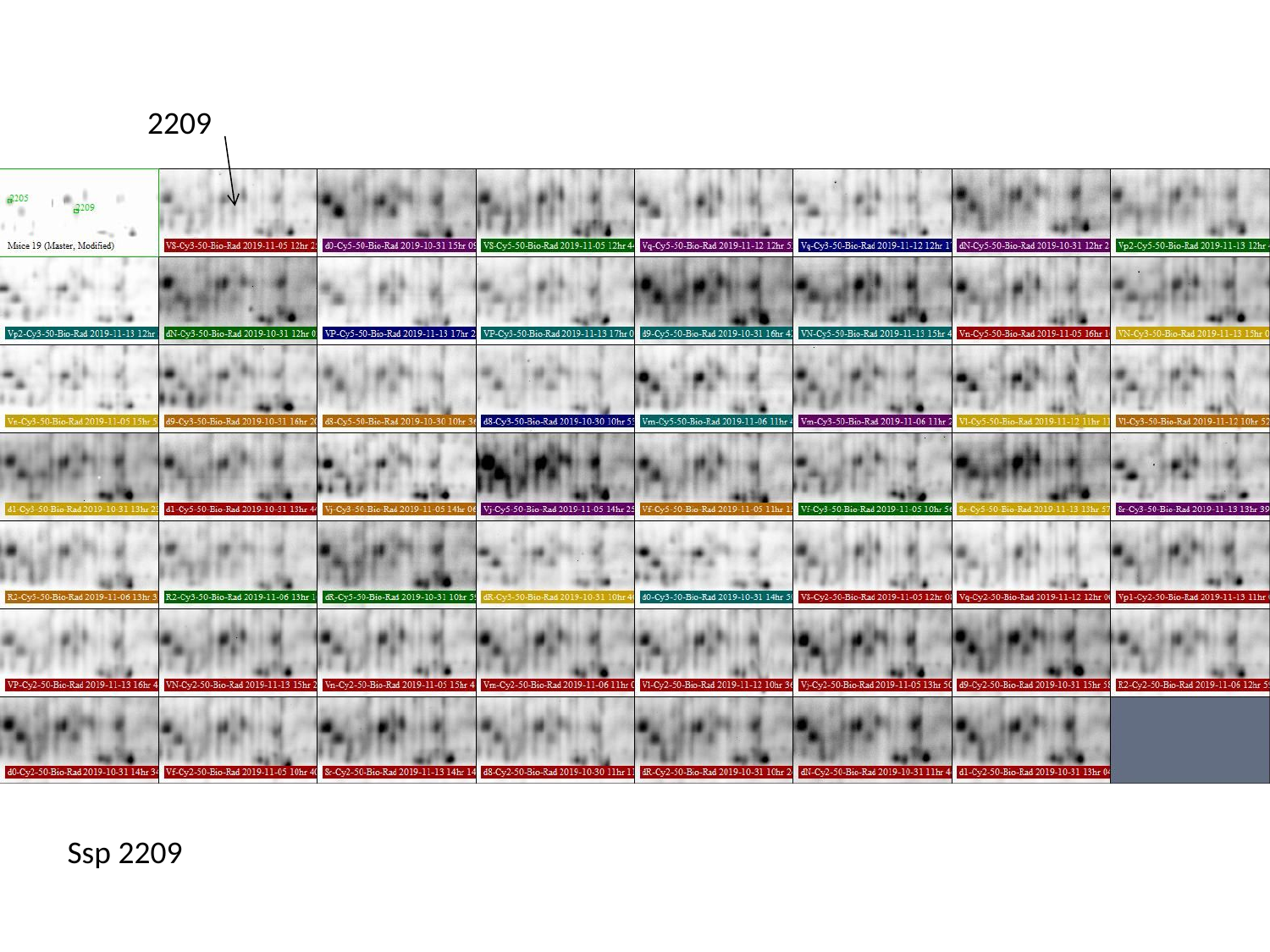

2209
Ssp 2209

## Slide 5
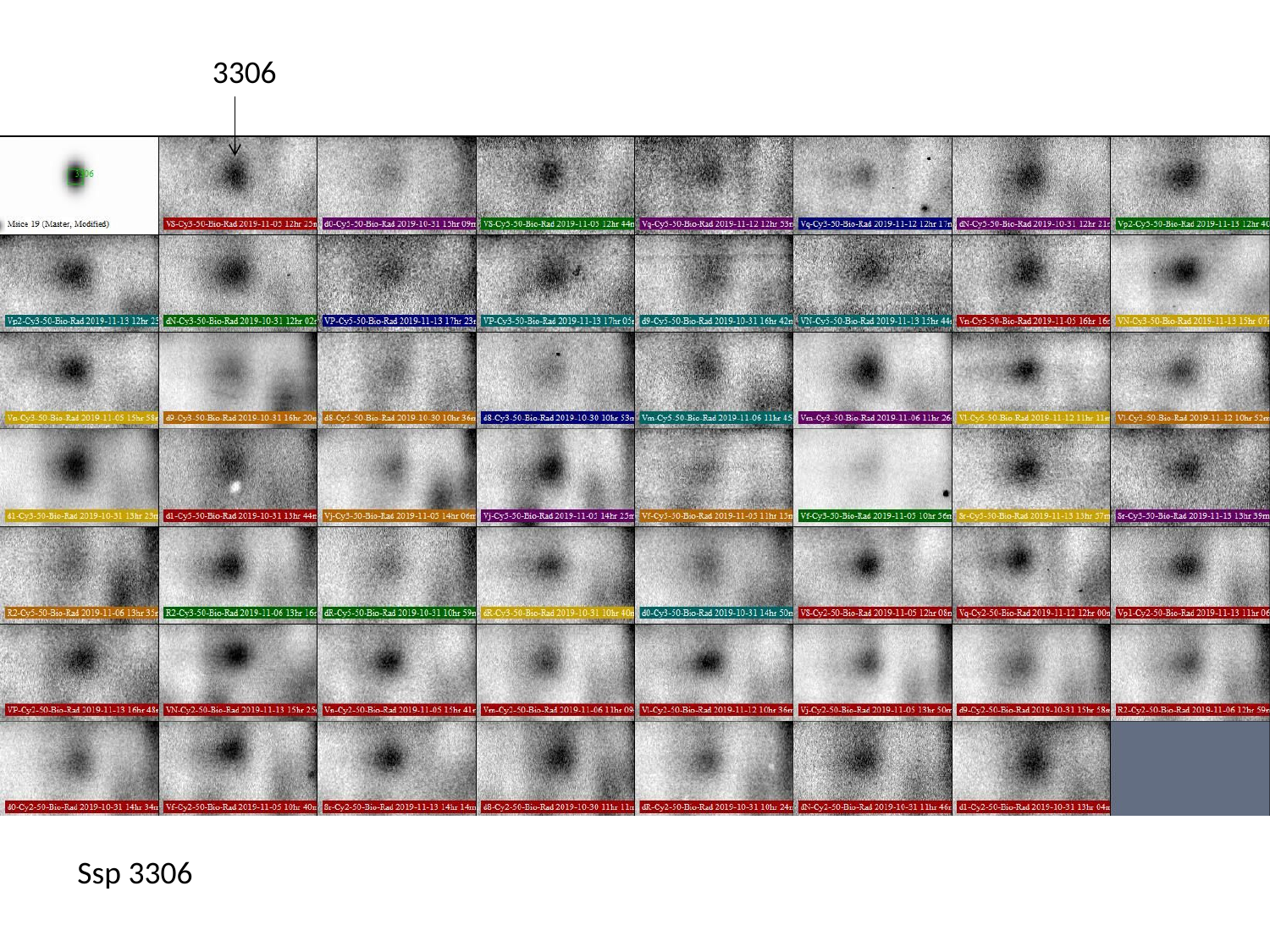

3306
Ssp 3306

## Slide 6
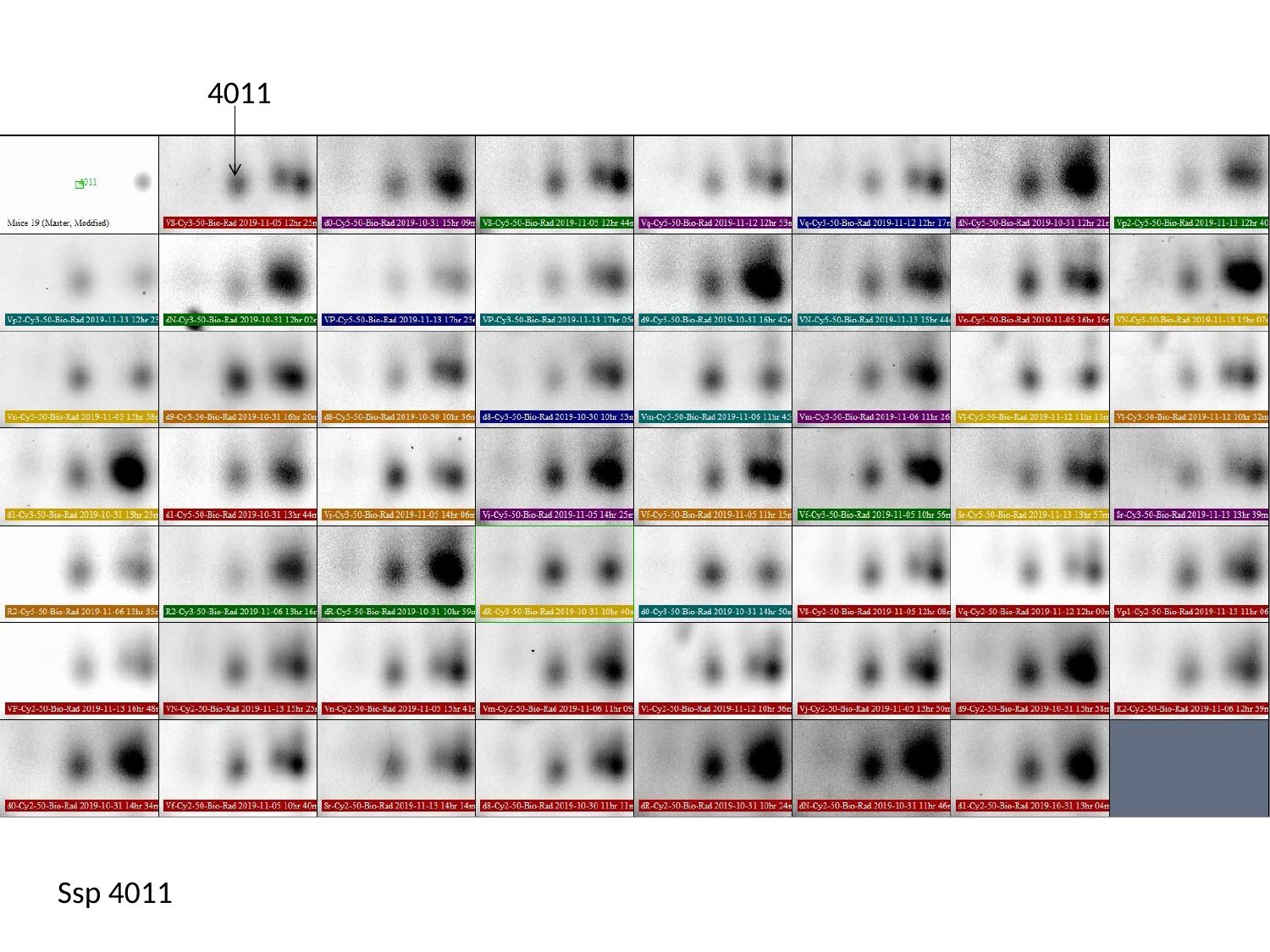

4011
Ssp 4011

## Slide 7
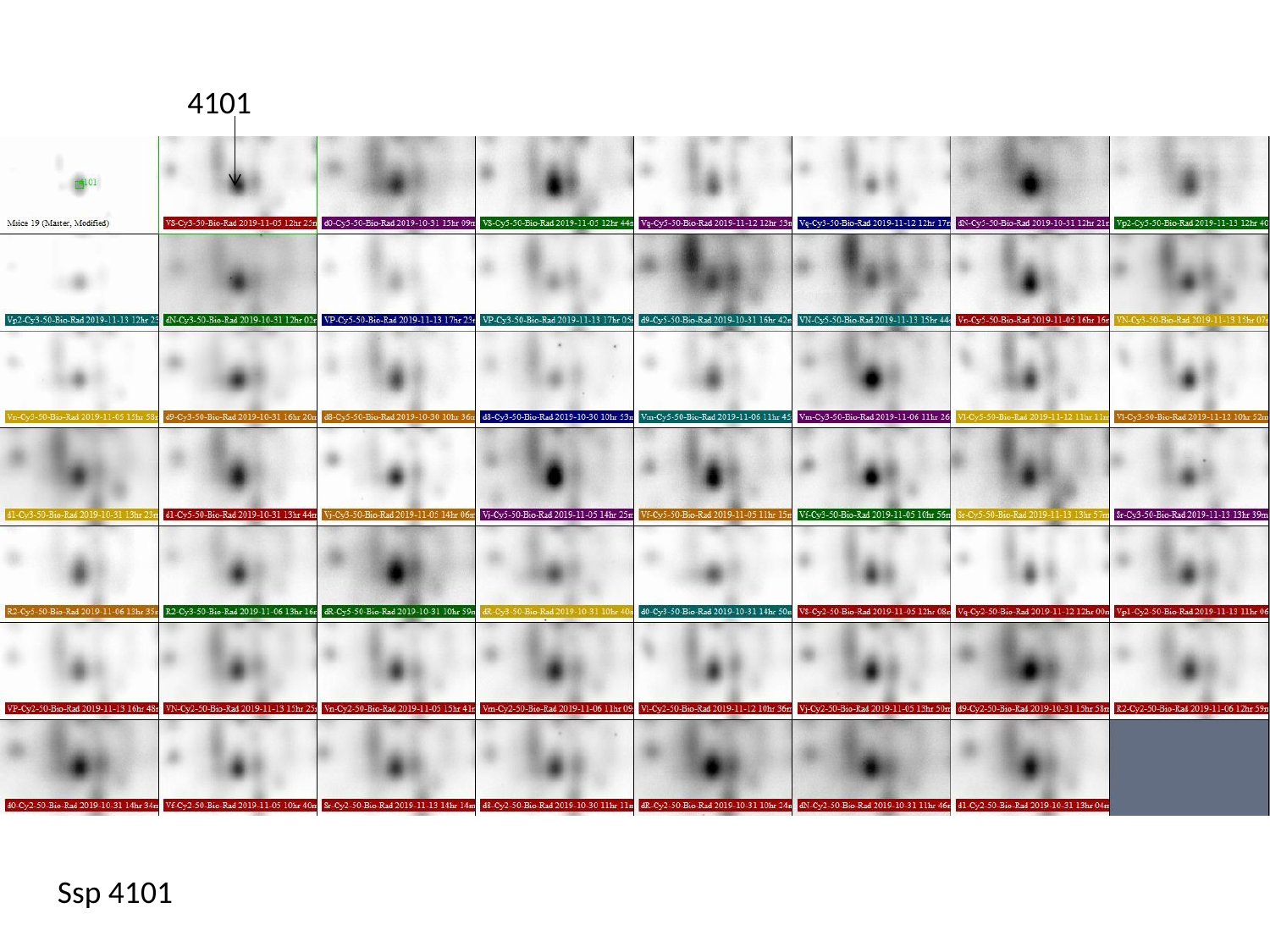

4101
Ssp 4101

## Slide 8
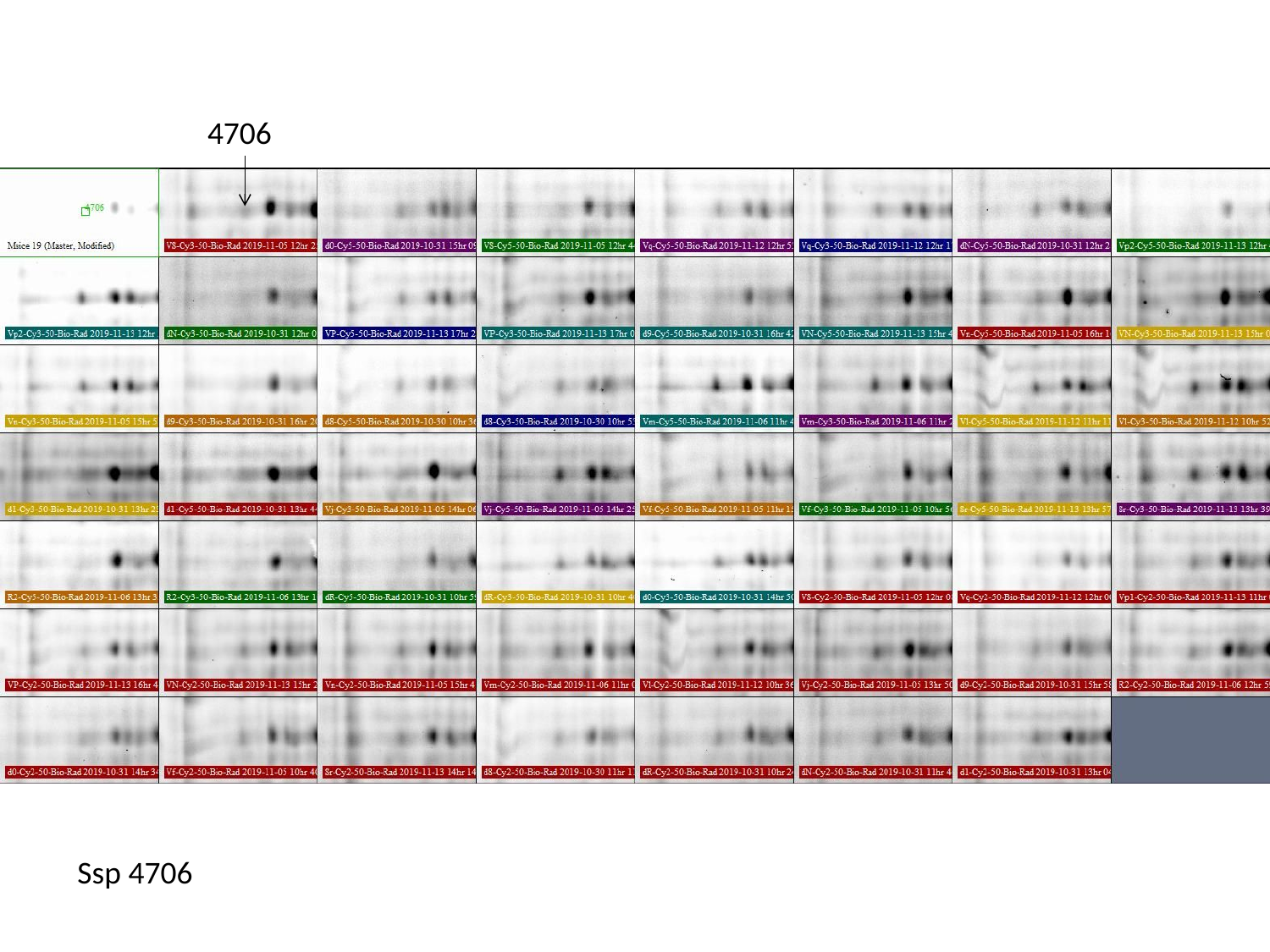

4706
Ssp 4706

## Slide 9
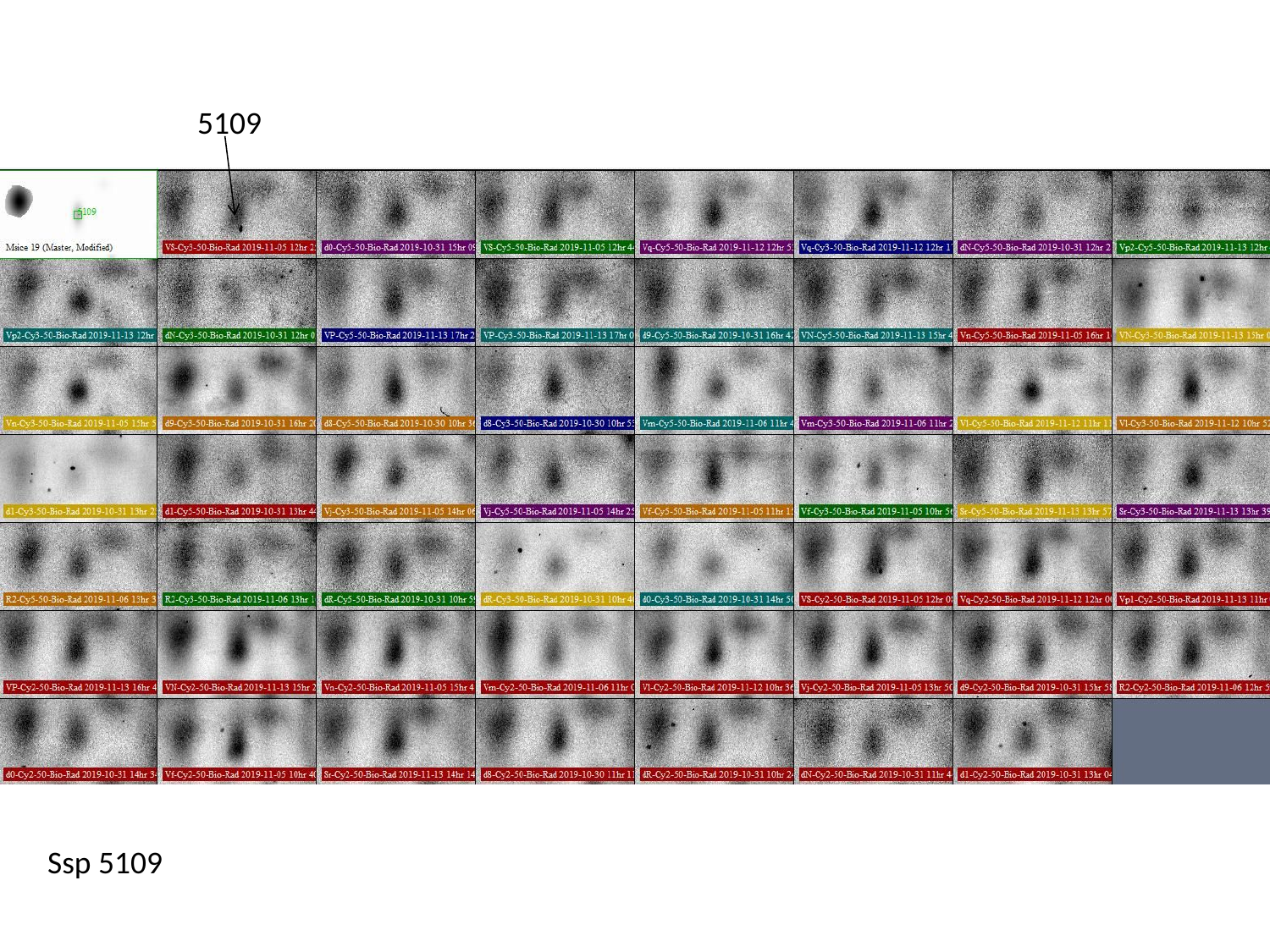

5109
Ssp 5109

## Slide 10
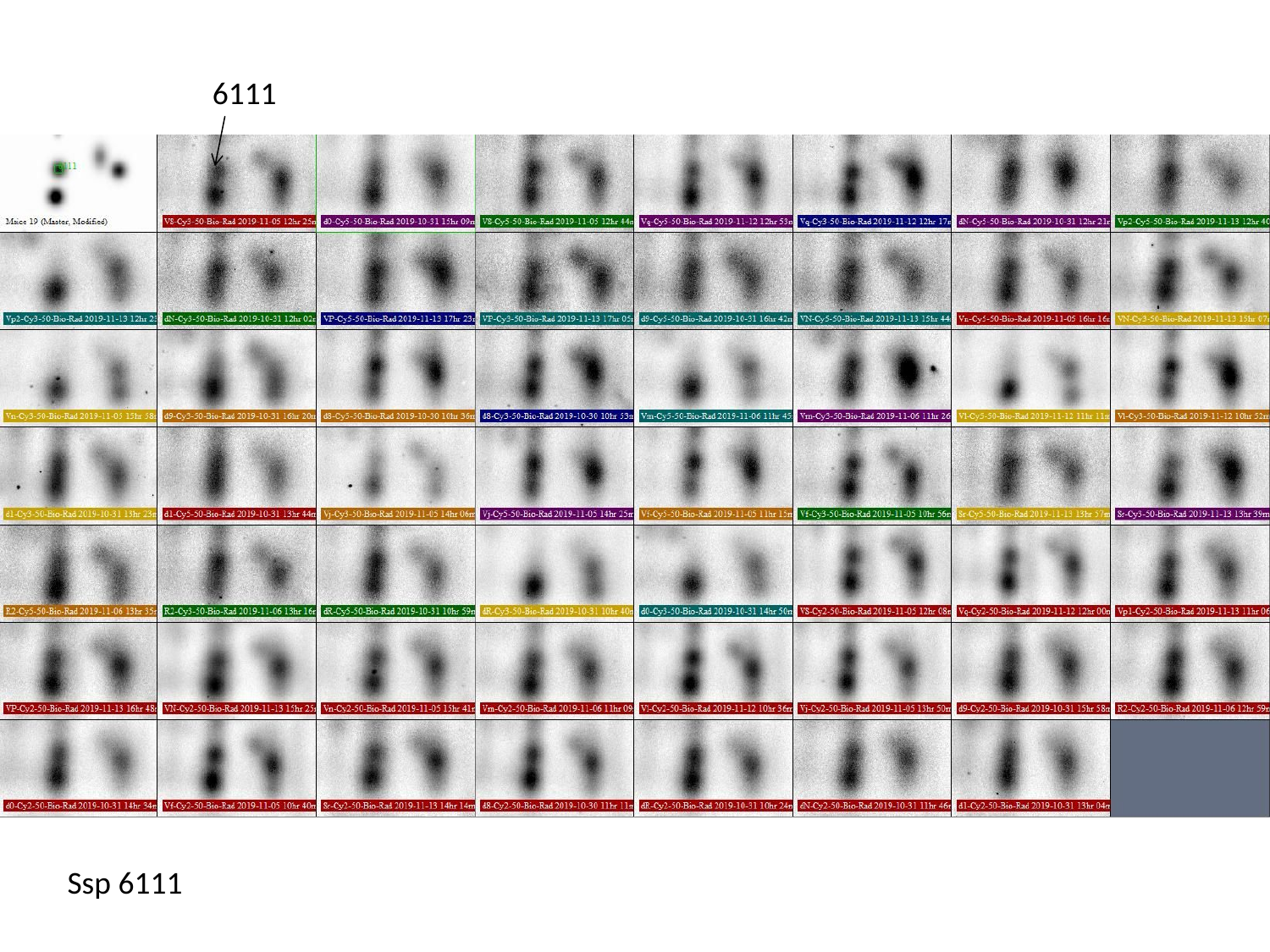

6111
Ssp 6111

## Slide 11
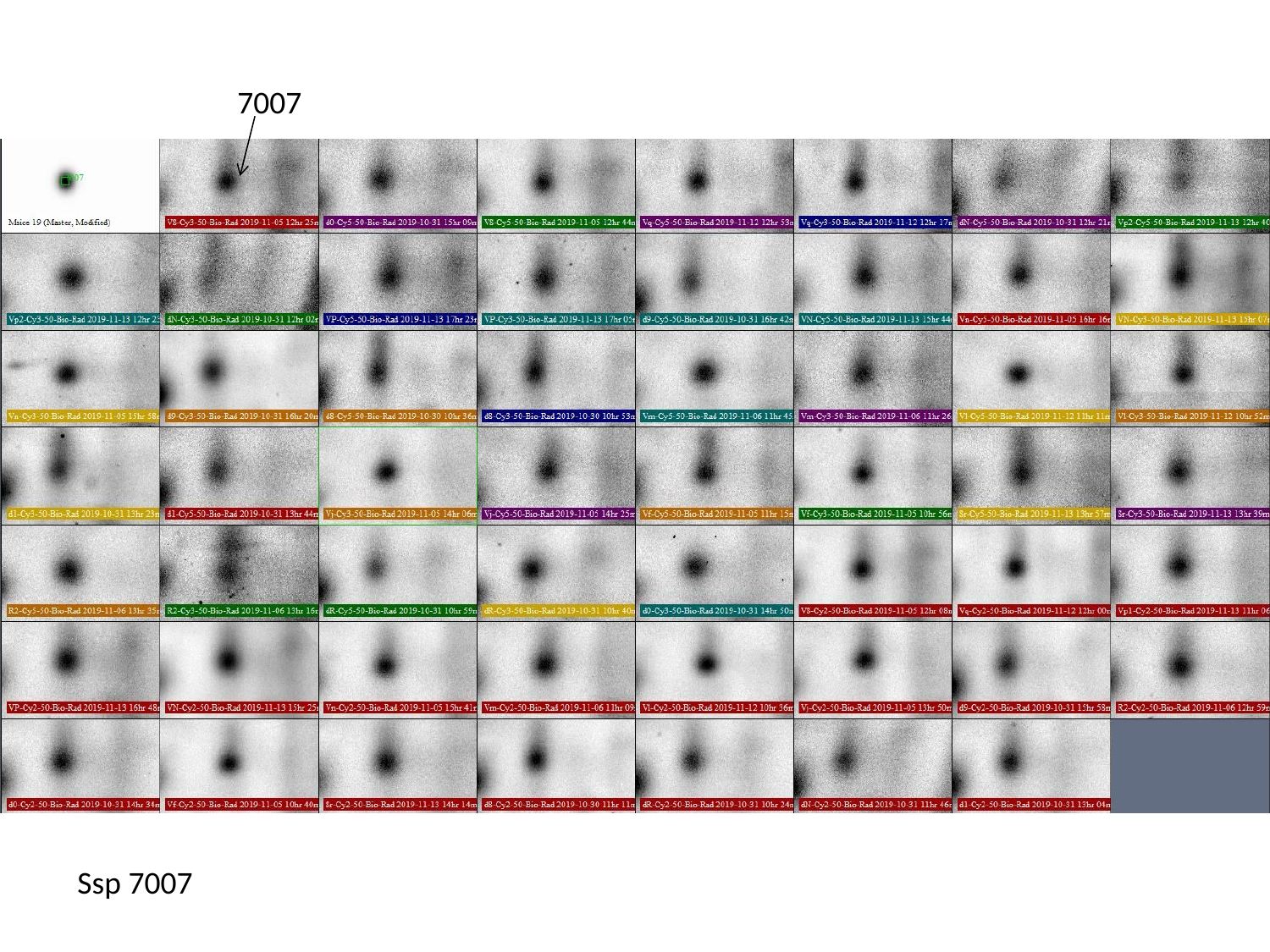

7007
Ssp 7007

## Slide 12
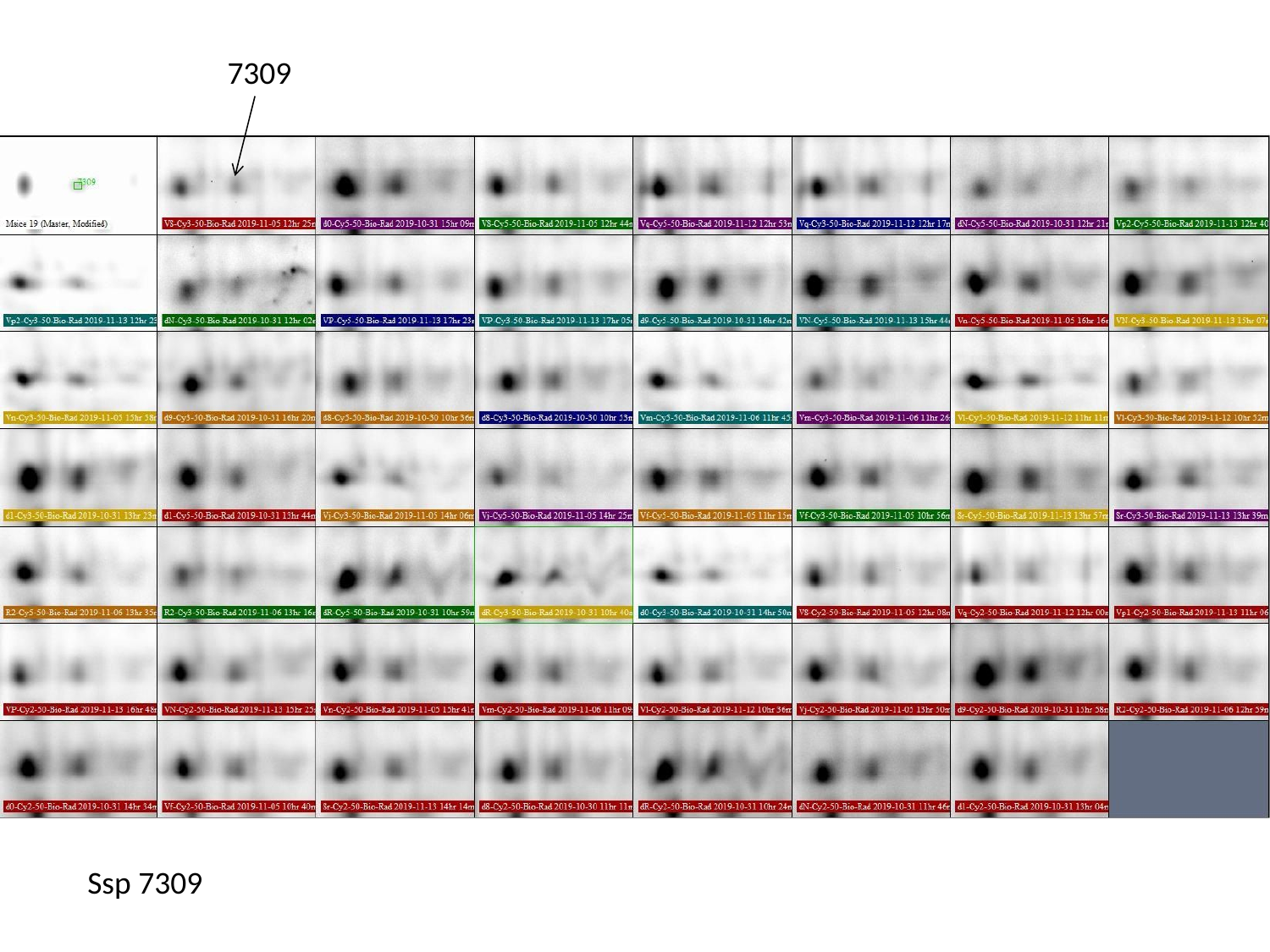

7309
Ssp 7309

## Slide 13
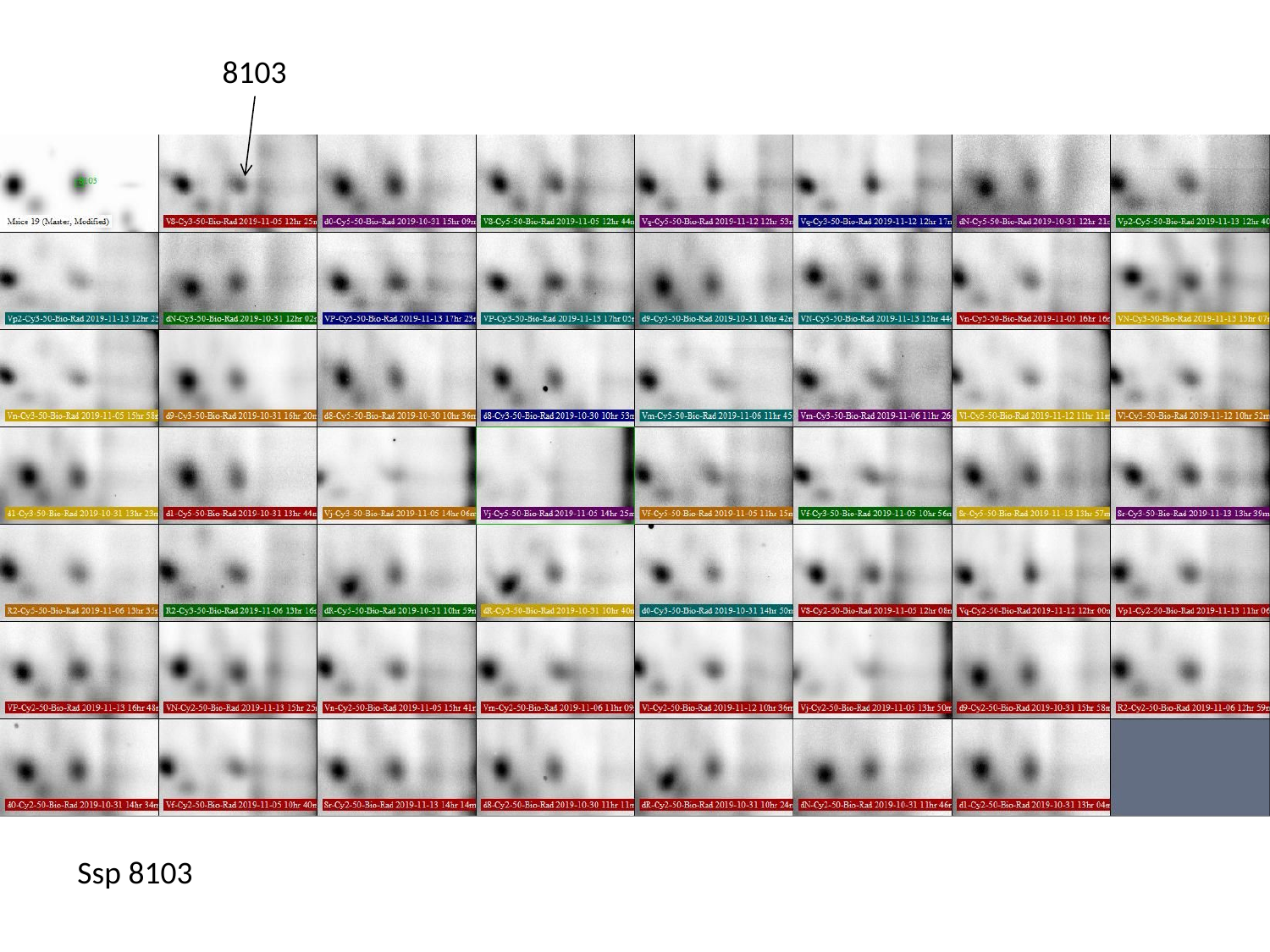

8103
Ssp 8103
