# Supplementary material for: Proteomic responses of two spring wheat cultivars to the combined water deficit and aphid (Metopolophium dirhodum) treatments
Source: Front Plant Sci. 2022 Nov 14;13:1005755. doi: 10.3389/fpls.2022.1005755 (PMC9704420; doi:10.3389/fpls.2022.1005755)
Supplement: Supplementary Table 1 — Estimated mean values for leaf morpho-physiological traits of spring wheat seedlings under the variable conditions of water supply (soil water capacity, SWC [%]) and aphid infestation. SE denotes standard error. The same letters within the columns indicate values that were not significantly different from each other based on a paired bootstrap test. Data from Saska et al. (2022). [file Table_1.docx]

**Supplementary Table S1.** Estimated mean values for leaf morpho-physiological traits of spring wheat seedlings under the variable conditions of water supply (soil water capacity, SWC [%]) and aphid infestation. SE denotes standard error. The same letters within the columns indicate values that were not significantly different from each other based on a paired bootstrap test. Data from Saska et al. (2022).

| Cultivar | SWC | No aphids | | Aphids | |
| --- | --- | --- | --- | --- | --- |
|  |  | Mean ± SE | | Mean ± SE | |
| Aboveground fresh biomass [g] | | |  |  |  |
| Quintus | 70 | 1.561 ± 0.0548 a | | 1.637 ± 0.0529 a | |
|  | 50 | 1.291 ± 0.0548 b | | 1.293 ± 0.0529 b | |
|  | 40 | 1.018 ± 0.0548 c | | 1.175 ± 0.0538 c | |
| Septima | 70 | 1.576 ± 0.0542 a | | 1.440 ± 0.0538 a | |
|  | 50 | 1.239 ± 0.0554 b | | 1.256 ± 0.0533 b | |
|  | 40 | 0.956 ± 0.0542 c | | 1.023 ± 0.0542 c | |
| $\Psi_{\pi100}$ [MPa] |  |  |  |  |  |
| Quintus | 70 | -1.05 ± 0.0466 b | | -1.15 ± 0.0467 c | |
|  | 50 | -1.19 ± 0.0469 c | | -1.26 ± 0.0467 d | |
|  | 40 | -1.32 ± 0.0467 e | | -1.36 ± 0.0468 e | |
| Septima | 70 | -0.97 ± 0.0468 a | | -1.04 ± 0.0468 b | |
|  | 50 | -1.07 ± 0.0467 b | | -1.23 ± 0.0467 d | |
|  | 40 | -1.24 ± 0.0470 d | | -1.28 ± 0.0468 d | |
