# Supplementary material for: Proteomic responses of two spring wheat cultivars to the combined water deficit and aphid (Metopolophium dirhodum) treatments
Source: Front Plant Sci. 2022 Nov 14;13:1005755. doi: 10.3389/fpls.2022.1005755 (PMC9704420; doi:10.3389/fpls.2022.1005755)
Supplement: Supplementary Table 2 — Life table parameters (means ± SE) of Metopolophium dirhodum reared on two cultivars (Quintus and Septima) of Triticum aestivum. SE were estimated with bootstrapping (100,000 resamplings). λ - finite rate of increase, r - intrinsic rate of increase, R 0 – net reproductive rate; T – generation time. The same letters within the columns indicate values that were not significantly different from each other based on a paired bootstrap test. Data from Saska et al. (2022). [file Table_2.docx]

**Supplementary Table S2.** Life table parameters (means ± SE) of *Metopolophium dirhodum* reared on two cultivars (Quintus and Septima) of *Triticum aestivum*. SE were estimated with bootstrapping (100,000 resamplings). *λ* - finite rate of increase, *r* - intrinsic rate of increase, *R*_0_ – net reproductive rate; T – generation time. The same letters within the columns indicate values that were not significantly different from each other based on a paired bootstrap test. Data from Saska et al. (2022).

| Parameter | SWC | Duration of development (d) | Fecundity (offspring female^−1^) | *λ* (d^−1^) | *r* (d^−1^) | *R*_0_ (offspring individual^−1^) | *T* (d) |
| --- | --- | --- | --- | --- | --- | --- | --- |
| Quintus | 70 | 7.83 ± 0.10 a | 41.8 ± 1.4 b | 1.303 ± 0.005 c | 0.264 ± 0.004 c | 36.66 ± 1.94 b | 13.63 ± 0.15 b |
|  | 50 | 8.65 ± 0.06 c | 31.7 ± 1.8 a | 1.267 ± 0.004 a | 0.236 ± 0.003 a | 28.78 ± 1.87 a | 14.21 ± 0.19 c |
|  | 40 | 8.07 ± 0.05 b | 31.9 ± 1.8 a | 1.282 ± 0.004 b | 0.248 ± 0.003 b | 29.62 ± 1.82 a | 13.64 ± 0.14 b |
| Septima | 70 | 7.89 ± 0.07 a | 42.1 ± 1.9 b | 1.296 ± 0.004 c | 0.259 ± 0.003 c | 38.44 ± 2.16 b | 14.09 ± 0.14 c |
|  | 50 | 8.48 ± 0.07 c | 31.8 ± 1.7 a | 1.265 ± 0.004 a | 0.235 ± 0.003 a | 28.13 ± 1.88 a | 14.18 ± 0.18 c |
|  | 40 | 7.70 ± 0.07 a | 29.9 ± 1.5 a | 1.290 ± 0.005 bc | 0.255 ± 0.004 bc | 26.28 ± 1.70 a | 12.84 ± 0.16 a |
