# Supplementary material for: Proteomic responses of two spring wheat cultivars to the combined water deficit and aphid (Metopolophium dirhodum) treatments
Source: Front Plant Sci. 2022 Nov 14;13:1005755. doi: 10.3389/fpls.2022.1005755 (PMC9704420; doi:10.3389/fpls.2022.1005755)
Supplement: Supplementary Table 3 — A list of the identified protein spots revealing differential abundance, i.e., min. 1.5 fold change at 0.05 level determined by Student’s t-test, in at least one out of 24 biologically relevant sample ratios. SSP – protein spot number; accession n. according to NCBI protein database (access: 23rd April, 2021; taxonomy: Triticeae: NCBI txid147389_20210423 (518563 sequences; 220219273 residues)), MS score – MASCOT Score, Nr of matches – number of unique peptides matched to the given sequence, pI/MW theor. was calculated from the downloaded NCBI protein sequence using “Compute pI/MW” tool in Expasy database (www.web.expasy.org). Significant ratio: 1: 70 (S/Q); 2: 50 (S/Q); 3: 40 (S/Q); 4: S (50/70); 5: S (40/70); 6: S (40/50); 7: Q (50/70); 8: Q (40/70); 9: Q (40/50); 10: 70 (Qm/Q); 11: 50 (Qm/Q); 12: 40 (Qm/Q); 13: 70 (Sm/S); 14: 50 (Sm/S); 15: 40 (Sm/S); 16: 70 (Sm/Qm); 17: 50 (Sm/Qm); 18: 40 (Sm/Qm); 19: Sm (50/70); 20: Sm (40/50); 21: Sm (40/70); 22: Qm (50/70); 23: Qm (40/50); 24: Qm (40/70) where Q means Quintus, S means Septima, Qm means aphid-treated Quintus, Sm means aphid-treated Septima, 40 means 40% SWC, 50 means 50% SWC, 70 means 70% SWC; ↑ means an increased rel. abundance while ↓ means decreased rel. abundance. [file Table_3.docx]

Supplementary Table S3. A list of the identified protein spots revealing differential abundance, i.e., min. 1.5 fold change at 0.05 level determined by Student T-test, in at least one out of 24 biologically relevant sample ratios. SSP – protein spot number; accession n. according to NCBI protein database (access: 23rd April, 2021; taxonomy: Triticeae: NCBI txid147389_20210423 (518563 sequences; 220219273 residues)), MS score – MASCOT Score, Nr of matches – number of unique peptides matched to the given sequence, pI/MW theor. was calculated from the downloaded NCBI protein sequence using “Compute pI/MW“ tool in Expasy database ([www.web.expasy.org](http://www.web.expasy.org)).

Significant ratio: 1: 70 (S/Q); 2: 50 (S/Q); 3: 40 (S/Q); 4: S (50/70); 5: S (40/70); 6: S (40/50); 7: Q (50/70); 8: Q (40/70); 9: Q (40/50); 10: 70 (Qm/Q); 11: 50 (Qm/Q); 12: 40 (Qm/Q); 13: 70 (Sm/S); 14: 50 (Sm/S); 15: 40 (Sm/S); 16: 70 (Sm/Qm); 17: 50 (Sm/Qm); 18: 40 (Sm/Qm); 19: Sm (50/70); 20: Sm (40/50); 21: Sm (40/70); 22: Qm (50/70); 23: Qm (40/50); 24: Qm (40/70) where Q means Quintus, S means Septima, Qm means aphid-treated Quintus, Sm means aphid-treated Septima, 40 means 40% SWC, 50 means 50% SWC, 70 means 70% SWC; ↑ means an increased rel. abundance while ↓ means decreased rel. abundance.

| SSP | Accession number (organism) | Protein name | | MS score | Nr of matches | pI/MW theor. | pI/MW exp. | Significant ratio | | | |
| --- | --- | --- | --- | --- | --- | --- | --- | --- | --- | --- | --- |
| Amino acid metabolism | | | | |  |  |  |  | | | |
| 4706 | XP_037439131.1 (*Triticum dicoccoides*) | 5-methyltetrahydropteroyltriglutamate--homocysteine methyltransferase 1-like | | 426 | 22 | 5.74/84.59 | 6.5/84.6 | 1, 2, 3, 14, 15, 16, 17, 18, 20, 22 ↓ | | | |
| 5302 | EMS64657.1  (*Triticum urartu*) | putative diaminopimelate decarboxylase, chloroplastic | | 73 | 3 | 8.69/77.63 | 6.79/49.19 | 3 ↑ 8, 9, 11 ↓ | | | |
|  |  |  | |  |  |  |  |  | | | |
| 5705 | KAE8786474.1 (*Hordeum vulgare*) | methionine synthase 1 | | 140 | 9 | 5.74/84.56 | 6.71/58.95 | 3, 5, 13, 16 ↑ | | | |
| 6613 | XP_037479697.1 (*Triticum dicoccoides*) | D-3-phosphoglycerate dehydrogenase 1, chloroplastic-like | | 579 | 20 | 6.59/63.85 | 6.95/60.7 | 1, 8, 15, 20, 21 ↓ | | | |
| 7004 | KAE8774345.1 (*Hordeum vulgare*) | reactive intermediate deaminase A, chloroplastic | | 345 | 10 | 8.71/18.67 | 7.29/11.64 | 7 ↑ 11 ↓ | | | |
| Nitrogen metabolism | | |  |  |  |  |  |  | | | |
| 8415 | KAE8793100.1 (*Hordeum vulgare*) | plastid glutamine synthetase isoform GS2c, chloroplastic | | 96 | 12 | 5.75/46.68 | 7.73/46.68 | 15, 18 ↓ | | | |
| Carbohydrate metabolism | | | |  |  |  |  |  | | | |
| 5401 | XP_020173165.1(*Aegilops tauschii* ssp. *strangulata*) | UDP-glucose 6-dehydrogenase 4 | | 636 | 26 | 5.84/52.6 | 6.76/54.43 | 3, 11, 18, 23 ↑ 7, 8, 9 ↓ | | | |
| 8525 | XP_020186567.1(*Aegilops tauschii* ssp. *strangulata*) | UTP--glucose-1-phosphate uridylyltransferase | | 561 | 25 | 5.2/51.66 | 7.56/53.2 | 12, 14 ↑ 20 ↓ | | | |
| 9410 | XP_020148641.1 (*Aegilops tauschii* ssp. *strangulata*) | fructokinase-2 | | 391 | 23 | 5.06/35.55 | 7.79/40.02 | 2, 17 ↑ 5, 6, 7, 8, 11, 22, 24 ↓ | | | |
| Energy metabolism | | | |  |  |  |  |  | | | |
| ATP metabolism | | |  |  |  |  |  |  | | | |
| 3306 | XP_040248654.1 (*Aegilops tauschii ssp strangulata*) | ATP synthase subunit alpha, mitochondrial-like | | 476 | 21 | 6.02/55.26 | 6.7/36.04 | 8, 9, 12, 13, 15, 16, 18, 20, 21, 22 ↑ 3 ↓ | | | |
| Glycolysis/gluconeogenesis | | | |  |  |  |  |  | | | |
| 1206 | ABQ81648.1 (*Triticum aestivum*) | glyceraldehyde-3-phosphate dehydrogenase | | 559 | 31 | 7.09/36.64 | 5.69/42.05 | 1, 2, 3, 16, 17, 18 ↑ | | | |
| 2503 | EMS54975.1 (*Triticum urartu*) | Glyceraldehyde-3-phosphate dehydrogenase, cytosolic | | 177 | 13 | 9.17/42.57 | 5.84/43.66 | 16, 17 ↓ | | | |
| 2205 | ANW11922.1 (*Triticum aestivum*) | glyceraldehyde-3-phosphate dehydrogenase, partial | | 600 | 30 | 6.74/36.27 | 6.02/43.24 | 23, 24 ↑ 18 ↓ | | | |
|  |  |  | |  |  |  |  |  | | | |
| 7608 | XP_037414566.1 (*Triticum dicoccoides*) | 2,3-bisphosphoglycerate-independent phosphoglycerate mutase-like | | 211 | 25 | 5.37/60.63 | 7.2/60.6 | 4, 23 ↓ | | | |
| Photosynthesis | | | |  |  |  |  |  | | | |
| 2618 | AAU11113.1  (*Psathyrostachys huashanica*) | ribulose-1,5-bisphosphate carboxylase/oxygenase large subunit (chloroplast) | | 422 | 28 | 6.13/52.86 | 6.19/53.61 | 5, 6, 11, 14, 24 ↑1, 18 ↓ | | | |
| 3620 | AAU11113.1 (*Psathyrostachys huashanica*) | ribulose-1,5-bisphosphate carboxylase/oxygenase large subunit (chloroplast) | | 343 | 25 | 6.13/52.86 | 6.33/53.62 | 2, 3, 5, 6, 11, 12, 22, 24 ↑ 1, 7, 8 ↓ | | | |
| 4011 | KAE8790036.1 (*Hordeum vulgare*) | chloroplast ribulose-1,5-bisphosphate carboxylase/oxygenase small subunit | | 351 | 15 | 8.58/19.47 | 6.48/13.11 | 3, 5, 6, 22, 24 ↑ 15, 18, 20, 21 ↓ | | | |
| 3405 | ASD37885.1 (*Aegilops* | ribulose 1,5-bisphosphate | | 396 | 26 | 6.22/52.32 | 6.4/53.61 | 3, 12 ↑ 15, 17, 18, 21 ↓ | | | |
|  | *speltoides*) | carboxylase/oxygenase large subunit | |  |  |  |  |  | | | |
| Respiration | | | |  |  |  |  |  |  | |  |
| 2108 | XP_020198028.1 (*Aegilops tauschii* ssp. *strangulata*) | NAD(P)H dehydrogenase (quinone) FQR1 | | 412 | 17 | 6.21/21.78 | 6.03/26.01 | 3, 6 ↑ 22, 24 ↓ |  | |  |
| 2506 | XP_037446710.1 (*Triticum dicoccoides*) | citrate synthase 4, mitochondrial-like | | 204 | 16 | 6.88/52.57 | 5.96/49.41 | 8 ↑ 12 ↓ |  | |  |
| 5103 | XP_037473639.1 (*Triticum dicoccoides*) | malate dehydrogenase, mitochondrial-like | | 250 | 10 | 8.57/35.48 | 6.79/38.8 | 15, 21, 23, 24 ↓ |  | |  |
| 5103 | KAE8779219.1 (*Hordeum vulgare*) | electron transfer flavoprotein subunit alpha, mitochondrial | | 148 | 14 | 6.41/37.4 | 6.79/38.8 | 15, 21, 23, 24 ↓ |  | |  |
| Protein folding | | | |  |  |  |  |  |  | |  |
| 8629 | XP_020147244.1 | RuBisCO large | | 496 | 26 | 5.56/63.95 | 7.5/59.31 | 5, 6, 14, 19, 22, 24 ↑ |  | |  |
|  | (*Aegilops tauschii ssp strangulata*) | subunit-binding protein subunit beta, chloroplastic | |  |  |  |  |  |  | |  |
| Stress and defense | | | |  |  |  |  |  | |  |  |
| 3005 | QPO15911.1 (*Leymus chinensis*) | late embryogenesis abundant protein 19 | | 323 | 13 | 6.32/16.18 | 6.35/23.28 | 3 ↑ 4, 5, 19 ↓ | |  |  |
| 3111 | AAG00428.1 (*Hordeum vulgare*) | germin D | | 77 | 5 | 6.02/24.47 | 6.1/25.93 | 4, 7, 8, 9, 12, 13, 18 ↑ 1, 2, 3, 22, 23 ↓ | |  |  |
| 3207 | XP_037414204.1  (*Triticum dicoccoides*) | glutathione S-transferase 1-like | | 360 | 15 | 5.79/23.39 | 6.33/26.88 | 1, 10 ↑ 4, 5, 13, 16, 17 ↓ | |  |  |
| 4101 | XP_020196644.1  (*Aegilops tauschii* ssp. *strangulata*) | probable glutathione S-transferase DHAR1, cytosolic | | 880 | 28 | 9.11/33.77 | 6.59/26.71 | 4, 5, 7, 8, 12, 13, 15, 17, 18, 19, 21 ↑ 3↓ | |  |  |
| 4519 | EMS50129.1 (*Triticum urartu*) | Heat shock cognate 70 kDa protein 1 | | 224 | 26 | 5.16/80.91 | 6.57/50.08 | 15, 20, 21 ↑ 5, 13 ↓ | |  |  |
| 5304 | XP_037439714.1  (*Triticum dicoccoides*) | heat shock cognate 70 kDa protein 2-like | | 407 | 28 | 5.16/80.91 | 6.84/50.87 | 9, 11, 15, 20 ↑ 3, 5, 6, 14, 17, 19 ↓ | |  |  |
|  |  |  | |  |  |  |  |  | |  |  |
| 6106 | XP_037460771.1 (*Triticum dicoccoides*) | germin-like protein 8-14 | | 222 | 6 | 5.36/21.79 | 7.16/25.64 | 4, 12 ↑14, 18 ↓ | |  |  |
| 8711 | XP_037444157.1 (*Triticum dicoccoides*) | heat shock cognate 70 kDa protein 2-like | | 292 | 27 | 5.08/71.16 | 7.48/72.66 | 4, 5, 12, 23 ↑ 1, 18 ↓ | |  |  |
| 9601 | XP_037439714.1 (*Triticum dicoccoides*) | heat shock cognate 70 kDa protein 2-like | | 405 | 29 | 5.06/71.07 | 7.77/74.91 | 11 ↑ 15, 18 ↓ | |  |  |
| Redox metabolism | | | |  |  |  |  |  | |  |  |
| 3111 | QBZ38485.1 (*Triticum monococcum*) | MnSOD, mitochondrial | | 238 | 19 | 7.89/25.35 | 6.1/25.93 | 4, 7, 8, 9, 12, 13, 18 ↑ 1, 2, 3, 22, 23 ↓ | |  |  |
| 3406 | XP_037475587.1 (*Triticum dicoccoides*) | L-ascorbate peroxidase 2, cytosolic-like | | 159 | 10 | 5.1/27.67 | 6.51/53.65 | 2 ↑ 6, 14, 16, 22, 24 ↓ | |  |  |
| 3503 | ADJ67791.1 (*Triticum aestivum*) | aldehyde dehydrogenase 7b | | 476 | 20 | 5.9/54.35 | 6.54/55.55 | 5, 23, 24 ↑ | |  |  |
|  |  |  | |  |  |  |  |  | |  |  |
| 3803 | QNV69782.1 (*Triticum turgidum* ssp. *durum*) | lipoxygenase-2 | | 290 | 26 | 6.09/96.75 | 6.2/96.8 | 1, 10 ↑ | |  |  |
| 4207 | XP_020172367.1 (*Aegilops tauschii* ssp. *strangulata*) | L-ascorbate peroxidase 1, cytosolic | | 497 | 24 | 5.85/27.46 | 6.4/28.74 | 4, 8, 15, 20 ↑ 3, 6 ↓ | |  |  |
| 4519 | XP_020190543.1 (*Aegilops tauschii* ssp. *strangulata*) | Peroxidase 1 | | 325 | 13 | 6.3/37.67 | 6.57/50.08 | 15, 20, 21 ↑ 5, 13 ↓ | |  |  |
| 7013 | XP_037413838.1 (*Triticum dicoccoides*) | peroxiredoxin-2C-like | | 404 | 16 | 5.15/17.35 | 7.34/18.57 | 16, 18 ↑ 6 ↓ | |  |  |
| 8103 | XP_037475587.1 (*Triticum dicoccoides*) | L-ascorbate peroxidase 2, cytosolic-like | | 505 | 24 | 5.1/27.67 | 7.69/29.42 | 18 ↑ 7, 23, 24 ↓ | |  |  |
| 8106 | XP_037475587.1 (*Triticum dicoccoides*) | L-ascorbate peroxidase 2, cytosolic-like | | 432 | 13 | 5.1/27.67 | 7.6/28.62 | 3, 17 ↑ 9, 16, 20, 21, 22, 24 ↓ | |  |  |
| 8125 | AFF27606.1 (*Triticum aestivum*) | chloroplast Cu/Zn superoxide dismutase, partial | | 51 | 1 | 5.2/17.53 | 7.42/19.47 | 3, 5, 6, 14, 17 ↑ 15 ↓ | |  |  |
|  |  |  | |  |  |  |  |  | |  |  |
|  |  |  | |  |  |  |  |  | |  |  |
| Glutathione metabolism | | | |  |  |  |  |  | |  |  |
| 7413 | EMS48257.1 (*Triticum urartu*) | Protein IN2-1-like protein B | | 205 | 13 | 8.84/41.02 | 7.42/31.32 | 11 ↑ 17 ↓ | |  |  |
| Regulatory | | | |  |  |  |  |  | |  |  |
| 2206 | XP_037420774.1 (*Triticum dicoccoides*) | ricin B-like lectin R40C1 | | 657 | 36 | 6.24/38.63 | 6.03/39.48 | 24 ↑ | |  |  |
| 4411 | XP_020183874.1 (*Aegilops tauschii* ssp. *strangulata*) | ricin B-like lectin R40G3 | | 510 | 41 | 5.93/36.43 | 6.49/40.17 | 4, 5 ↑ 14, 16, 24 ↓ | |  |  |
| One-carbon metabolism | | | |  |  |  |  |  | |  |  |
| 5302 | sp\|A6XMY9.1\|METK1_TRIMO (*Triticum monococcum*) | S-adenosylmethionine synthase 1 | | 602 | 22 | 5.61/42.82 | 6.79/49.19 | 3 ↑ 8, 9, 11 ↓ | |  |  |
| 6522 | sp\|Q4LB22.1\|METK3_HORVU (*Hordeum vulgare*) | S-adenosylmethionine synthase 3 | | 626 | 17 | 5.52/42.76 | 7.06/49.47 | 3, 12 ↑ 5, 9, 21 ↓ | |  |  |
|  |  |  | |  |  |  |  |  | |  |  |
| Secondary metabolism | | | |  |  |  |  |  | |  |  |
| 6106 | XP_020157388.1 (*Aegilops tauschii* ssp. *strangulata*) | isoflavone reductase homolog IRL | | 254 | 14 | 5.31/33.12 | 7.16/25.64 | 4, 12 ↑ 14, 18 ↓ | |  |  |
| Sulfur metabolism | | | |  |  |  |  |  | |  |  |
| 12 | XP_040242492.1 (*Aegilops tauschii* ssp. *strangulata*) | CBS domain-containing protein CBSX3, mitochondrial | | 255 | 19 | 9.14/22.39 | 5.39/22.3 | 4, 7, 18 ↑ 1, 11, 12, 22, 24 ↓ | |  |  |
| Nucleotide metabolism | | | |  |  |  |  |  | |  |  |
| 4011 | EMS63724.1 (*Triticum urartu*) | Nucleoside diphosphate kinase 1 | | 51 | 1 | 6.31/16.58 | 6.48/13.11 | 3, 5, 6, 22, 24 ↑ 15, 18, 20, 21 ↓ | |  |  |
| Signalling | | | |  |  |  |  |  | |  |  |
| 4304 | KAE8791495.1 (*Hordeum vulgare*) | Serine/threonine-protein kinase 38-like protein | | 305 | 16 | 6.03/30.86 | 6.61/33.58 | 20 ↑ 10, 11, 19 ↓ | |  |  |
| 5103 | XP_037425140.1 (*Triticum dicoccoides*) | guanine nucleotide-binding protein subunit beta-like protein A | | 140 | 14 | 6.06/36.28 | 6.79/38.8 | 15 ↓ | |  |  |
|  |  |  | |  |  |  |  |  | |  |  |
| 6409 | XP_020197659.1 (*Aegilops tauschii* ssp. *strangulata*) | guanine nucleotide-binding protein subunit beta-like protein A | | 121 | 19 | 5.97/36.16 | 6.9/39.04 | 18, 20, 21 ↑13 ↓ | |  |  |
| Molecular transport | | | |  |  |  |  |  | |  |  |
| 110 | XP_037413079.1 (*Triticum dicoccoides*) | mitochondrial outer membrane protein porin 3 | | 546 | 16 | 6.79/29.9 | 5.35/29.9 | 3, 4, 5, 12 ↑ 8, 9 ↓ | |  |  |
| 202 | XP_020174223.1 (*Aegilops tauschii* ssp. *strangulata*) | GTP-binding nuclear protein Ran-2 isoform X2 | | 191 | 14 | 6.65/25.13 | 6.65/25.13 | 3 ↑ | |  |  |
| 3208 | XP_020174223.1 (*Aegilops tauschii* ssp. *strangulata*) | GTP-binding nuclear protein Ran-2 isoform X2 | | 255 | 12 | 6.65/25.13 | 6.15/30.3 | 13 ↑ 21, 24 ↓ | |  |  |
| Cytoskeleton | | | |  |  |  |  |  | |  |  |
| 6110 | XP_037436859.1 (*Triticum dicoccoides*) | actin-depolymerizing factor 4-like isoform X1 | | 552 | 24 | 6.45/21.46 | 6.97/17.05 | 10 ↑16, 18, 20 ↓ | |  |  |
| 6111 | XP_037436859.1 (*Triticum dicoccoides*) | actin-depolymerizing factor 4-like isoform X1 | | 457 | 18 | 6.45/21.46 | 6.98/18.33 | 18 ↑ 11, 22, 24 ↓ | |  |  |
|  |  |  | |  |  |  |  |  | |  |  |
| 8525 | Q9ZRB7.1 (*Triticum aestivum*) | Tubulin alpha chain | | 145 | 11 | 4.89/49.74 | 7.56/53.2 | 12, 14 ↑ | |  |  |
| Transcription and RNA processing | | | |  |  |  |  |  | |  |  |
| 202 | AAK01176.1 (*Triticum aestivum*) | RNA-binding protein | | 45 | 1 | 6.6/20.31 | 5.58/30.23 | 3 ↑ | |  |  |
| 6409 | XP_037412554.1 (*Triticum dicoccoides*) | transcription factor Pur-alpha 1-like | | 453 | 24 | 5.59/32.89 | 6.9/39.04 | 18, 20, 21 ↑ 13 ↓ | |  |  |
| 7008 | XP_020162185.1 (*Aegilops tauschii* ssp. *strangulata*) | glycine-rich RNA-binding protein blt801 | | 579 | 20 | 5.41/16.92 | 7.33/13.68 | 10, 22 ↑ 13, 15, 16, 17 ↓ | |  |  |
| 7608 | XP_037476932.1 (*Triticum dicoccoides*) | polyadenylate-binding protein RBP45-like isoform X2 | | 467 | 13 | 5.55/50.91 | 7.2/60.6 | 4, 23 ↓ | |  |  |
| 7413 | XP_020150997.1 (*Aegilops tauschii* ssp. *strangulata*) | nuclear cap-binding protein subunit 2 | | 189 | 14 | 5.42/28.04 | 7.42/31.32 | 11, 22 ↑ 17 ↓ | |  |  |
| Protein metabolism | | | |  |  |  |  |  | |  |  |
|  | | | |  |  |  |  |  | |  |  |
| 1206 | KAE8800160.1 (*Hordeum vulgare*) | eukaryotic translation initiation factor 3 subunit i | | 130 | 3 | 3.31/35.57 | 5.69/42.05 | 1, 2, 3, 16, 17, 18 ↑ | |  |  |
| 2014 | KAE8790324.1 (*Hordeum vulgare*) | 40S ribosomal protein S21 | | 296 | 10 | 6.71/9.12 | 6/9.12 | 7, 17, 18 ↑ 6, 11, 22, 24 ↓ | |  |  |
| 2210 | XP_020167947.1 (*Aegilops tauschii* ssp. *strangulata*) | proteasome subunit alpha type-6 | | 720 | 25 | 6.33/27.43 | 6.03/28.57 | 2, 16, 17, 18 $\uparrow$ | |  |  |
| 2602 | KAE8802615.1 (*Hordeum vulgare*) | mitochondrial-processing peptidase subunit alpha | | 146 | 15 | 6.74/53.48 | 6.04/52.83 | 3 ↑ 5, 8, 9 ↓ | |  |  |
| 3009 | XP_037432389.1  (*Triticum dicoccoides*) | ubiquitin-conjugating enzyme E2 variant 1D-like | | 207 | 19 | 6.42/16.66 | 6.21/17.97 | 2, 7, 24 ↑ 14 ↓ | |  |  |
| 5304 | EMS47104.1 (*Triticum urartu*) | 26S protease regulatory subunit 6B-like protein | | 130 | 4 | 5.91/41.45 | 6.84/50.87 | 9, 11, 15, 20 ↑ 3, 5, 6, 17, 19 ↓ | |  |  |
| 8106 | XP_037419561.1 (*Triticum dicoccoides*) | proteasome subunit alpha type-6-like | | 272 | 15 | 6.33/27.45 | 7.6/28.62 | 3, 17 ↑ 9, 16, 20, 21 ↓ | |  |  |
|  |  |  | |  |  |  |  |  | |  |  |
|  |  |  | |  |  |  |  |  | |  |  |
| 9409 | AQU14669.1  (*Triticum aestivum*) | ribosomal protein S2 | | 236 | 15 | 4.87/33.28 | 7.79/45.17 | 1, 7, 16 ↑ 5, 10, 11, 12, 22 ↓ | |  |  |
| Proteins with unknown function | | | |  |  |  |  |  | |  |  |
| 2013 | KAE8780228.1 (*Hordeum vulgare*) | hypothetical protein D1007_46620 | | 102 | 6 | 6.33/14.71 | 6.15/11.62 | 3, 4, 5, 7, 12, 24 ↑ 9 ↓ | |  |  |
| 4522 | XP_037439716.1  (*Triticum dicoccoides*) | uncharacterized protein LOC119307737 | | 85 | 5 | 7.68/17.62 | 6.51/46.48 | 4, 12 ↑ 6 ↓ | |  |  |
| 6113 | XP_037458661.1 (*Triticum dicoccoides*) | uncharacterized protein LOC119329692 | | 262 | 13 | 5.58/16.88 | 7.06/18.94 | 3 ↑ 15 ↓ | |  |  |
| 8125 | XP_037446867.1 (*Triticum dicoccoides*) | uncharacterized protein At2g34160-like | | 148 | 6 | 5.18/15.17 | 7.42/19.47 | 3, 5, 6, 14, 17 ↑ 15 ↓ | |  |  |
| 9603 | VAI28258.1 (*Triticum turgidum* ssp. *durum*) | unnamed protein product | | 107 | 18 | 5.03/67.29 | 7.8/74.6 | 1, 7, 8, 15, 16, 18, 20, 23, 24 ↓ | |  |  |
